# Characteristics of phosgene aspiration lung injury analyzed based on transcriptomics and proteomics

**DOI:** 10.3389/fgene.2024.1393665

**Published:** 2024-05-17

**Authors:** Li-Na Wang, Yi-Ru Shao, Peng-Fei Wang, Jiang Lv, Dai-Kun He

**Affiliations:** ^1^ Department of General Practice, Jinshan Hospital, Fudan University, Shanghai, China; ^2^ Center of Emergency and Critical Medicine, Jinshan Hospital, Fudan University, Shanghai, China; ^3^ Research Center for Chemical Injury, Emergency and Critical Medicine of Fudan University, Shanghai, China; ^4^ Key Laboratory of Chemical Injury, Emergency and Critical Medicine of Shanghai Municipal Health Commission, Shanghai, China

**Keywords:** transcriptomics, proteomics, phosgene, inhalation lung injury, influencing factors

## Abstract

**Background:**

Phosgene is a chemical material widely used worldwide. No effective method has been developed to reverse its pathological injuries. Some studies have shown that neuronal inflammation in lung tissue is involved, but the specific mechanism has not been reported.

**Objective:**

To analyze the expression alterations of whole transcriptome gene sequencing bioinformatics and protein expression profile in lung tissue after phosgene aspiration lung injury (P-ALI) and find the main factors and pathways affecting the prognosis of P-ALI.

**Methods:**

Rat models of P-ALI were made by phosgene. Rats were divided into a P-ALI group and a blank group. Hematoxylin-eosin (HE) staining and lung wet/dry ratio measurement were used to evaluate the lung injury. The levels of inflammatory factors were measured by ELISA. High-throughput sequencing was used to measure the expression profile of each gene. Protein expression profiles were determined by label-free relative quantification of the differential proteome.

**Results:**

Lung injury such as the disordered structure of alveolar wall and inflammatory factors (IL-1β, IL-18, and IL-33) were significantly increased in the P-ALI group (*p* < 0.05). There were 225 differentially expressed lncRNAs, including 85 upregulated and 140 downregulated genes. They were also the genomes with the most significant changes in transcriptome gene expression, mainly constituting cytoplasmic, synaptic structures and transporters, and involved in amino acid and carbon metabolism. There were 42 differentially expressed circRNAs, including 25 upregulated genes and 17 downregulated genes, mainly involved in cell composition, growth, differentiation, and division. There were only 10 differentially expressed miRNAs genes, all upregulated and mainly involved in the inflammatory response pathway. Proteome identification showed 79 differentially expressed proteins. KEGG enrichment analysis showed that it was mainly involved in the N-glycan biosynthesis pathway.

**Conclusion:**

We discovered that differentially regulated genes (lncRNAs, circRNAs, and miRNAs) were primarily associated with neuronal reflexes and synaptic signaling, including neurotransmitter transmission, ion signaling pathway conduction, neuronal projection, and synaptic vesicle circulation. They affected inflammatory factors and other metabolic pathways. This finding could be explored in future studies.

## 1 Introduction

Phosgene (COCI_2_) was first synthesized in 1812, and it can react with water to produce carbon dioxide (CO_2_) and hydrochloric acid (HCL) slowly, which is a toxic gas with irritating effects on the respiratory tract. Phosgene was used in chemical weapons as a critical chemical intermediate, causing 80% of chemically related deaths during the First World War (Holmes et al., 2016). In modern society, it is widely used in the chemical production process of dyes, pesticides, drugs, disinfectants, cleaners, and fertilizers, so it has a wide range of industrial uses worldwide. However, phosgene in production, storage, and transportation in any link is an improper operation or the risk of accidental leakage. As a result, exposed individuals inhale phosgene, the primary cause of acute lung injury, hypoxemia, respiratory acidosis, hemolysis (Aggarwal et al., 2019), serious edema, and acute respiratory distress syndrome (Aggarwal et al., 2016; Aggarwal et al., 2018). They will also gradually develop chronic lung diseases, such as pulmonary fibrosis (Mo et al., 2013), airway hyperresponsiveness, and gas exchange disorders (Song et al., 2011; Balakrishna et al., 2014), which seriously threaten people’s health and life safety. In general, there are many biological factors involved in the inflammatory response, among which IL-1β, IL-18, and IL-33 are actively expressed in lung tissue inflammation, and their levels are often elevated when different pathogens such as bacteria, viruses, and parasites are infected (Sager et al., 2014; Carreto-Binaghi et al., 2021; Hawerkamp et al., 2023).There is no effective treatment for phosgene-induced respiratory system injury, and oxygen therapy, glucocorticoids, and symptomatic treatment remain the primary clinical treatments. Therefore, effective treatment strategies are urgently needed. Many researchers became interested in the mechanism of phosgene-induced acute lung injury to address this issue.

In the past few decades, transcription of omics research to make people realize the transcription factors may be involved in all aspects of the immune response and inflammatory reaction, such as immune cell differentiation and development, RNA-Seq technology has been increasingly used in the diagnosis of various diseases such as single gene disease (Saeidian et al., 2020), cardiac muscle myopathy (Meng et al., 2019), blood system diseases (Arindrarto et al., 2021), etc., showing the strong ability of analysis and supplementary diagnosis. In addition, the noncoding RNAs are also closely related to human diseases, including lncRNAs, circRNAs, microRNAs, and other RNAs with known and unknown RNAs.

With the development of high-throughput sequencing technology (also known as next-generation sequencing technology), it is considered a promising next-generation precision medicine technology that can rapidly, comprehensively, and massively analyze the transcriptome and genome of a species in detail. Therefore, we aim to explore the potential key cellular pathways and functions affecting phosgene aspiration lung injury (P-ALI) through transcriptomic sequencing technology, combined with proteomic identification and bioinformatics data analysis to screen critical prognoses to provide new directions and targets for clinical research and treatment.

## 2 Materials and methods

### 2.1 Animals

In our study, the number of animals in each group was set at 3, based on the 4R principle (reduction, refinement, replacement, and responsibility) advocated in animal experiments and a large amount of literature summary. Six healthy male adult Sprague-Dawley (SD) rats used in this study were purchased from the Animal Experimental Center of Shanghai Second Military Medical University. All rats were 4–6 weeks old, free of the specific pathogen (SPF), and weighing 180–280 g (certificate number: 20180004049887). All rats were exposed to alternating light/dark cycles for 12 h at a room temperature of 20°C–24°C and were free to move around and obtain food and water by themselves.

We anesthetized rats by intraperitoneal injection of pentobarbital sodium at a dose of 150–200 mg/kg. Our animal tests were approved by the Animal Ethics Committee of Shanghai Public Health Clinical Center Laboratory Animal Welfare (No. 2020-A036-001) and the animals in this study were disposed of in strict accordance with the European Union Code for Animal Management (1986) recommendations.

### 2.2 Preparation and grouping of the rat model of phosgene inhalation lung injury

The experimental rats were divided into an air group (control group) and a phosgene exposure group (P-ALI group). The rats were exposed to the poison cabinet for 5 min and then quickly removed and placed in the fresh air. In this process, the dynamic poison cabinet was purchased from Shanghai Second Military Medical University. The rats in the air group were also placed in the exposure cabinet with the same airflow as the rats in the exposure group. All other procedures were the same. Rats in the air group were numbered 1, 2, and 3, and rats in the P-ALI group were numbered 4, 5, and 6. The phosgene used in the experiment was prepared by 40% phosgene organic alkali titration. The steps were as follows: 1.8 g solid triphosgene (COCl_2_, molecular weight 98.95) was dissolved in 5 mL cyclohexane to make 40% concentrated phosgene; 20 mL N, N-dimethylformamide (C_3_H_7_NO, DMF) was slowly dropped to produce phosgene gas. At this time, the gas concentration was 8.33 mg/m^3^. The air group was exposed to normal room air, while the phosgene group was exposed to phosgene gas at a constant rate in the airtight cabinet for 5 min until a final concentration of 8.33 mg/L, as previously described. The room temperature was 24°C and the humidity was 55%–60%. Besides, all the other environmental conditions were same to maintain the airflow rate and phosgene concentration.

### 2.3 Pathological changes in lung tissue

In our test, we euthanized the rats with an overdose of anesthesia. As rapid overdose of anesthetics caused excessive central nervous suppression in rats leading to death, we anesthetized rats by intraperitoneal injection of pentobarbital sodium at a dose of 150–200 mg/kg. During intraperitoneal injection, the needle should be drawn back after entering the animal’s abdominal cavity to avoid injecting anesthetic into the animal’s gastrointestinal tract. After the rats were deathed by overdose, the right middle lobe of each group was taken, fixed with 10% paraformaldehyde, embedded in paraffin at low temperature, sectioned, and stained with hematoxylin-eosin (HE). The pathological changes of the lung tissue were observed under the light microscope.

### 2.4 Determination of lung wet-to-dry ratio

The wet-to-dry ratio (W/D) usually reflects the water content of lung tissue and the degree of pulmonary edema. Therefore, after thoracotomy, the right lower lungs of the rats were removed to weigh the wet weights, and then the lungs were roasted in an oven (80°C, 48 h) to constant weight to weigh the dry weights. Finally, the wet-to-dry ratio (W/D) of lung tissue was calculated.

### 2.5 Determination of neutrophil count and protein content in BALF

In each group, 5 mL of normal iced saline (4°C) was absorbed by syringe through the right bronchial puncture cannula, and the normal saline was slowly injected into the right upper lung with a small pressure through the endotracheal tube. The bronchoalveolar lavage fluid (BALF) was pumped back, and so on, repeatedly until the total amount of BALF reached 15 mL, and smears of blood cell counting plate counted the cells. The resulting lavage solution was centrifuged (1000 r/min, 10 min), and the supernatant was collected in a 15 mL centrifuge tube and frozen at −80°C. The supernatant was used for protein determination.

### 2.6 Determination of IL-1β, IL-18, and IL-33 in serum and BALF

The contents of IL-1β, IL-18, and IL-33 in serum and BALF were determined by double antibody sandwich enzyme-labeled immunoassay (ELISA). The instructions for each kit were followed strictly. The absorbance value of the enzyme-labeled solubility wavelength was read at 450, and the corresponding absorbance value was calculated according to the standard curve.

### 2.7 Whole transcriptome sequencing

Next-Generation Sequencing (NGS) technology was used. Total RNA extractions included mRNA isolation, library building reagent, quantification, library recovery, bridge amplification, and onboard sequencing. The analysis process included data generation, data de-hybridization, transcriptome splicing, SSR analysis and SNP analysis, gene function annotation, gene expression differential analysis, differential gene expression pattern clustering, differential gene enrichment analysis, and the differential genes with the most significant possible correlation were selected. Total RNAs were isolated from the rat’s lung tissues using the Magzol Reagent (Magen, China)according to the manufac-turer’s protocol. The quantity and integrity of RNA syield were assessed by using the K5500(BeijingKaiao, China) and the Agilent 2200 TapeStation (Agilent Technologies, United States) separately. Based on the sequencing index design and results of different RNAs, the *p*-value of RNAs in different groups were calculated in subsequent analysis, and *p*-value<0.05 and |log2FoldChange| >1 were used as the definition criteria for differential RNAs.

### 2.8 Expression difference analysis and GO and KEGG enrichment analysis

Gene ontology (GO) analysis provides three aspects of molecular function, biological process, and cellular component for differentially expressed genes. First, cell composition refers to each part of the cell and the extracellular environment. Molecular functions mainly refer to the main activities of gene products at the molecular level, such as binding and catalysis. Finally, a biological process is an event or action inside a cell and defines its beginning and end. According to the GO annotation of genes, all genes of this species were selected as background genes, and the hypergeometric distribution method was used to calculate the *p*-value. With *p* < 0.05 as the significance threshold, high-frequency annotations with statistical significance relative to the background were obtained to obtain the distribution information and significance of gene sets in the GO category.

The significant difference in gene expression [|log2(FoldChange)|>1 and *p* < 0.05] was performed with KEGG analysis. KEGG pathway annotation provided the annotation information of signal transduction and disease pathways for differentially expressed genes to provide the background knowledge of gene pathways and function research. Therefore, enrichment analysis was performed to calculate the *p*-value by the Fisher Exact Test. *p* < 0.05 was taken as the significance threshold to obtain the signal transduction and disease pathways with statistical significance relative to the background to obtain the distribution information and significance of gene sets in KEGG categories.

### 2.9 Relative quantification of non-labeled differential proteome (label-free technique)

The two groups of samples were subjected to protein sample extraction, enzymatic hydrolysis, purification, mass spectrometry, and other processes. First, the protein was extracted. It was followed by protein purification, enzymatic hydrolysis, peptide purification, and other steps. Then, the sample was sent for a mass spectrometry test. After obtaining the mass spectrometry test data of different samples, peptide matching and signal intensity information extraction data analysis were performed, and finally, the relative expression amount of protein in different samples was obtained. After the quantitative information of proteins was processed, the following proteins were screened out: the detection values were missing in more than 50% of the samples or the quantitative values were consistent in all samples, and these proteins were removed in the subsequent quantitative analysis. The quantitative value of the remaining protein replaces the missing value with 1/5 of the smallest positive value in the original data. The sum of intensity values in each sample was normalized to obtain the quantitative information of the final protein. The difference protein was more than 2 times changed and *p* < 0.05 was used as the difference selection criterion. In this process, the quantitative protein-dye was Coomassie Plus Protein Assay, Thermo (No. 23236), and the extracted protein concentration was determined by the Bradford method [Marion M. Bradford], Analytical Biochemistry, 1976, 72: 248-254], and LC-MS mass spectrometry (instrument model: Fusion Mass spectrometry). The database used in this study was the Uniprot_RAT protein Library database, and Maxquant software was used for the database search. Differential protein GO analysis included three ontologies, which described the cellular location (CC), molecular function (MF) of genes, and biological process (BP).

### 2.10 Statistical analysis

SPSS 21.0 and GraphPad Prism 5.0 were used for data analysis, and we used Adobe Illustrator (AI) software for image processing. Measurement data conforming to normal distribution were expressed as mean ± standard deviation (‾x ± s), and two groups were compared by *t*-test. Non-normally distributed measurement data were expressed as median M and interquartile distance Q (P25, P75), and comparison between the two groups was performed by non-parametric Mann-Whitney *U* test. *p* < 0.05 was considered statistically significant.

## 3 Results

### 3.1 Analysis of the general state of rats

After exposure for 5 min, the rats in the P-ALI group showed restlessness, repeated jumping, gradually hobbled gait, significantly decreased mobility, half-closed eyes, drooping, curled up into a ball, anorexia, vertical hair, eyeball depression, shortness of breath, and indifference to external stimuli. The rats in the air group showed normal performance. All rats in the two groups survived without death. The P-ALI rat model was successfully replicated in our study. Further analysis showed that there were significant differences in pathological changes of lung tissue, wet/dry ratio and inflammatory markers between the air group and phosgene group.

#### 3.1.1 Histopathological changes in lung

Macroscopic observation showed that the lung tissue of rats in the air group had a smooth surface and intact structure, showing no signs of injury. Besides, the lung volume of rats in microscopic observation showed that the alveolar wall of rats in the air group was thin, the structure was intact, and there was no inflammatory cell infiltration in the alveolar wall and interstitium. However, the structure of the alveolar wall of rats in the P-ALI group was disordered, with clear congestion, edema, and thickening. The alveolar cavity was fused, the septum was widened, and a large number of inflammatory cells infiltrated. It can be seen that phosgene damage to lung tissue was so obvious.

#### 3.1.2 HE staining

**FIGURE 1:**
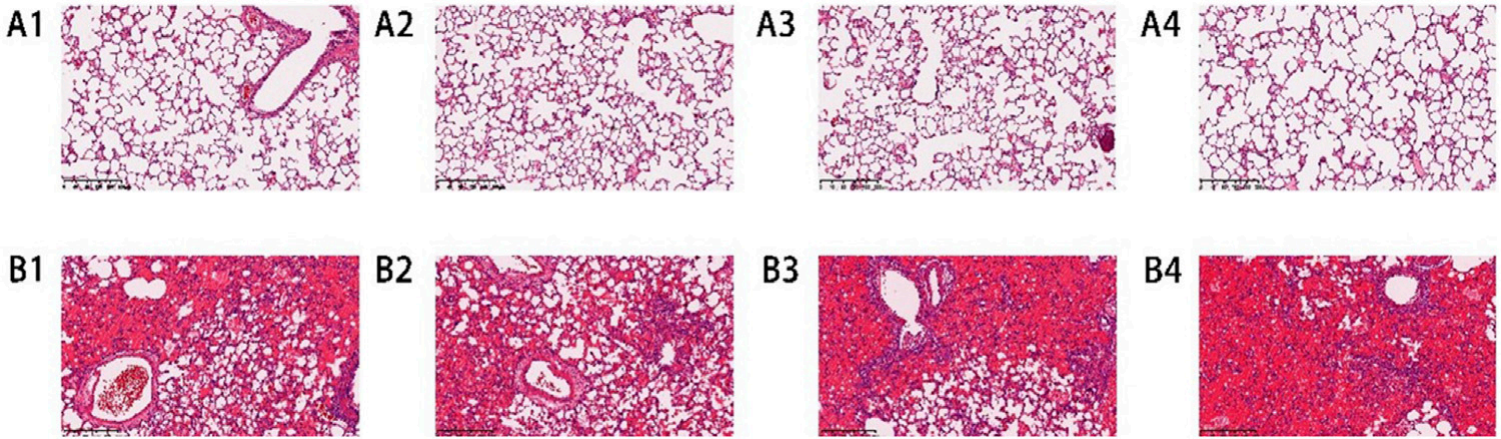
HE staining results of lung tissue slices of rats in the air group and P-ALI group. HE staining showed that the structure of the alveolar wall of the air group was complete, and there was no apparent inflammatory cell infiltration in the alveolar wall and interstitium (A1-A4). In the P-ALI group, the structure of the alveolar wall of the lung tissue was disordered, and a large number of inflammatory cells were infiltrated in the alveolar wall and interstitium, so the congestion and exudation were obvious (B1-B4).

Lung tissue samples were fixed with 4% paraformaldehyde for HE staining. Compared with the air group, the lung tissue of rats in the P-ALI group was significantly congested and exudated, and the inflammatory reaction was evident (Figure 1).

#### 3.1.3 Determination of the wet-to-dry ratio of lung and neutrophil count and protein content in BALF of rats

**FIGURE 2:**
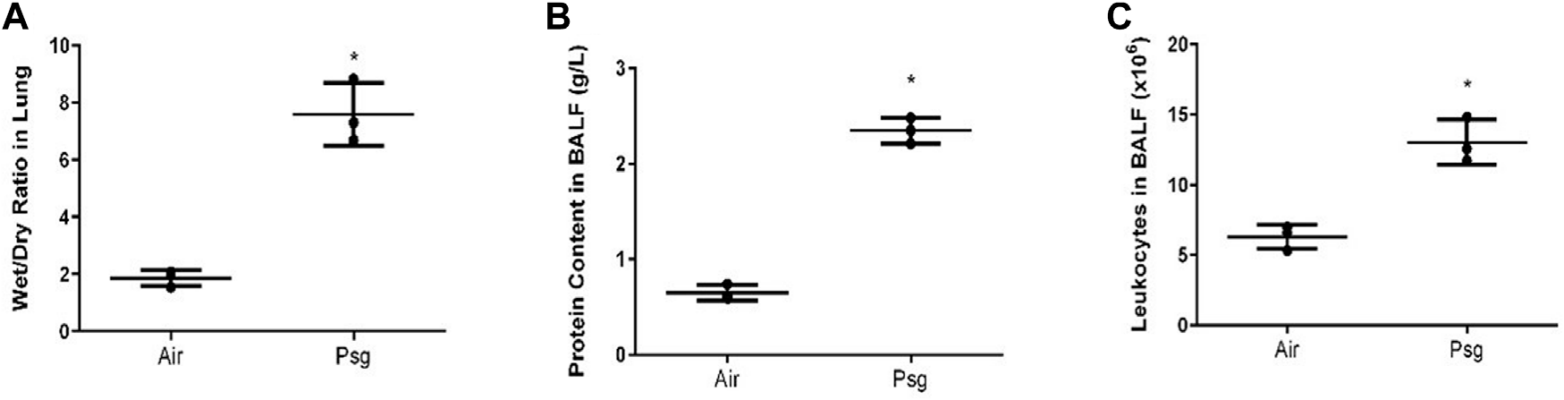
Changes of lung wet/dry ratio **(A)**, number of neutrophils **(C)**, and protein content in BALF **(B)** between air group and P-ALI group. The results showed that the lung wet/dry ratio, the number of neutrophils, and protein content in BALF rats in the P-ALI group were significantly higher than those in the air group, and the difference between the two groups was statistically significant (*p* < 0.05). Note. * indicates statistical significance between the two groups.

The lung W/D ratio of rats in the P-ALI group was significantly higher than that in the air group, and the difference was statistically significant (*p* = 0.00790, *p* < 0.05). Usually, the number of cells and protein content in BALF of rats was small. However, in this study, a large number of granulocytes were seen in the BALF of the P-ALI group when compared to the air group, and the protein content was significantly increased, the difference was statistically significant, too (*p* = 0.00126, 0.00050; *p* < 0.05) (Figure 2). This was closely related to the local inflammatory response.

#### 3.1.4 Comparison of IL-1β, IL-18, and IL-33 contents in serum and BALF

**FIGURE 3:**
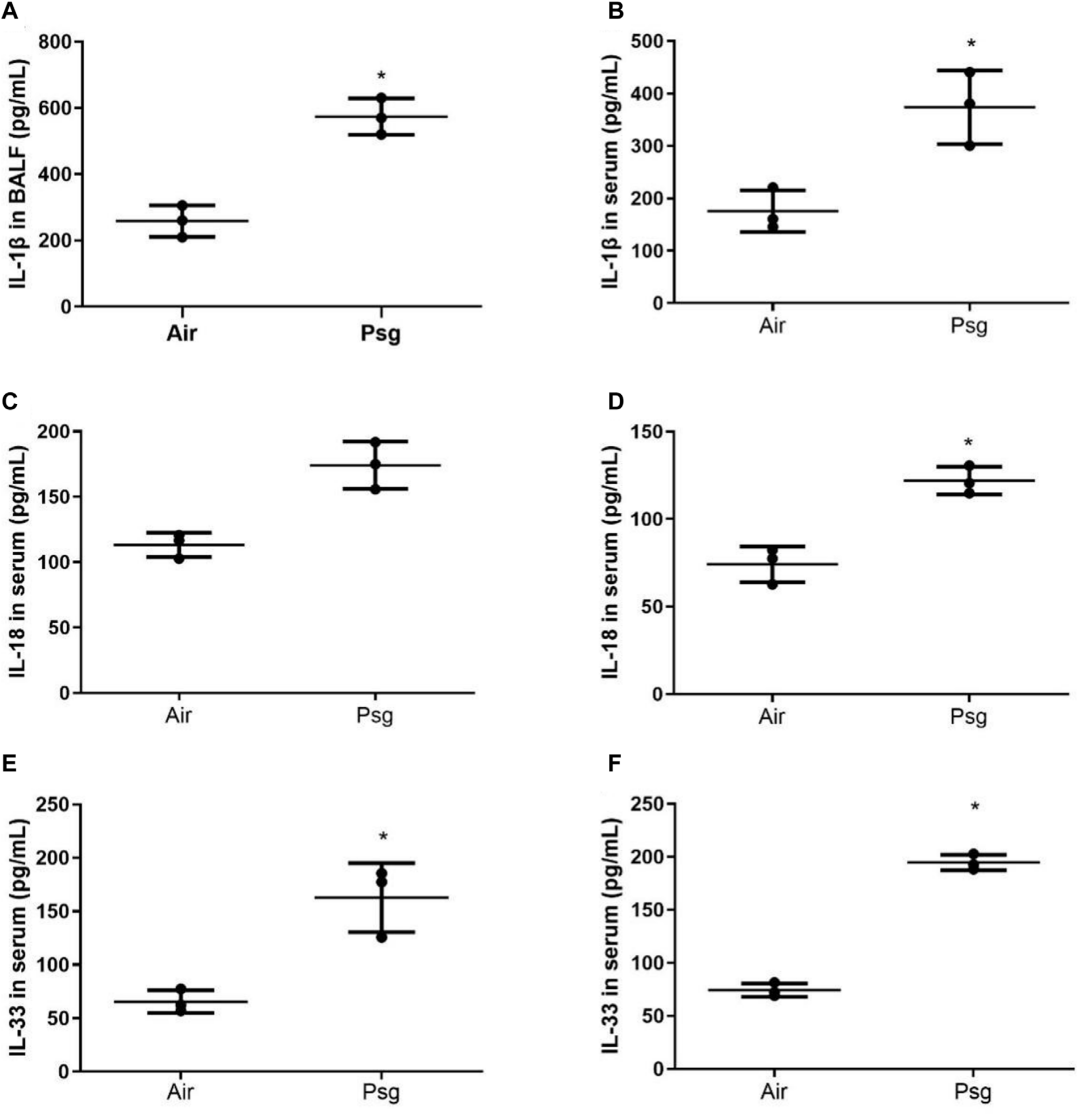
Comparison of IL-1β, IL-18, and IL-33 contents in serum and BALF of rats in air group and P-ALI group. Note. * indicates statistical significance between the two groups.

Compared with the air group, the contents of IL-1β, IL-18, and IL-33 in serum and BALF in the P-ALI group were significantly increased (*p* < 0.05) (Figure 3). The above experimental structure indicated that the P-ALI rat model was successfully reproduced and stable, which met the experimental requirements. Such IL-1β, IL-18, and IL-33 were common inflammatory factors, our study showed that all of these could be increased during P-ALI, and even leading to respiratory failure.

### 3.2 Whole transcriptome sequencing results

#### 3.2.1 lncRNAs sequencing results

**FIGURE 4:**
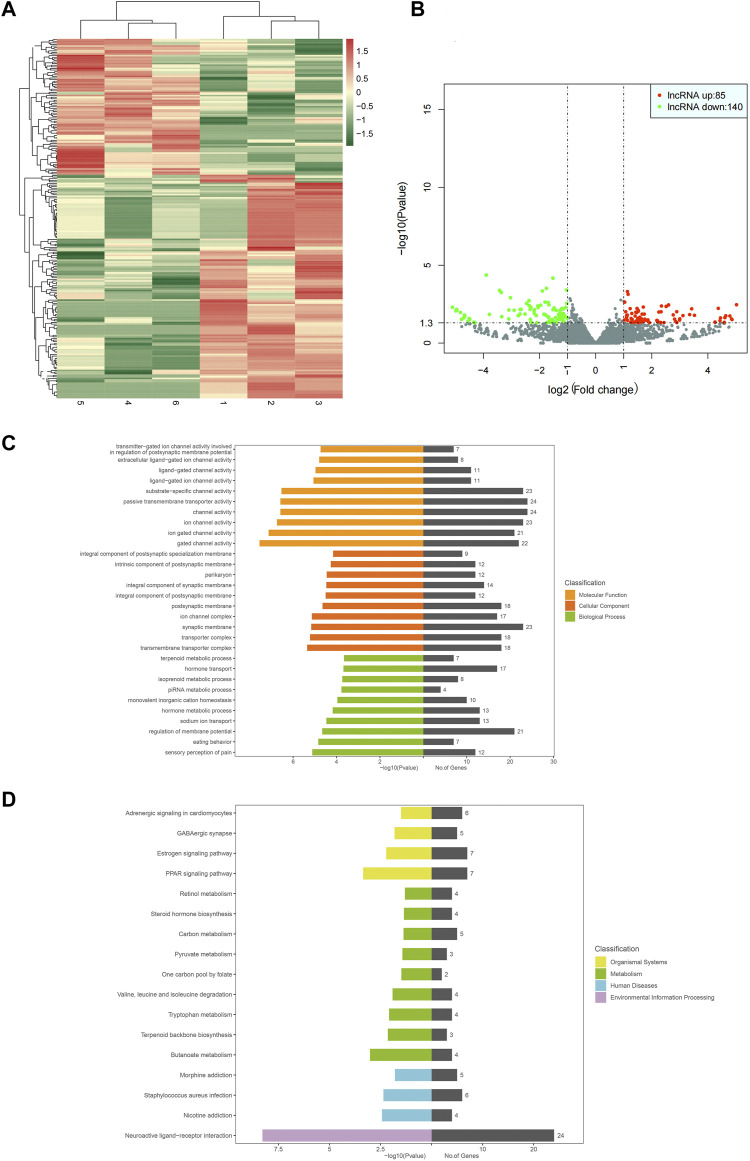
Heat map for analysis of gene differential expression between samples (control-treatment), color represents log10 (expression value + 1) value **(A)**. In the control-treatment map for analysis of gene differential expression between samples, the horizontal axis represents the change of gene expression multiple in different samples, and the vertical axis represents the statistical significance of the gene expression change. The red dots represent significantly upregulated genes, and the green represents significantly downregulated genes **(B)**. GO enrichment map (control-treatment) **(C)**. KEGG pathway enrichment map (control-treatment) **(D)**.

High-throughput sequencing results showed 10760 lncRNAs and 225 differentially expressed genes, including 85 upregulated and 140 downregulated genes. GO functional annotation analysis showed that the cellular components were mainly involved in the compositions of cytoplasm, synaptic structure, and transporters, including perikaryon, synaptic membrane, transmembrane transporter complex, and ion channel complex. Ion channel activities were involved in the most obvious molecular function enrichments, including channel activity, passive transmembrane transporter activity, ion channel activity, gated channel activity, ligand-gated channel activity, etc. The biological process was mainly involved in regulating membrane potential, while others were involved in hormone transport, hormone metabolism, sodium ion transport, and pain production (hormone transport, metabolic hormone process, sodium ion transport, sensory perception of pain, etc. KEGG pathway analysis showed that differential lncRNAs were mainly involved in amino acid and carbon metabolism, while environmental processes were mainly involved in neuroactive ligand-receptor interaction. In addition, these differential lncRNAs were also involved in human diseases, such as chronic morphine poisoning, morphine addiction, *staphylococcus aureus* infection, and nicotine addiction. The heat map, volcano map, and signal transduction pathways were shown in Figure 4.

#### 3.2.2 circRNAs sequencing results

**FIGURE 5:**
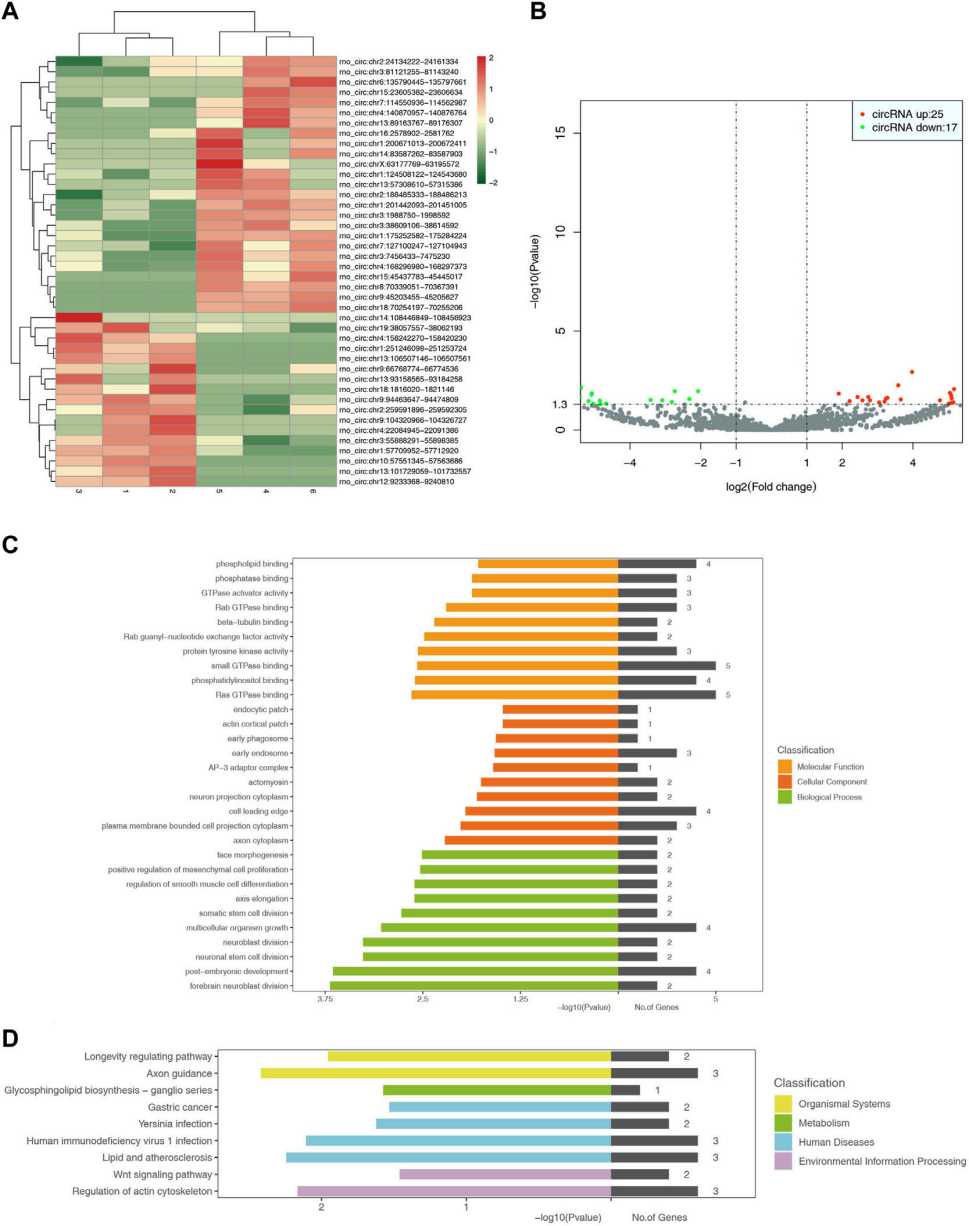
Heat map for analysis of differential expression of circRNAs between samples (control-treatment), color represents log10 (expression value + 1) value **(A)**. In the control-treatment diagram for the analysis of differential expression of circRNAs between samples, the horizontal axis represents the change of expression multiple of circRNAs in different samples, the vertical axis represents the statistical significance of the change of circRNAs expression, and the red dots represent the cirRNAs with significant differences **(B)**. GO enrichment map (control-treatment) **(C)**. Figure D shows the enrichment pattern of the KEGG pathway of differentially expressed genes (control-treatment) **(D)**.

The screening results showed 3862 circRNAs, including 42 differential cRNAs, 25 upregulated genes, and 17 downregulated genes. GO functional annotation analysis showed that the cellular components were mainly involved in cell composition, including the cell leading edge, neuron projection cytoplasm, and the membrane-bounded cell projection cytoplasm. It was also involved in the early phagosome, endosome, and actomyosin. The molecular function was mainly involved in the phosphorylation process in biochemical reactions, such as phospholipid binding, phosphatase binding, phosphatidylinositol binding, etc. The biological process was mainly involved in cell growth, differentiation, and division, including post-embryonic development, multicellular organism growth, somatic stem cell division, regulation of smooth muscle cell differentiation, positive regulation of mesenchymal cell proliferation, etc., especially nerve cells such as neuroblast division, neuronal stem cell division, and forebrain neuroblast division. KEGG pathway analysis showed that the differential circRNAs were mainly involved in the glycosphingolipid biosynthesis-ganglion series, axon guidance, and longevity-regulating pathway. However, these circRNAs were also detected in human diseases such as gastric cancer, *yersinia* infection, and HIV infection. Further analysis revealed that these circRNAs were also involved in the Wnt signaling pathway and regulation of the actin cytoskeleton. The heat map, volcano map, and signal transduction pathways are shown in Figure 5.

#### 3.2.3 miRNAs sequencing results

**FIGURE 6:**
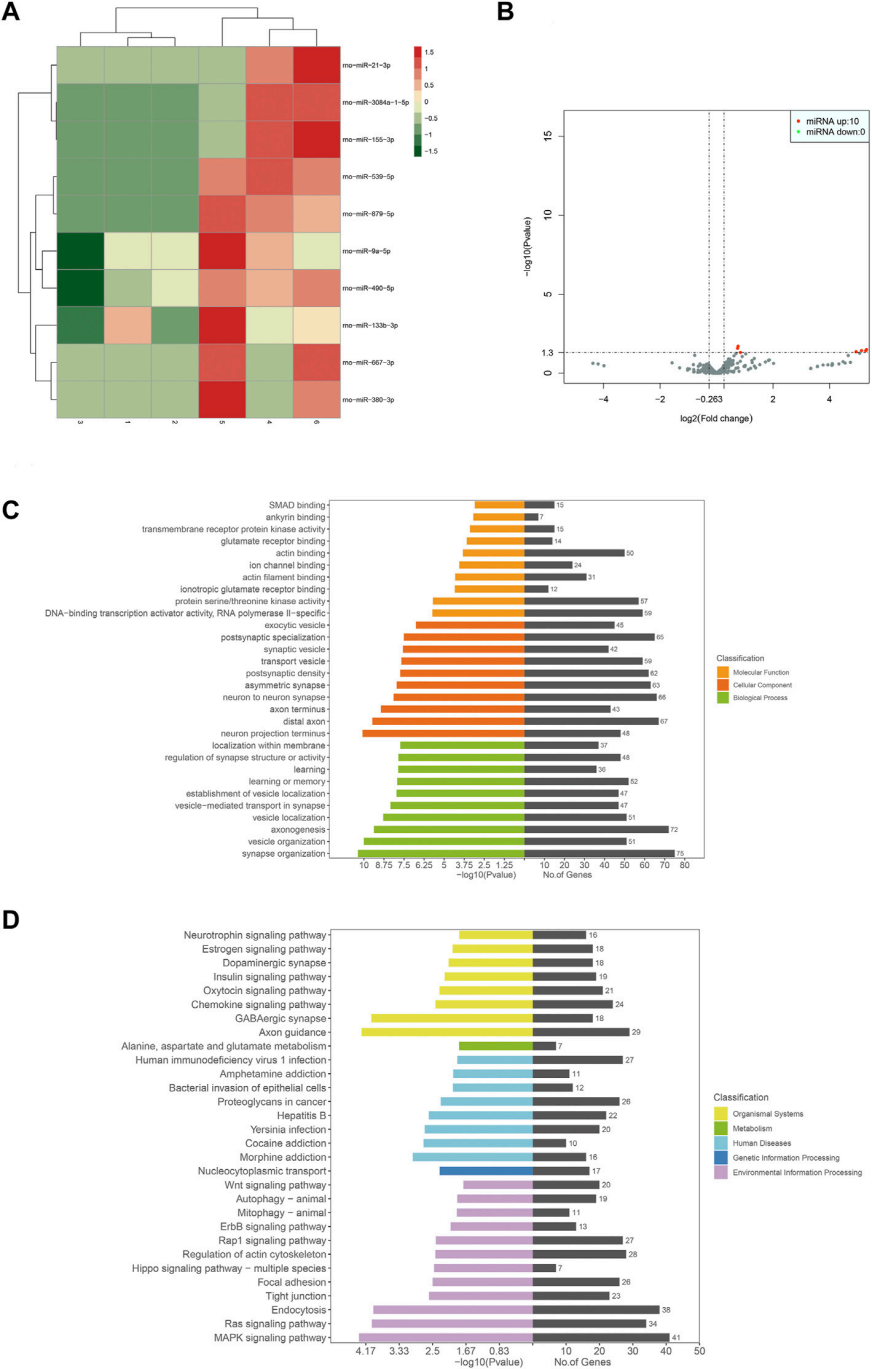
Heat map for analysis of differential expression of miRNAs between samples (control-treatment), color represents log10 (expression value + 1) value **(A)**. The volcano map of miRNAs differential expression analysis (control-treatment) showed that the horizontal coordinate represents the change of miRNAs expression multiple in different samples, the vertical coordinate represents the statistical significance of the difference in miRNAs expression level, and the red dot represents the significant upregulation of miRNAs. Green indicates significantly downregulated miRNA **(B)**. GO enrichment map (control-treatment) **(C)**. **(D)** shows the enrichment pattern of the KEGG pathway of differentially expressed genes (control-treatment).

The sequencing results showed 510 miRNAs. Further analysis revealed only 10 differentially expressed genes. They were miR-155-3p, miR-539-5p, miR-879-5p, miR-21-3p, miR-9a-5p, miR-490-5p, miR-667-3p, miR-3084a-1-5p and miR-380-3p, and all of them were upregulated. GO functional annotation analysis showed that the cellular components were mainly involved in cellular and neuronal synapses, such as postsynaptic specialization, postsynaptic density, asymmetric synapse, neuron-to-neuron synapse, distal axon, etc. The molecular function was mainly involved in the composition of ion channels, receptors, and transcription enzymes, including protein serine/threonine kinase activity, DNA-binding transcription activator activity, and RNA polymerase II-specific. Biological processes were mainly involved in synaptic transmission activities, such as synapse organization, axonogenesis, vesicle organization, etc. KEGG pathway analysis showed that the differential miRNAs were mainly involved in inflammatory responses, such as MAPK signaling pathway, Ras signaling pathway, Wnt signaling pathway, etc. Figure 6 shows the heat map, volcano map, and signal transduction pathways of different miRNAs.

### 3.3 Identification and functional analysis of differential proteins

**FIGURE 7:**
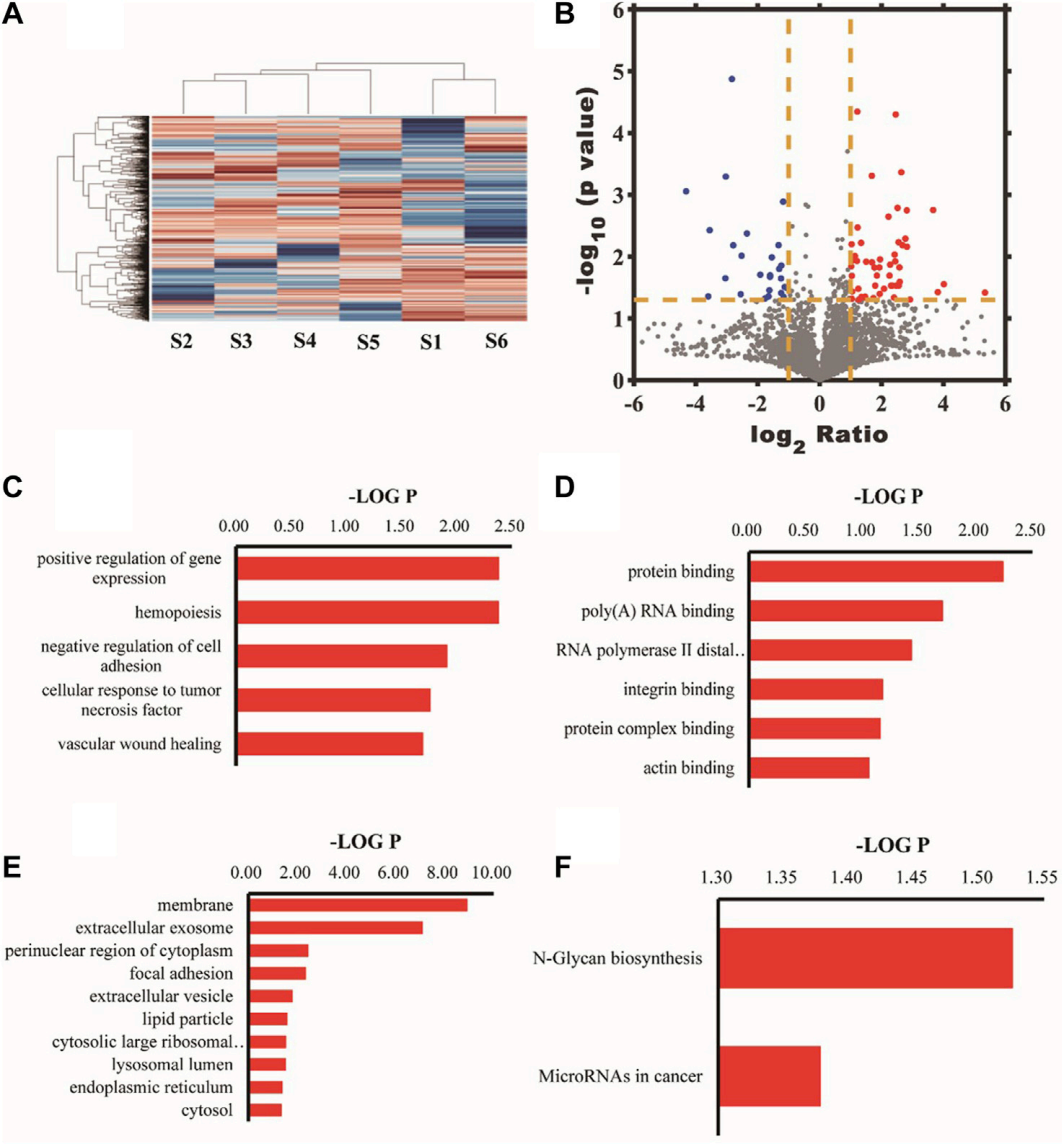
The tree structure on the top represents the similarity settlement relationship between samples, and the tree structure on the left represents the similarity settlement relationship between genes. Red represents increased expression, and blue represents decreased expression **(A)**. The *X*-axis was the ratio of proteins, that was, the ratio of the expression amount in the experimental group samples to the expression amount in the control group samples; the *Y*-axis represented the *p*-value of the repeated test results; each point in the figure represented a protein; the red area was the upregulated protein; the blue area was the downregulated protein **(B)**. Biological processes with the highest differential protein enrichment **(C)**. Molecular functional items with the highest differential protein enrichment **(D)**. Entries of cell composition with the highest differential protein enrichment **(E)**. Analysis of differential protein KEGG metabolic pathway **(F)**.

A total of 4049 proteins were identified. Quantitative information on 3670 proteins was obtained after further processing, among which 78 were differential proteins. Differential protein GO analysis showed that the top 10 cell composition items (CCs) with the highest enrichment were membrane, extracellular exosome, perinuclear region of cytoplasm, focal adhesion, extracellular vesicle, lipid particle, cytosolic large ribosomal subunit, lysosomal lumen, endoplasmic reticulum, and cytosol respectively. The most enriched molecular function items (MFs) were protein binding, poly(A)RNA binding, RNA polymerase II distal enhancer sequence-specific DNA binding, integrin binding, complex protein binding, and actin binding. The most enriched biological process items (BPs) were positive regulation of gene expression, hemopoiesis, negative regulation of cell adhesion, cellular response to tumor necrosis factor, vascular wound healing, S-adenosylhomocysteine metabolic process, regulation of protein metabolic process, retrograde axonal transport, positive regulation of protein targeting to the mitochondrion, and surfactant homeostasis. Analyzing metabolic pathways enriched by differential proteins, we found that the N-Glycan biosynthesis pathway was mainly involved. Figure 7 shows the heat map, volcano map, and signal transduction pathways of differential proteins.

## 4 Discussion

In this study, we investigated the whole-transcriptomic and proteomic sequencing information from P-ALI and identified potential therapeutic targets that may be useful for exploring neuronal inflammatory responses. Acute lung injury caused by phosgene inhalation is severe and is considered lethal. Furthermore, P-ALI has a rapid disease progression, a poor prognosis, and a high mortality and disability rate, all of which endanger people’s health and safety. Currently, most studies focus on treating P-ALI, such as oxygen therapy, hormone therapy, symptomatic supportive therapy, etc., while there are few studies on the key factors affecting the prognosis and the potential mechanisms related to prognosis. Therefore, a new method is needed to reveal the key prognostic factors of P-ALI and screen the genes related to prognosis to treat P-ALI more effectively. The popularity and development of gene sequencing technology make people through a key gene for gene sequencing revealed many disease outcomes. The advent of high-throughput sequencing technology allows people to rediscover noncoding RNAs (ncRNAs), resolving many previously undiagnosed diseases and altering people’s understanding of the pathophysiology of many diseases. It suggests that precision medicine has a broad application prospect in the future.

By replicating the rat model of P-ALI, we compared the general state changes of rats in the P-ALI group and air group, and we constructed a sequencing database, combined with the biological information analysis method, to explore the key genes affecting the progression of P-ALI.

The data showed that the wet/dry ratio of the lung, neutrophil count, and protein content in BALF rats of the P-ALI group were significantly increased (*p* < 0.05). Similarly, the contents of IL-1β, IL-18, and IL-33 in serum and BALF were significantly increased compared with those in the air group (*p* < 0.05), which was consistent with the findings of our previous research and other scholars (He et al., 2018; Jin et al., 2020). Lung tissue typically contained the richest types of immune cells and played a vital role in the immune response to overproduction of inflammatory cytokines (Cao et al., 2023), so the interaction between inflammatory factors and proteins in BALF revealed the severity of phosgene inhalation in lung tissue. Compared with the existing literature on lung injury models, many studies have focused on bronchial epithelial cell death or alveolar epithelial barrier breakdown caused by elevated levels of inflammatory factors as important mechanisms of P-ALI. (Lu et al., 2021; He et al., 2023), while our study focused on the presence of neuronal inflammatory signaling pathways.

Through high-throughput gene sequencing screening, we found 225 differentially expressed long noncoding RNAs (lncRNAs) between the two groups, including 85 upregulated genes and 140 downregulated genes. In addition, there were 42 differential circRNAs, including 25 upregulated genes and 17 downregulated genes. Only 10 differentially expressed miRNAs genes were upregulated. In this study, lncRNAs were the genomes with the largest changes in transcriptome gene expression.

Usually, lncRNAs were more than 200 nt in length, localized in the nucleus or cytoplasm, and regulated various biological processes, including epigenetic, transcriptional, and post-transcriptional levels, such as chromatin modification, transcriptional activation, and inhibition. As inducer molecules of miRNAs interfere with gene expression (Batista and Chang, 2013; Kopp and Mendell, 2018), circRNAs are widely found in eukaryotes and are usually produced by splicing cyclization of mRNA precursors (pre-mRNA). They were formed by reverse splicing between the “head” of the previous exon and the “tail” of the last exon (Patop et al., 2019). circRNAs were involved in various biological processes, especially in the development of animals and plants and the occurrence and development of diseases (Chen et al., 2021). MicroRNAs, the significant components of small RNAs, were multifunctional regulators of gene expression in higher eukaryotes. It has been proven closely related to various diseases, including cancer, cardiovascular diseases, immune system diseases, allergic diseases, etc. (Iorio and Croce, 2012; Lu and Rothenberg, 2018; Van Meter et al., 2020). As indispensable regulators in life activities, miRNAs were involved in critical biological processes such as gene expression and regulation, RNA processing and cleavage, protein translation, genetic “invasion” inhibition, and gametogenesis (Mohr and Mott, 2015). It was an emerging biomarker for early cancer diagnosis and prognosis (Mohr and Mott, 2015). By comparing the GO function analysis through the network database, we found that differential lncRNAs and miRNAs were mainly involved in the cytoplasm, neurotransmitter transmission, ion signaling pathway conduction, synaptic vesicles, and neuronal projection. In contrast, differential circRNAs were mainly involved in cell composition, growth, differentiation, and division. KEGG enrichment analysis showed that neuronal projection activity and inflammatory pathways were dominant, and lncRNAs were mainly neural ligand-receptor interaction pathways; circRNAs were mainly involved in growth and development pathways, and some of these were involved in inflammatory pathways such as the Wnt signaling pathway. The miRNAs were mainly inflammatory pathways, and the detected signaling pathways included the MAPK signaling pathway, Ras signaling pathway, Wnt signaling pathway, chemokine signaling pathway, and neutrophil signaling pathway. Therefore, it can be concluded that genes and pathways related to neuronal projection and inflammation regulation determine the severity and prognosis of P-ALI. A systematic review showed that the pathophysiological mechanisms of phosgene inhalation lung injury included lung epithelial-endothelial cell injury, immune microenvironment changes, and pulmonary neuronal stimulation (Cao et al., 2022). Neuronal pulmonary edema was considered to be a key pathogenic factor of P-ALI. Our study confirmed the presence of neuronal projections in P-ALI response. In this pathophysiological process, reducing hypoxia may play a protective role in P-ALI (Zhang et al., 2023). However, the specific mechanism needs further study.

More and more studies suggested that ncRNAs are related to many cellular functions and have become a hot topic in protein interaction (Ferre et al., 2016; Zang et al., 2020). Differential protein identification and functional analysis were carried out by Label-free technology. The results showed that the cell components with the highest differential protein enrichment were cell entries, adhesion spots, extracellular vesicles, molecular function gene transcription, integrin binding, complex protein binding, and actin junction. Biological processes involve growth and development, regulation of inflammatory factors, protein metabolism, etc. The differential protein KEGG metabolic pathway analysis was mainly based on N-Glycan biosynthesis.

N-Glycan biosynthesis was essential for both single-celled and multicellular organisms. It regulated various biological functions involving protein folding, transport, classification, localization, signaling, proliferation, migration, and environmental adhesion (Li et al., 2021; Viinikangas et al., 2021). In addition, it plays a vital role in the immune system, pathogen recognition, and cancer (Bhide and Colley, 2017). However, its relationship and mechanism with ncRNAs must be confirmed further.

In this study, we showed the characteristics of P-ALI analyzed based on transcriptomics and proteomics systematically, which highlighted novel ideas for the treatment of it. With the existing research, the therapeutic direction of P-ALI was focused on improving inflammatory response, regulating immune microenvironment and repairing damaged tissues. Therefore, the neuronal pathway of lung tissue provided a very promising therapeutic prospect for improving the targeted therapy of P-ALI. Future preclinical and clinical trials were critical to evaluate and optimize therapies to best address the challenges that exist in the treatment of P-ALI.

## 5 Conclusion

Through genomic analysis and bioinformatics analysis by high-throughput sequencing technology, we found that neuronal injury in lung tissue may be a key factor affecting the prognosis of acute lung injury caused by phosgene, which leads to changes in inflammation and metabolic levels. However, more basic experimental studies should be required to investigate the specific mechanism.

## 6 Innovations and limitations

Innovations: We analyzed transcriptome genes and proteomes using high-throughput technology combined with proteome identification for P-ALI with a poor prognosis. GO and KEGG results can provide many new directions for future research.

Limitations: On the one hand, there were some potential biases and limitations in our study design, sample size, and analytical methods, sunch as the lack of comparative analysis of the human genome, the small sample size for animals and the poor statistical methodsand. On the other hand, although NGS was used to screen a large number of differential genes. However, additional validation via qPCR, Western blot, and other technologies were required to ensure the accuracy of screening differential genes.

## Data Availability

The original contributions presented in the study are included in the article/Supplementary Material, further inquiries can be directed to the corresponding author.
