# Supplementary figures and images for: Characteristics of phosgene aspiration lung injury analyzed based on transcriptomics and proteomics
Source: Front Genet. 2024 May 17;15:1393665. doi: 10.3389/fgene.2024.1393665 (PMC11140124; doi:10.3389/fgene.2024.1393665)

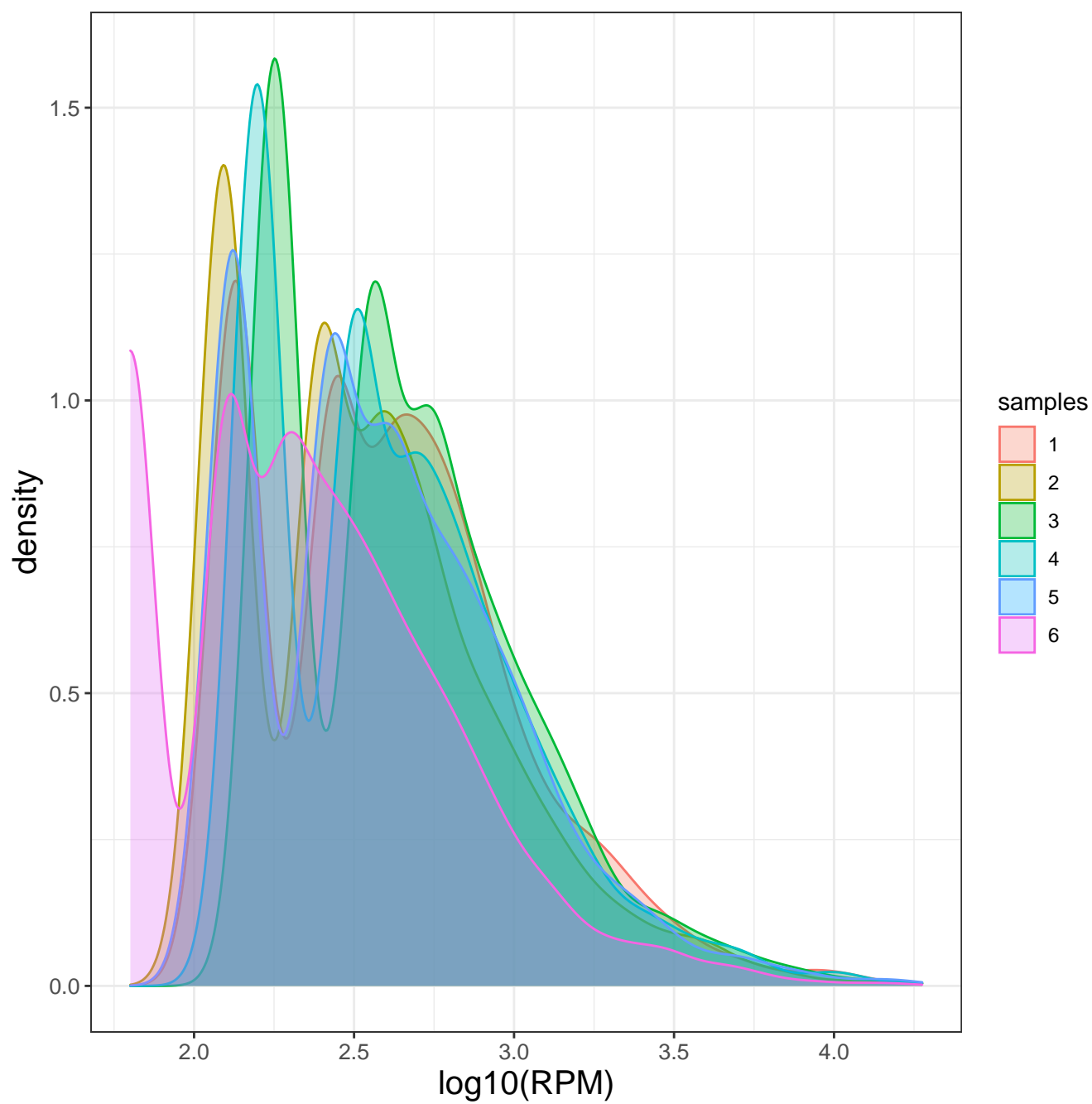

Supplement: Supplementary file 1 [file Presentation1.zip › Data/CircRNA/circ_expression/sample_density.pdf]

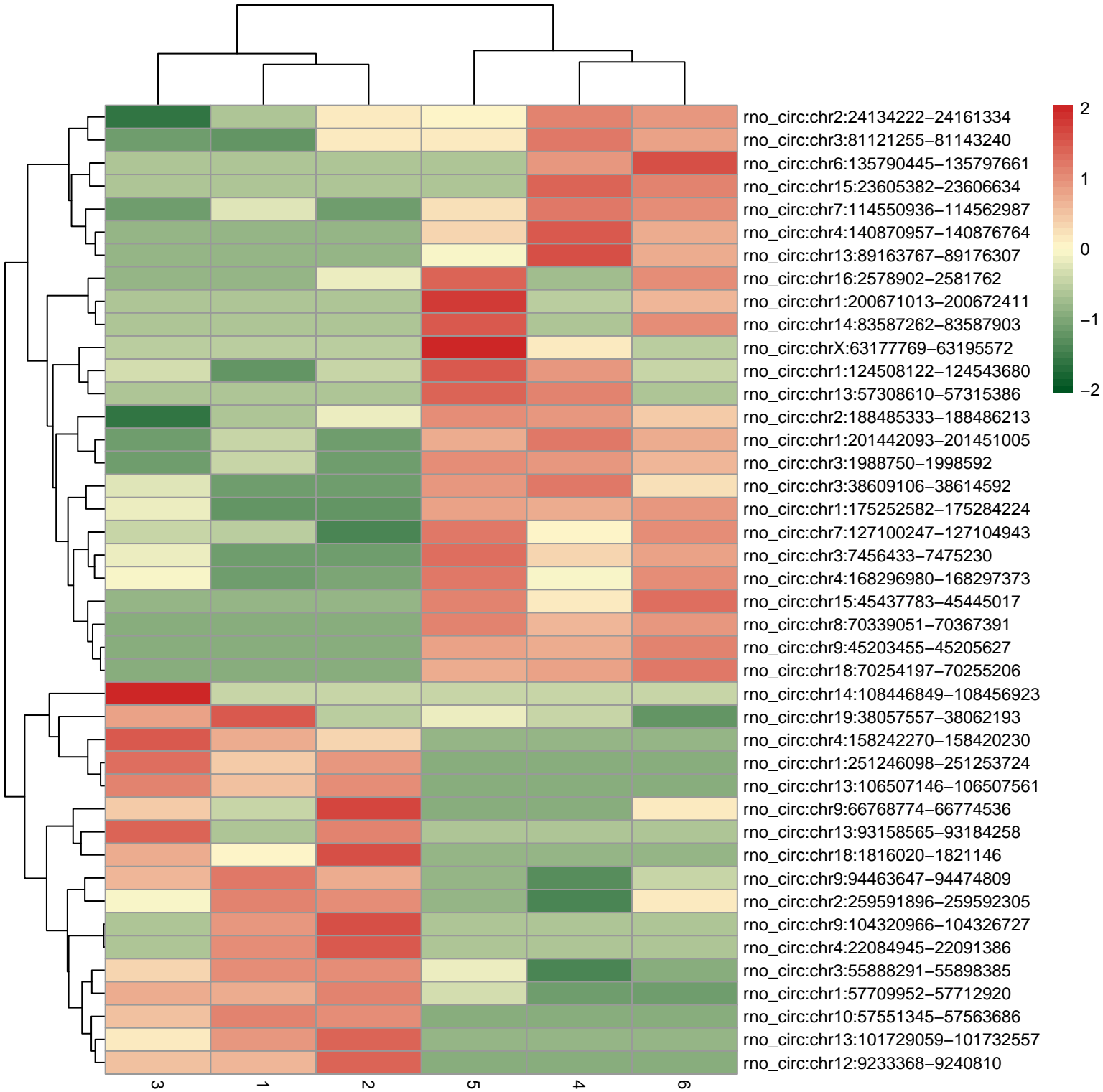

Supplement: Supplementary file 1 [file Presentation1.zip › Data/CircRNA/differential_expression/Control--Treatment/Control--Treatment.circRNA.heatmap.pdf]

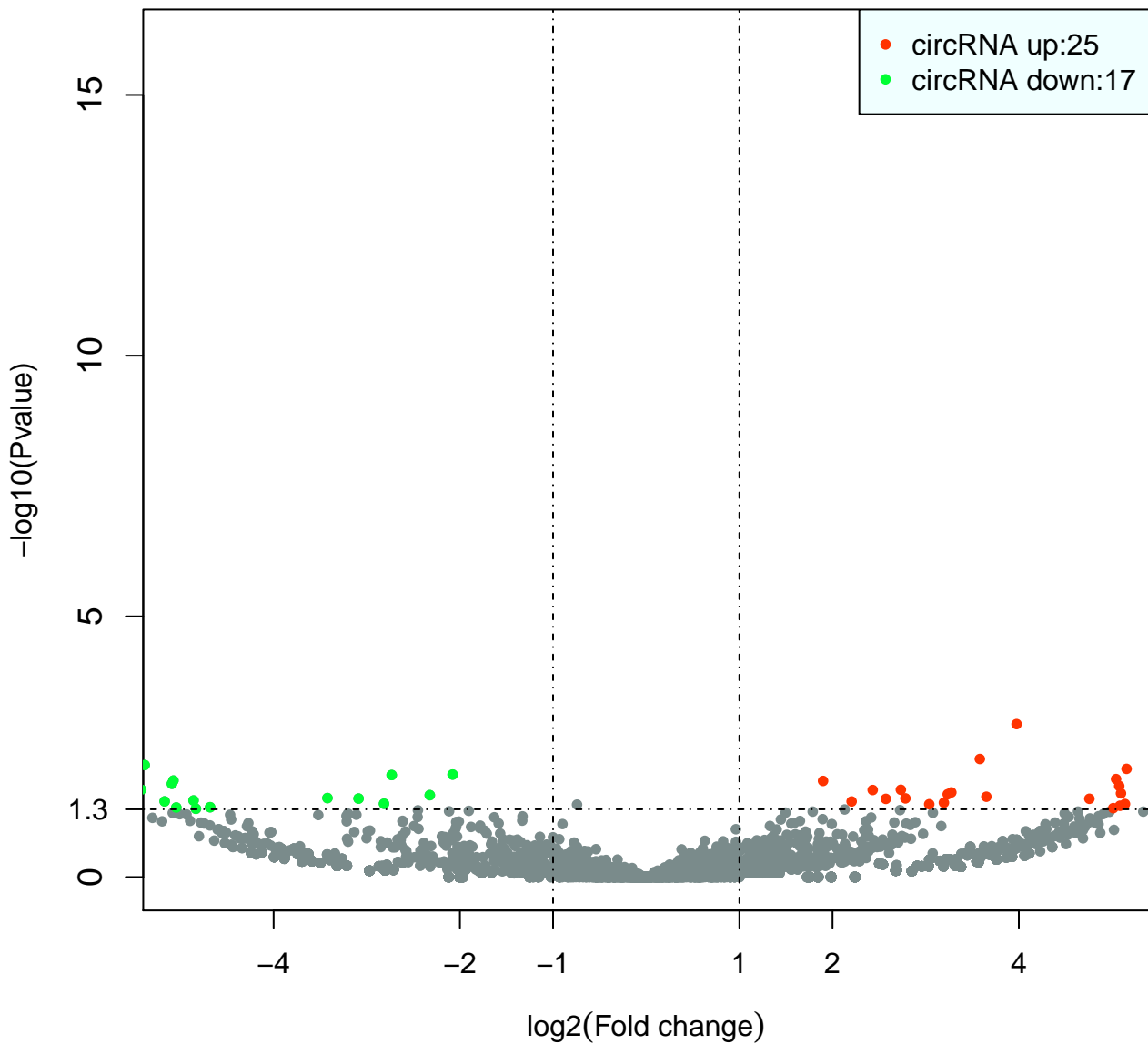

Supplement: Supplementary file 1 [file Presentation1.zip › Data/CircRNA/differential_expression/Control--Treatment/Control--Treatment.circRNA.volcano.pdf]

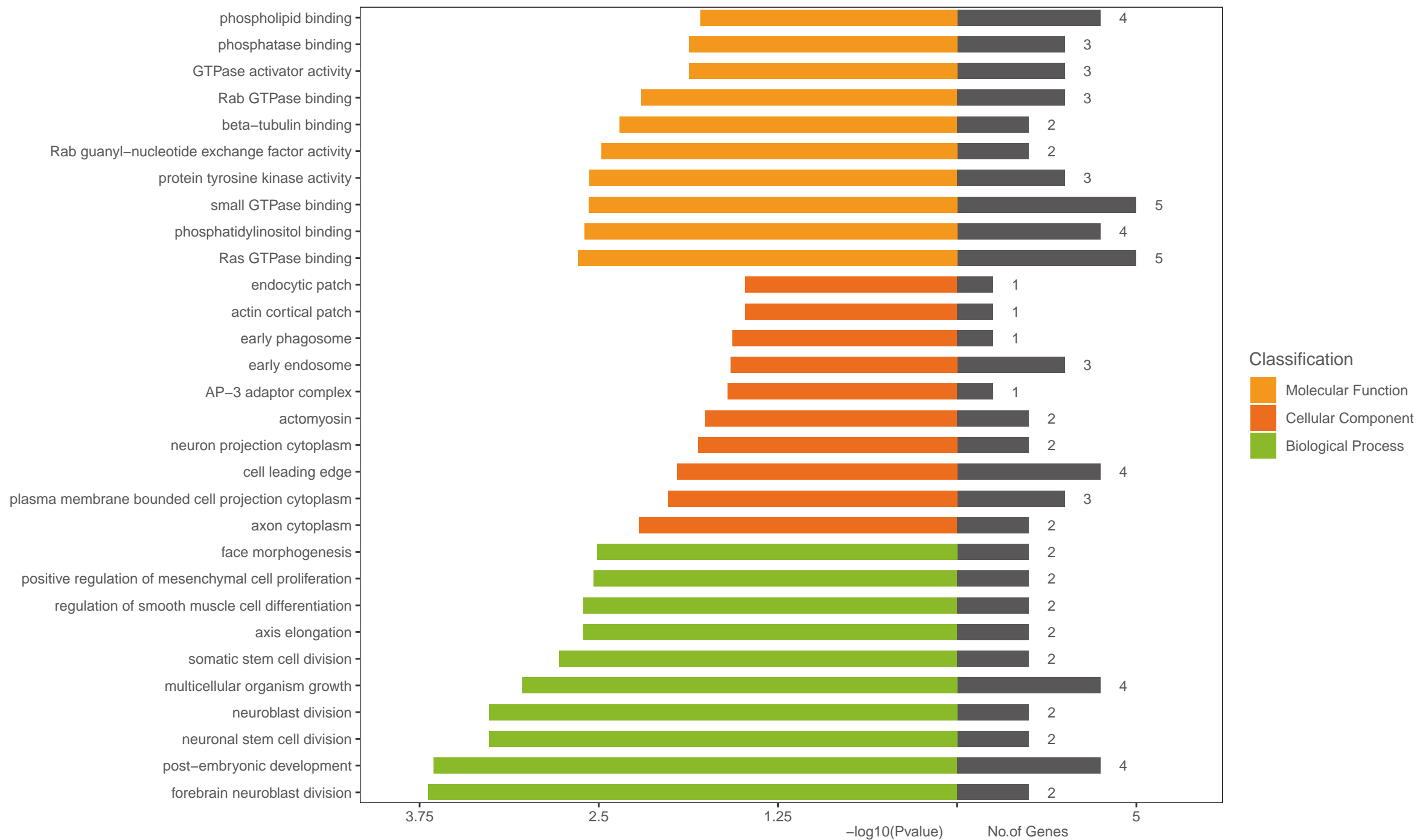

Supplement: Supplementary file 1 [file Presentation1.zip › Data/CircRNA/GO/Control--Treatment/go.pdf]

Statistics of Pathway Enrichment

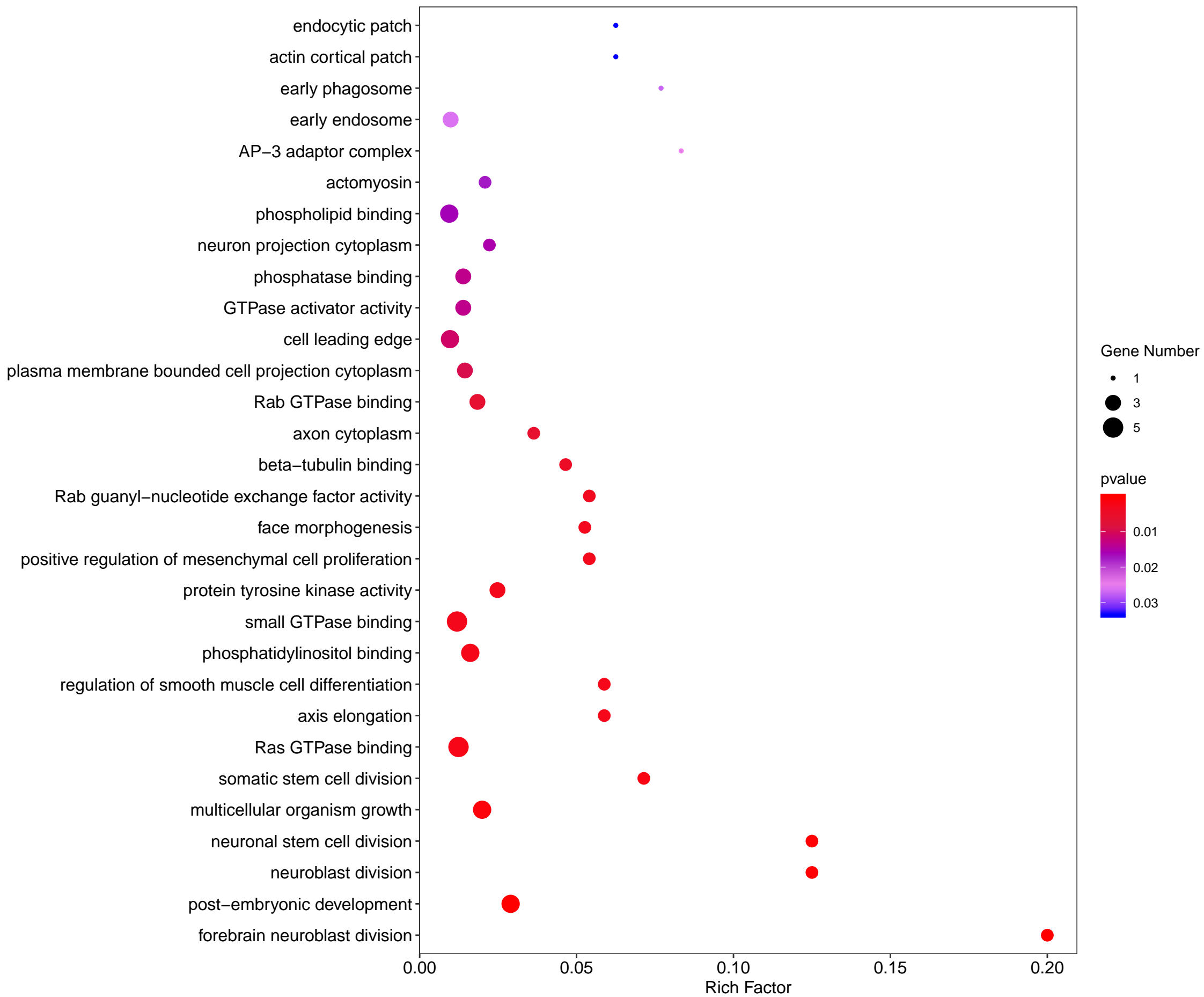

Supplement: Supplementary file 1 [file Presentation1.zip › Data/CircRNA/GO/Control--Treatment/go.point.pdf]

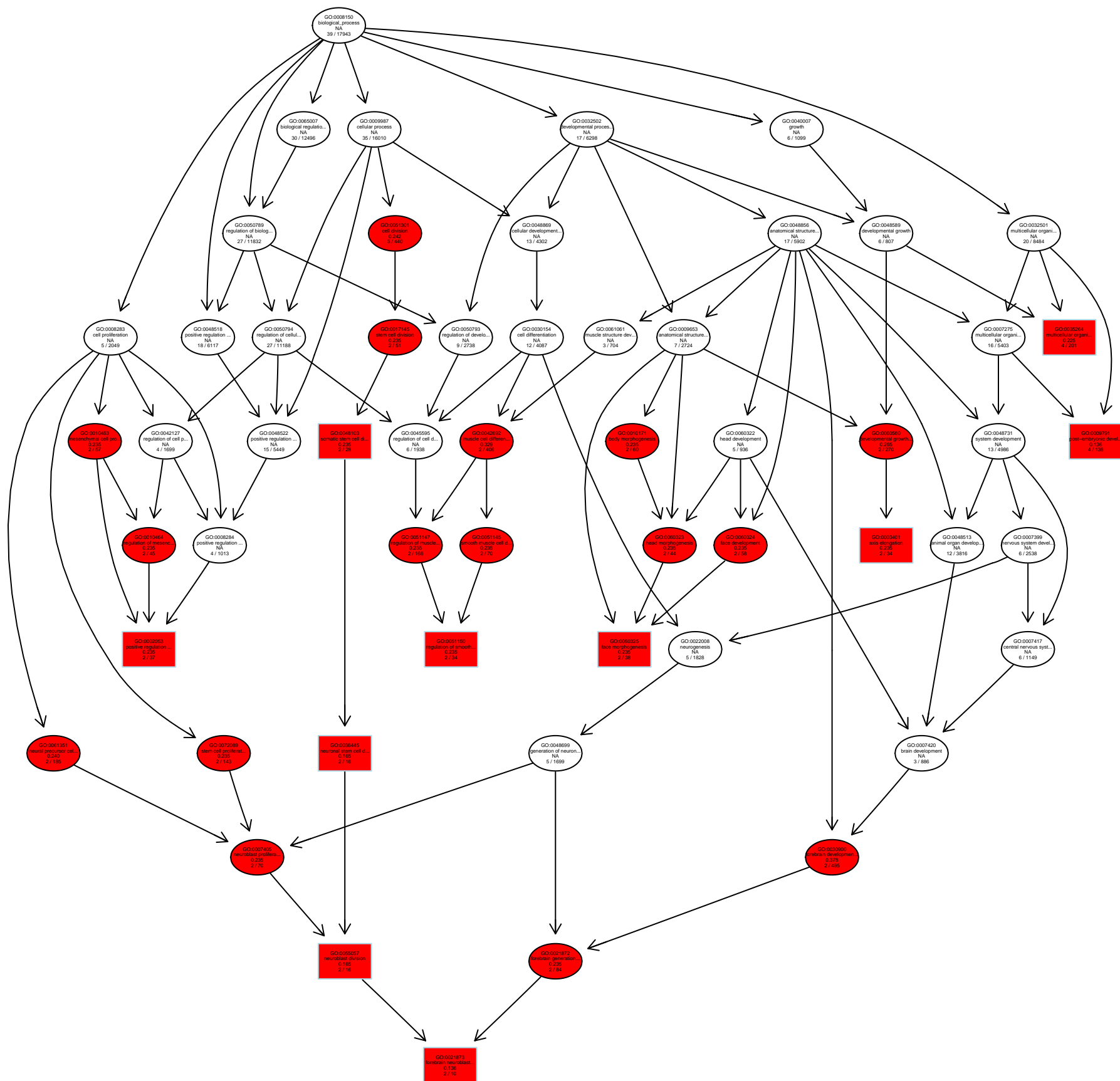

Supplement: Supplementary file 1 [file Presentation1.zip › Data/CircRNA/GO/Control--Treatment/GO-Biological_Process.pdf]

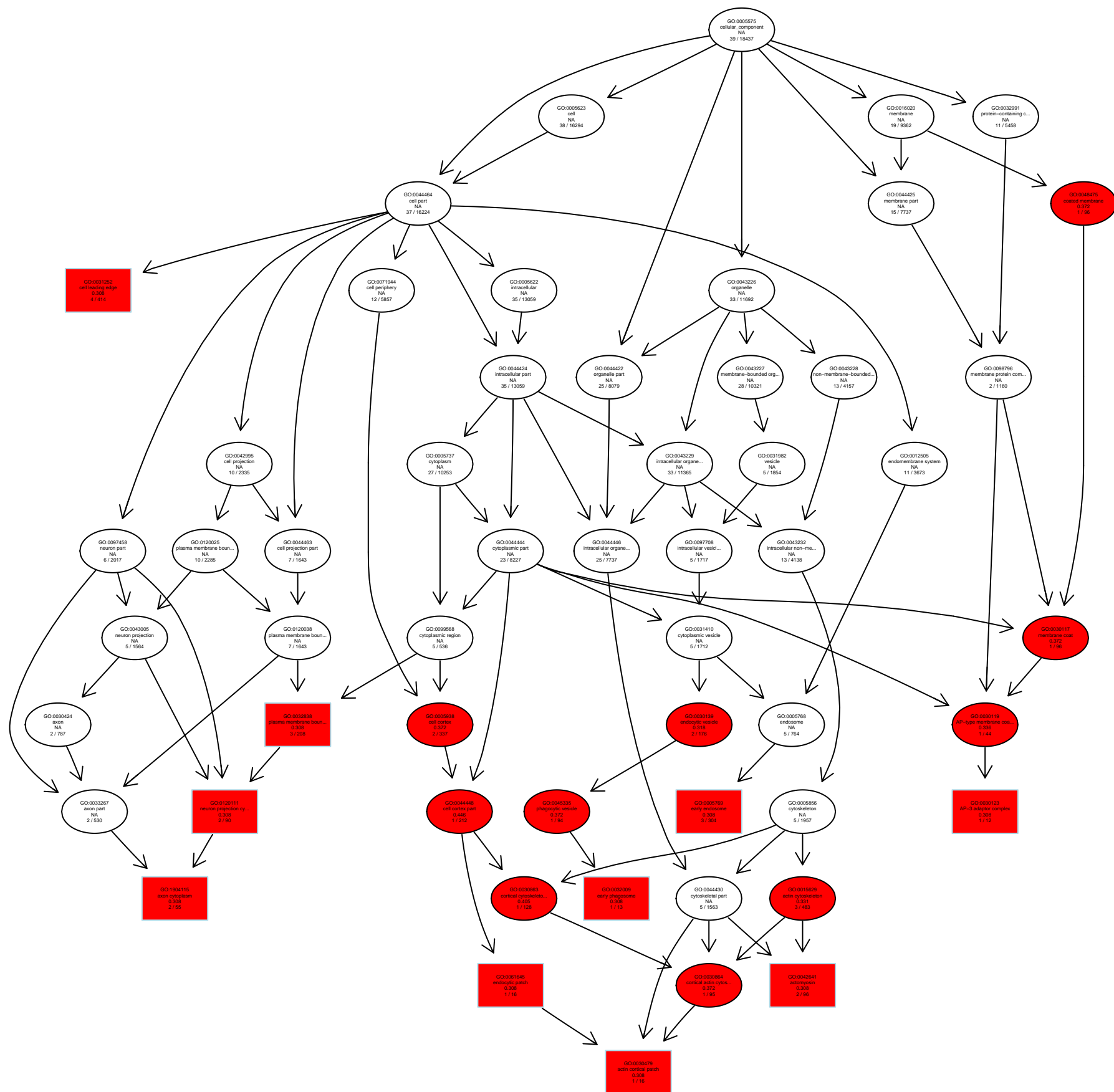

Supplement: Supplementary file 1 [file Presentation1.zip › Data/CircRNA/GO/Control--Treatment/GO-Cellular_Component.pdf]

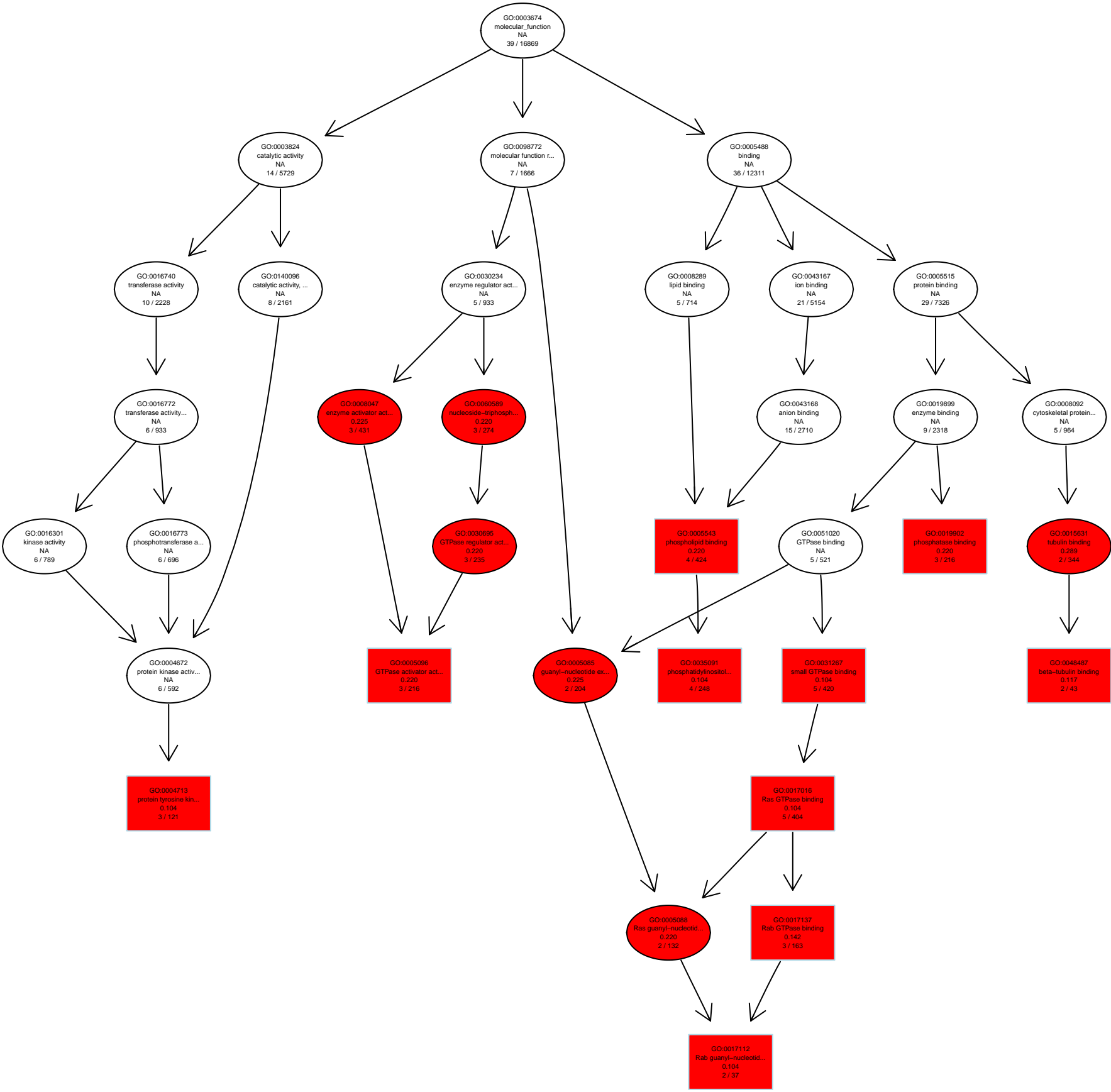

Supplement: Supplementary file 1 [file Presentation1.zip › Data/CircRNA/GO/Control--Treatment/GO-Molecular_Function.pdf]

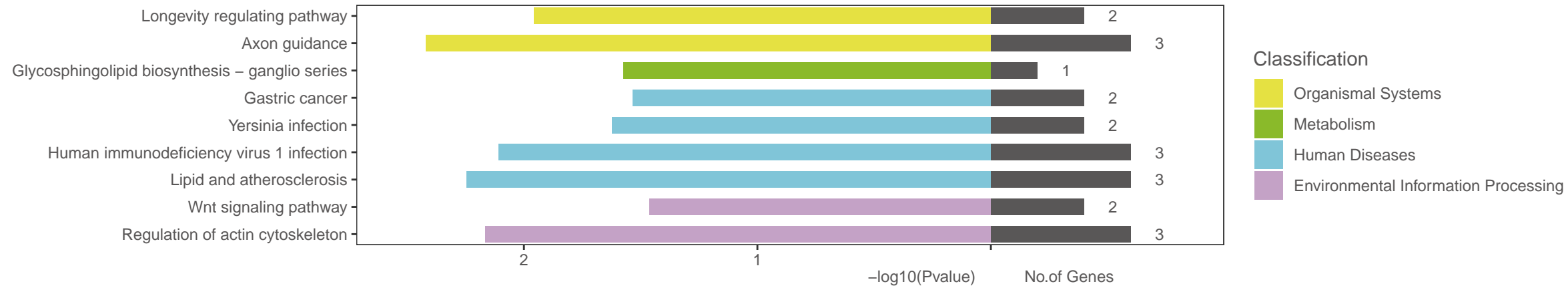

Supplement: Supplementary file 1 [file Presentation1.zip › Data/CircRNA/KEGG/Control--Treatment/kegg.pdf]

Statistics of Pathway Enrichment

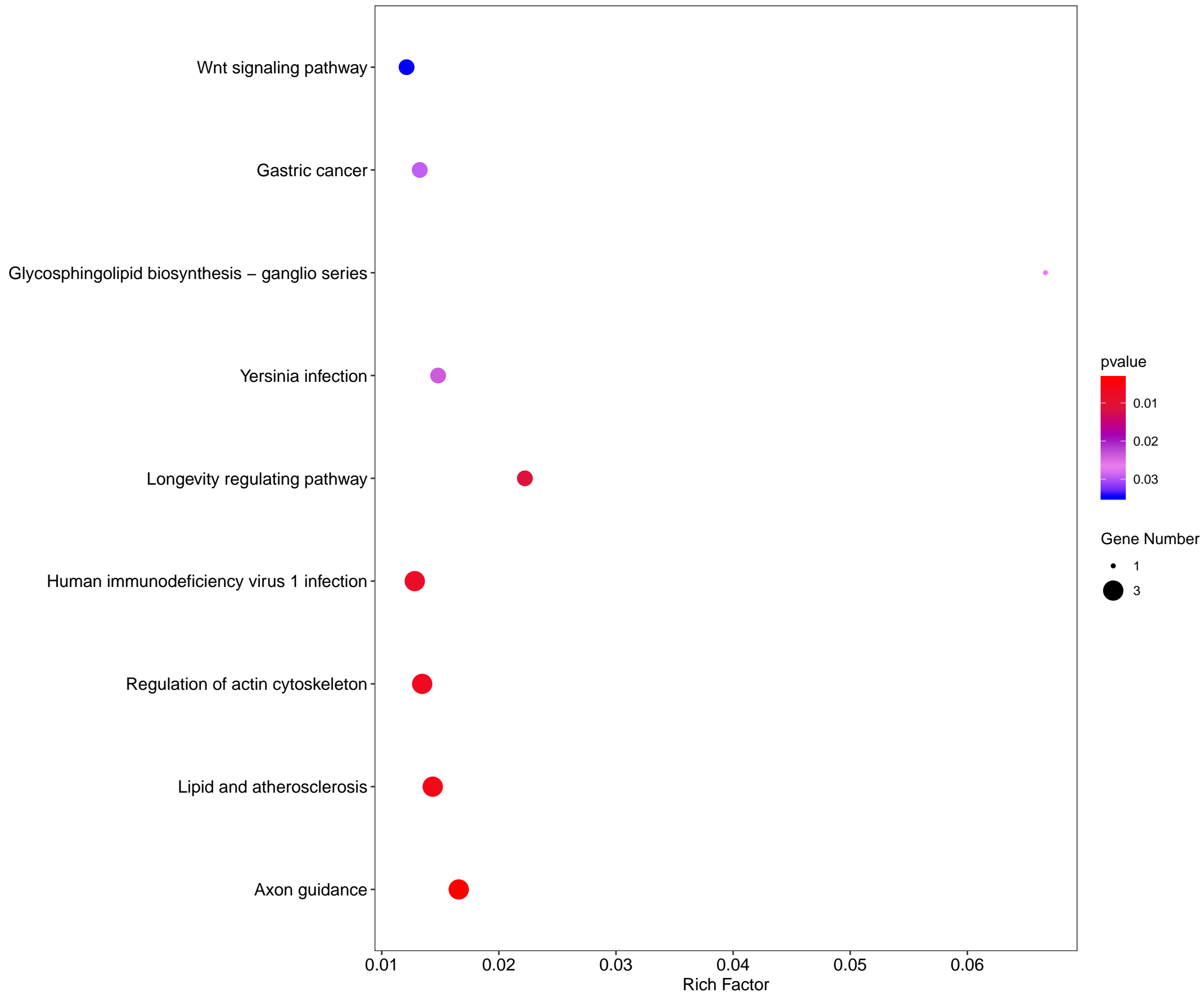

Supplement: Supplementary file 1 [file Presentation1.zip › Data/CircRNA/KEGG/Control--Treatment/kegg.point.pdf]

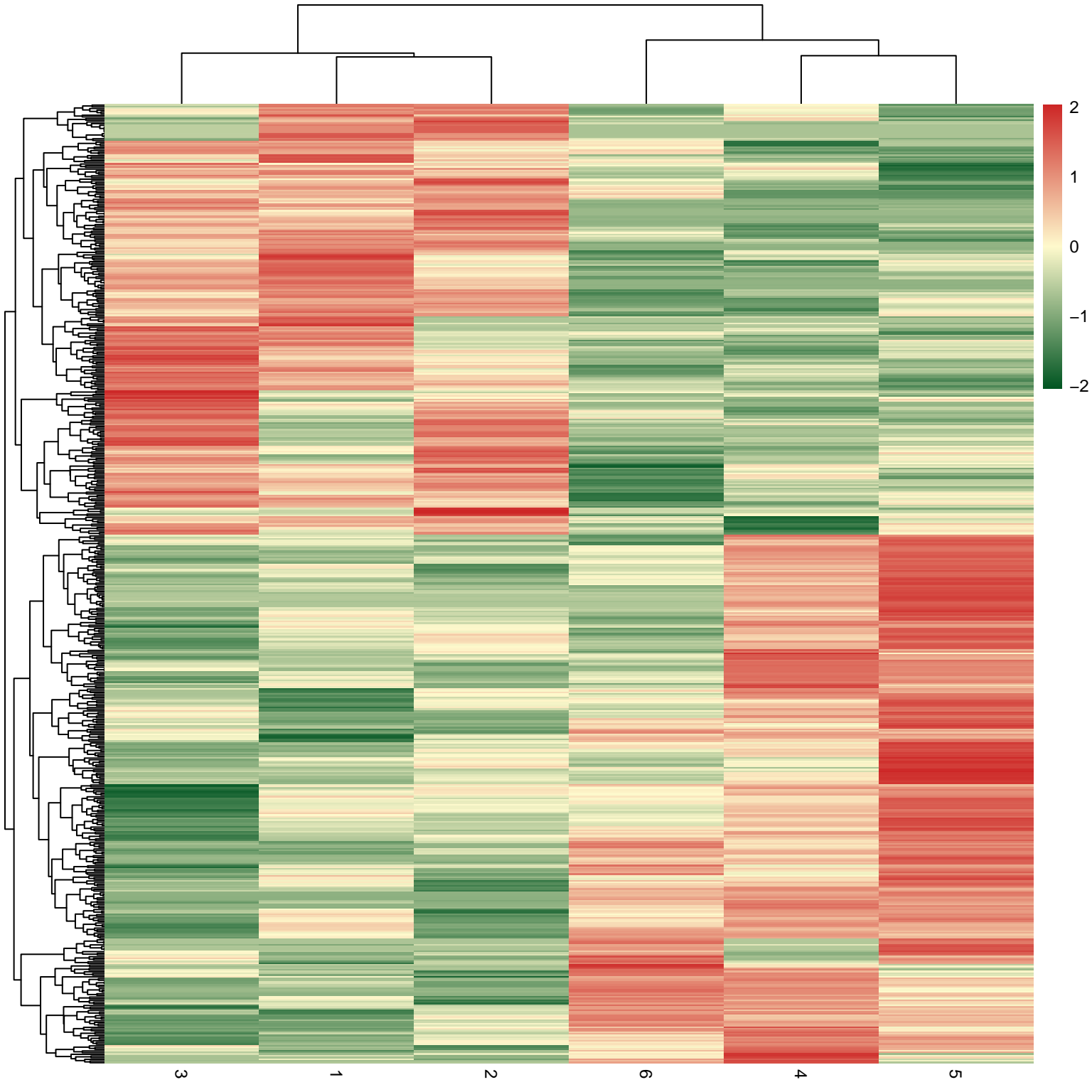

Supplement: Supplementary file 1 [file Presentation1.zip › Data/lncRNA/Gene_differential_expression/Control--Treatment/Control--Treatment.heatmap.gene.pdf]

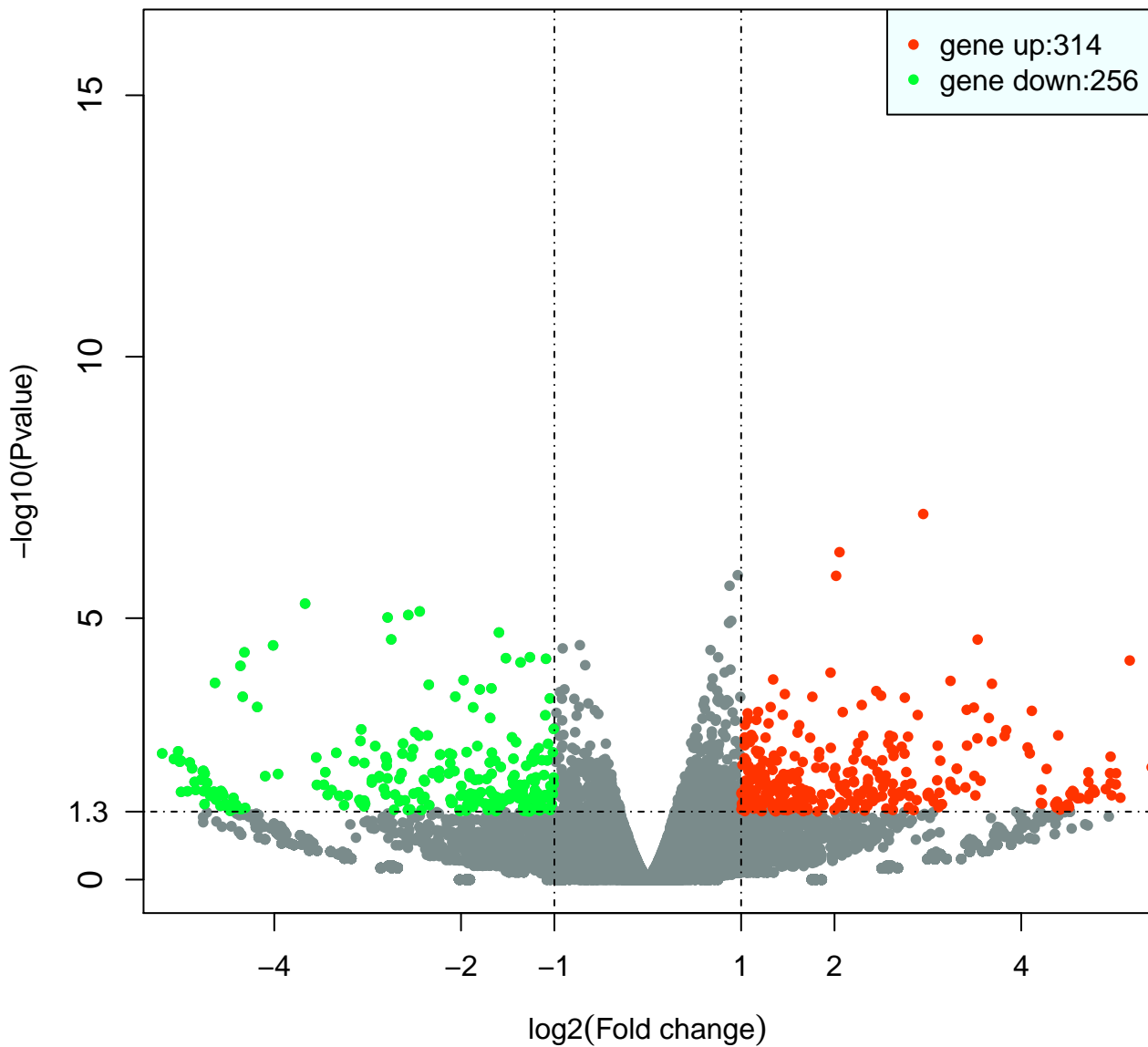

Supplement: Supplementary file 1 [file Presentation1.zip › Data/lncRNA/Gene_differential_expression/Control--Treatment/Control--Treatment.volcano.gene.pdf]

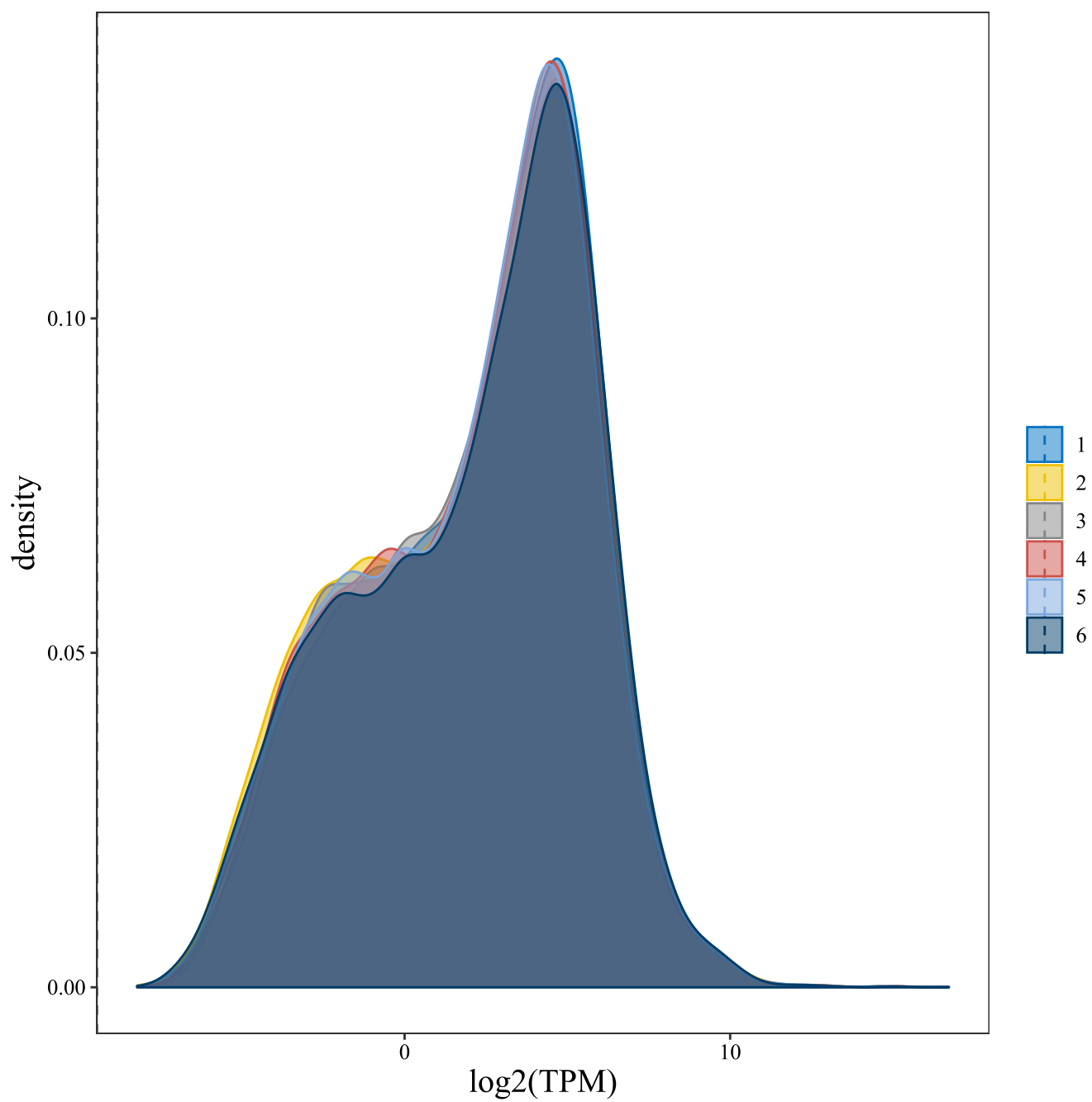

Supplement: Supplementary file 1 [file Presentation1.zip › Data/lncRNA/Gene_expression/gene_density.pdf]

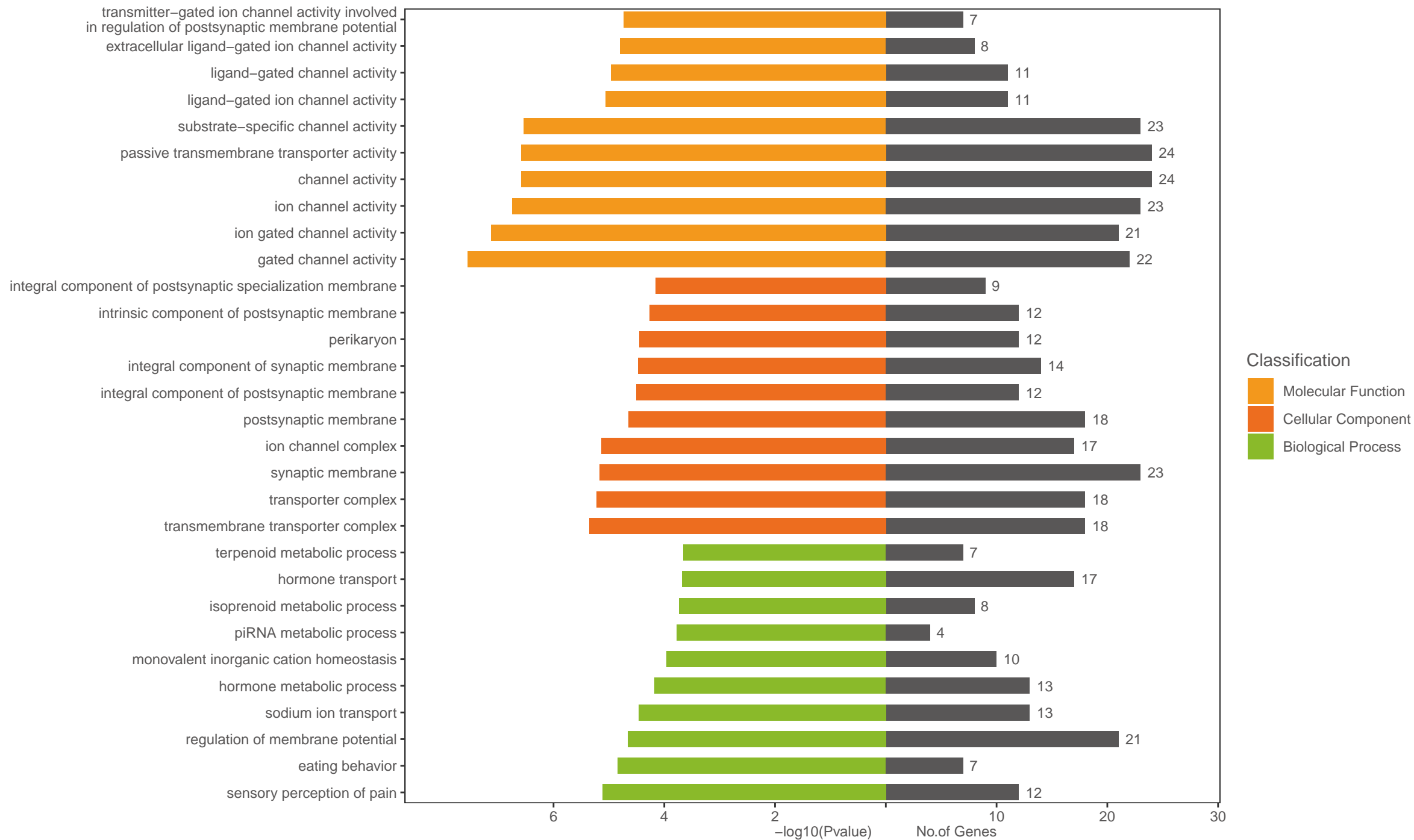

Supplement: Supplementary file 1 [file Presentation1.zip › Data/lncRNA/GO/Control--Treatment/go.pdf]

Statistics of Pathway Enrichment

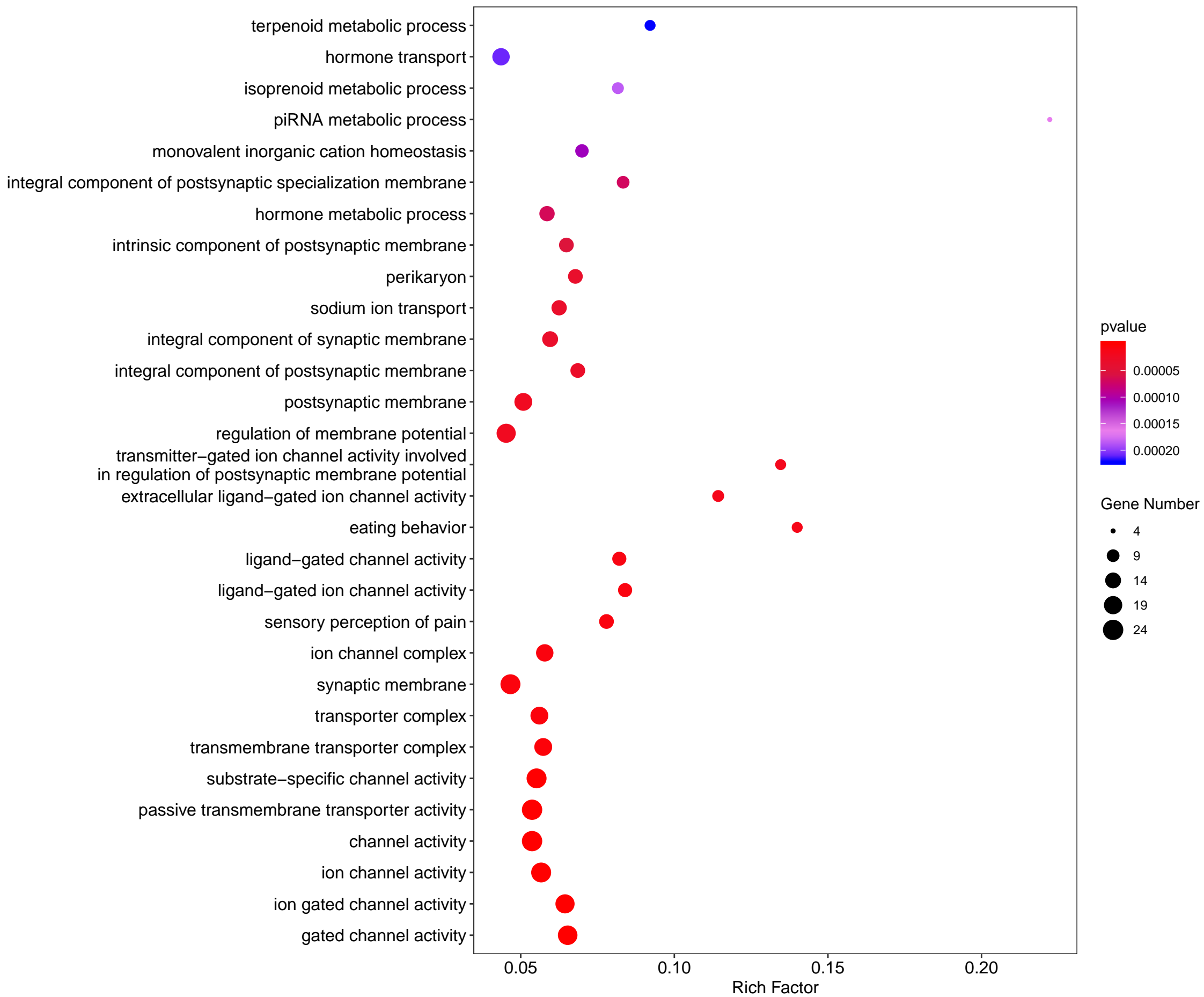

Supplement: Supplementary file 1 [file Presentation1.zip › Data/lncRNA/GO/Control--Treatment/go.point.pdf]

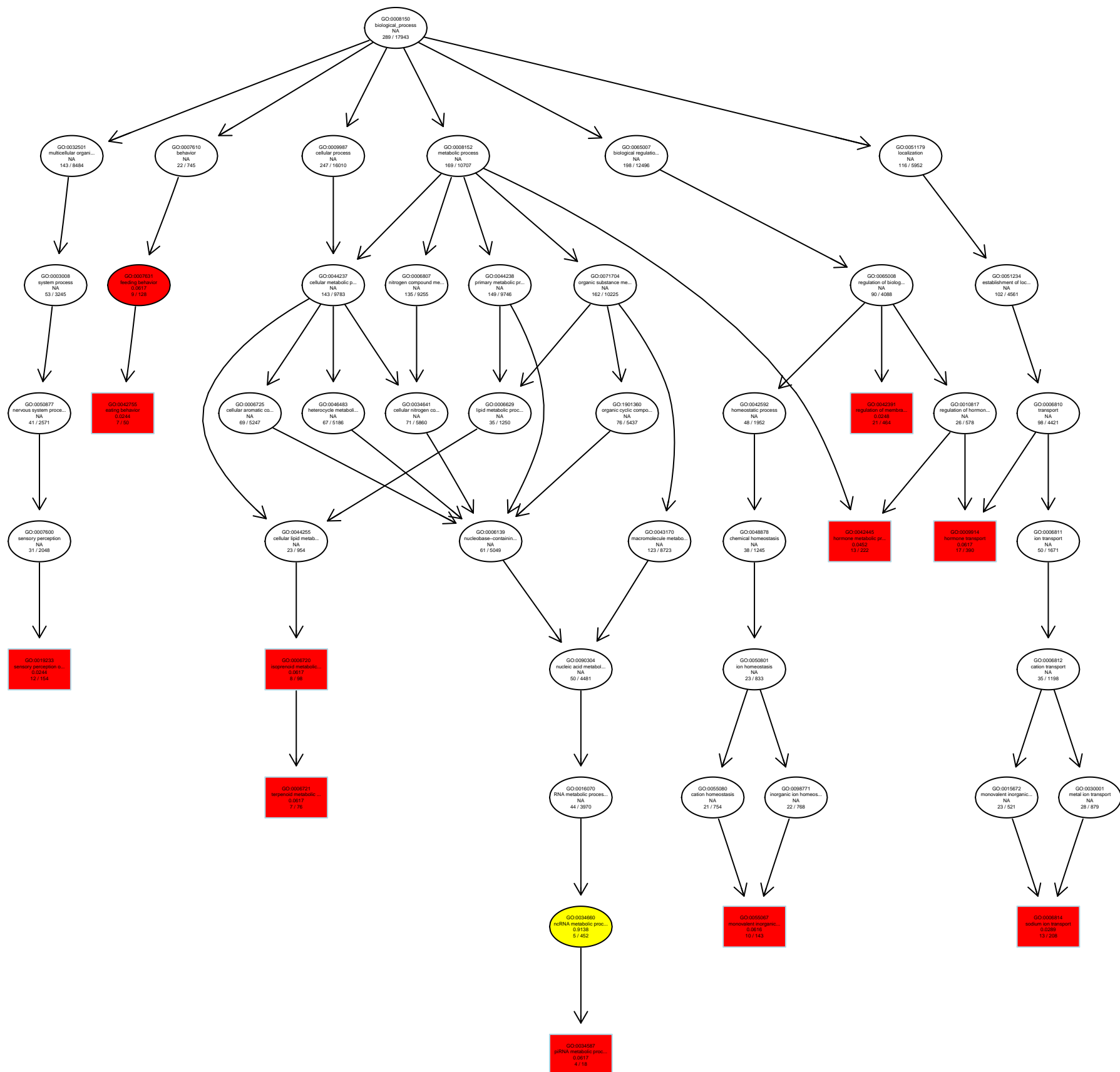

Supplement: Supplementary file 1 [file Presentation1.zip › Data/lncRNA/GO/Control--Treatment/GO-Biological_Process.pdf]

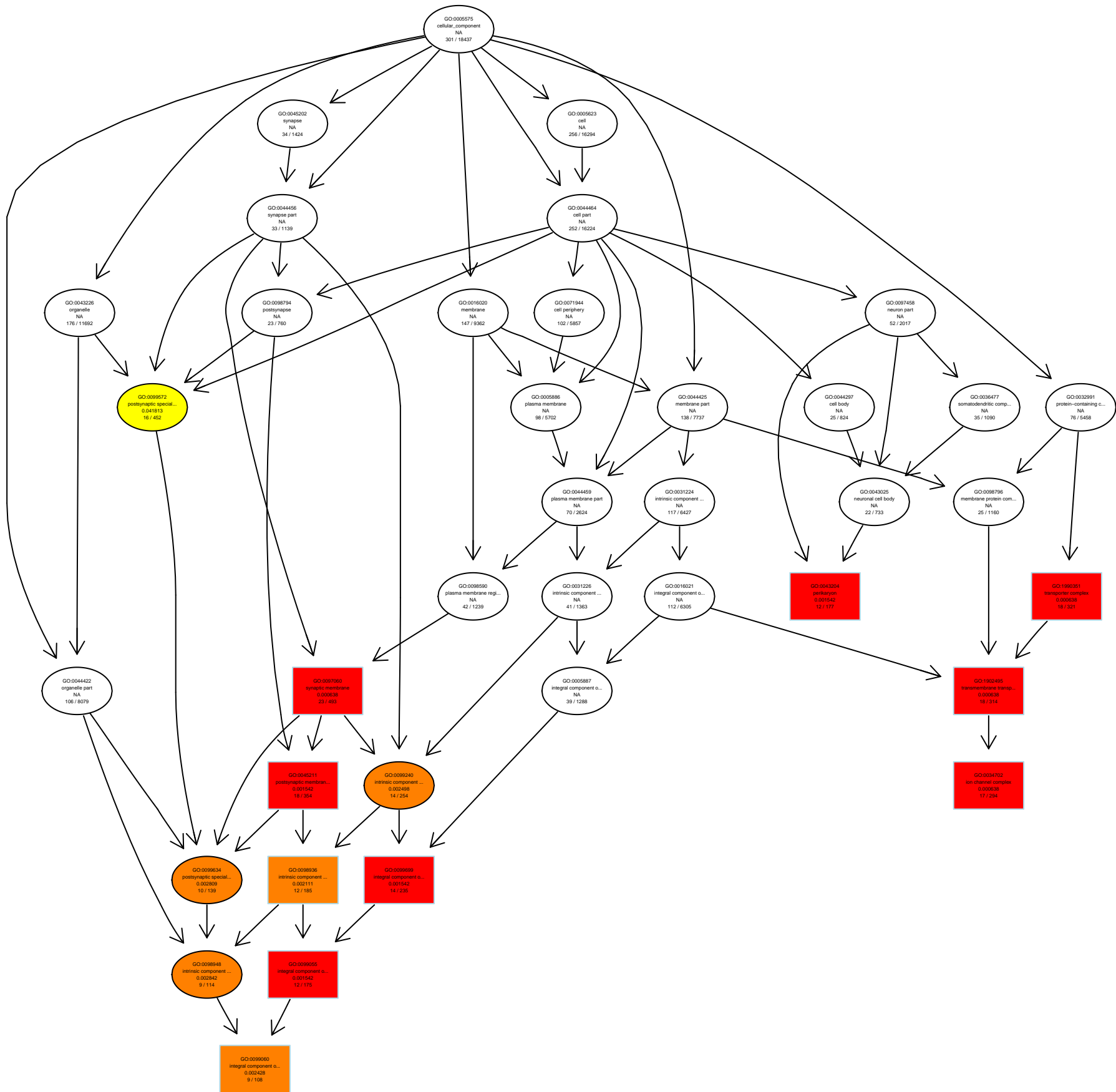

Supplement: Supplementary file 1 [file Presentation1.zip › Data/lncRNA/GO/Control--Treatment/GO-Cellular_Component.pdf]

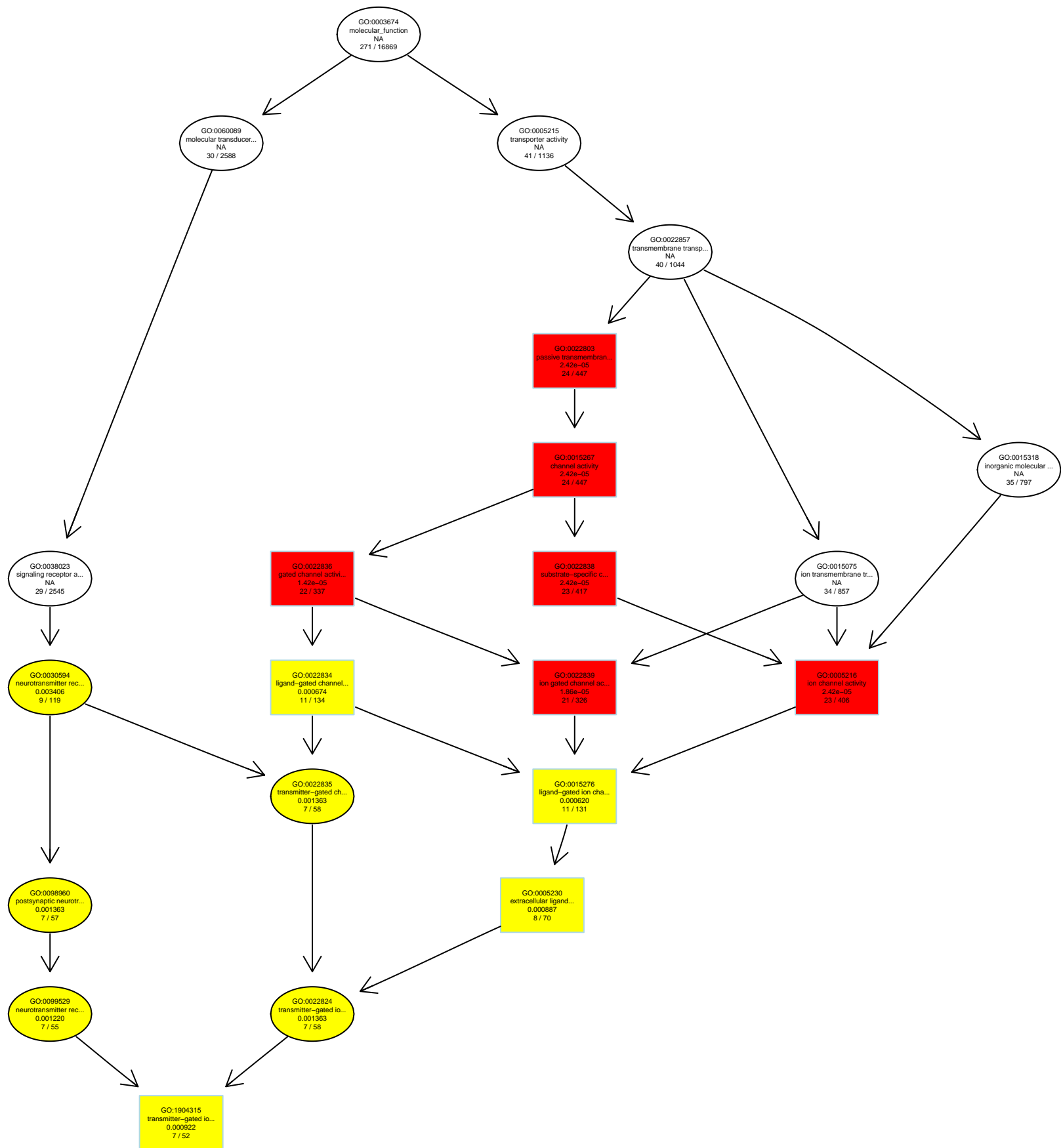

Supplement: Supplementary file 1 [file Presentation1.zip › Data/lncRNA/GO/Control--Treatment/GO-Molecular_Function.pdf]

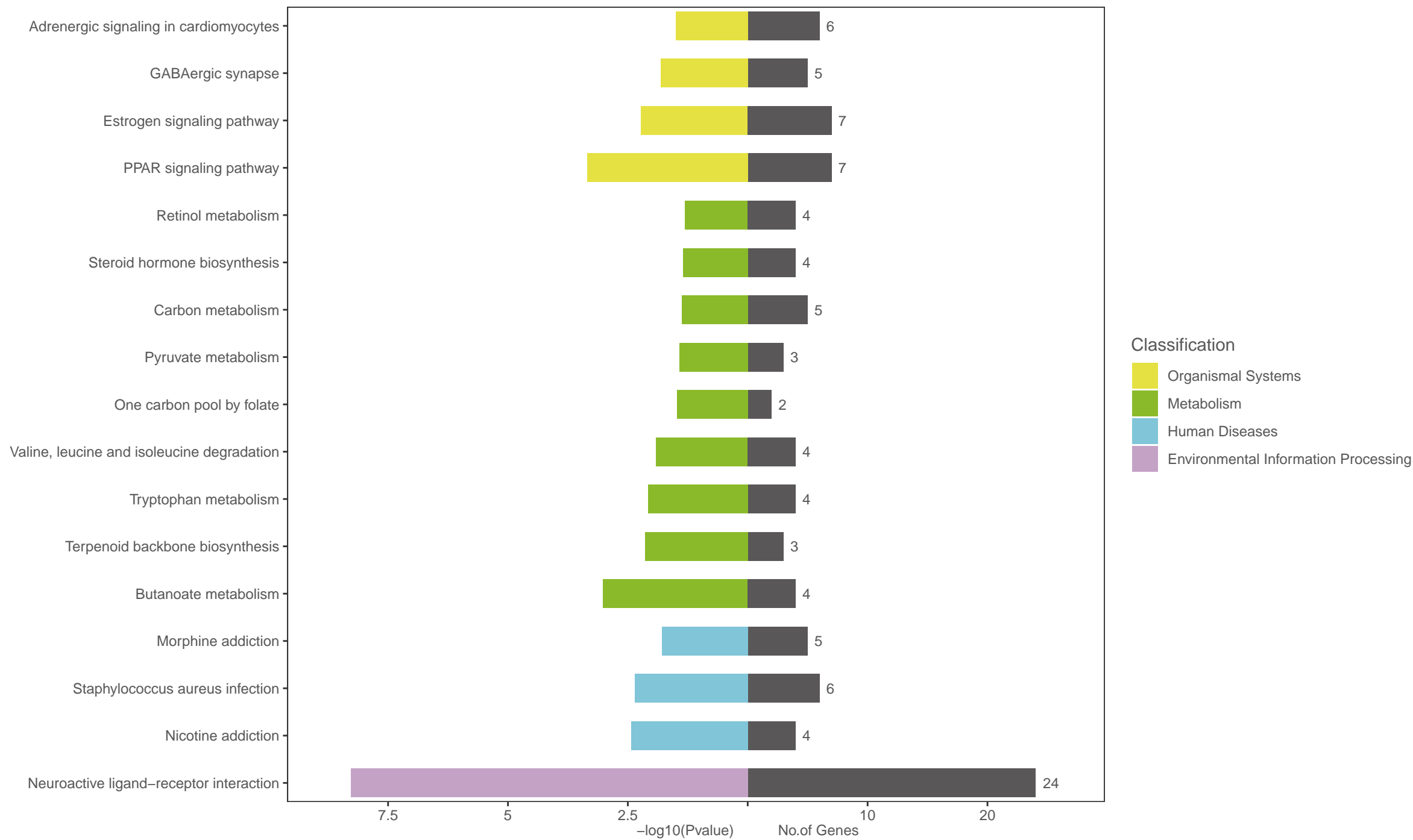

Supplement: Supplementary file 1 [file Presentation1.zip › Data/lncRNA/KEGG/Control--Treatment/kegg.pdf]

Statistics of Pathway Enrichment

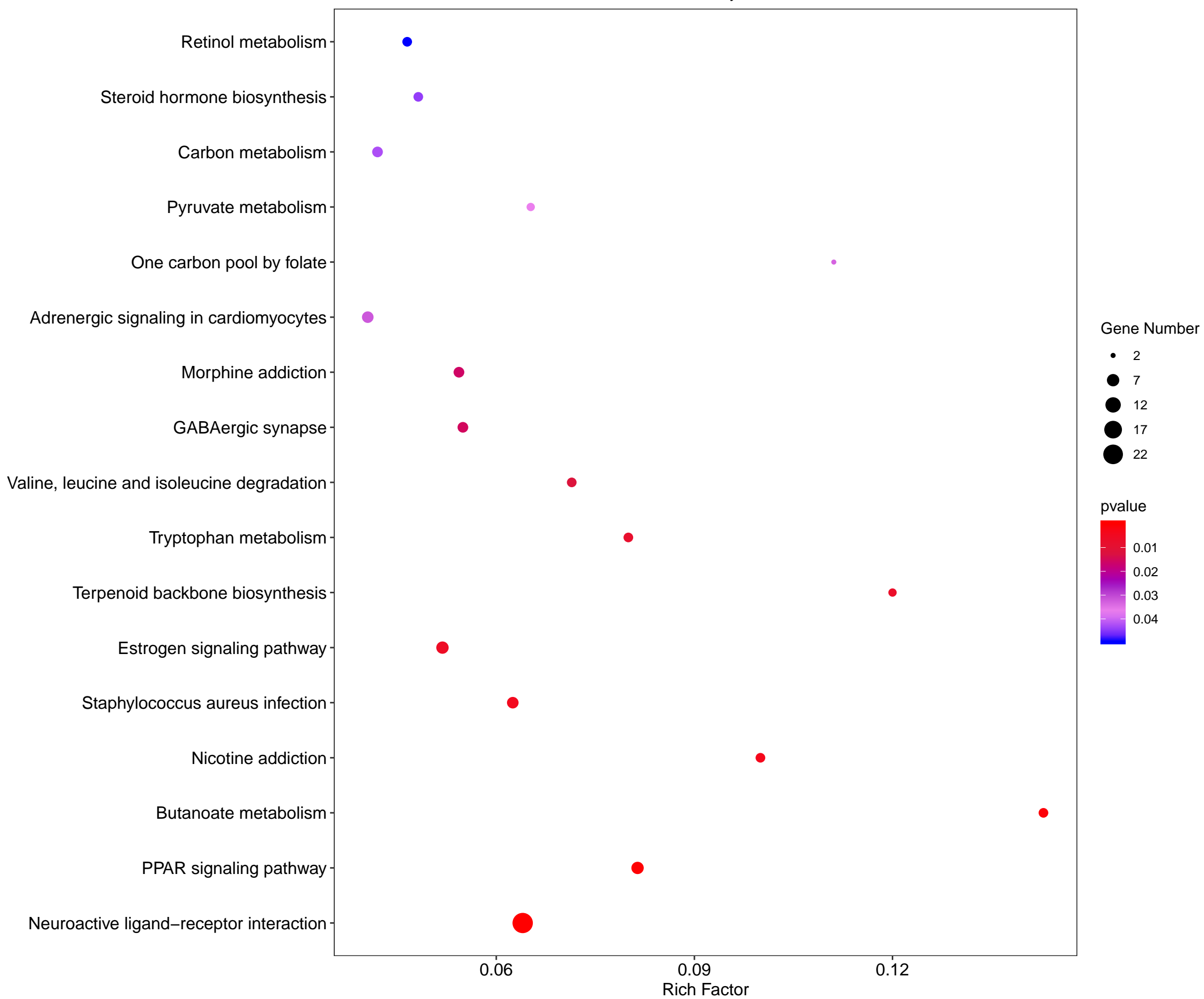

Supplement: Supplementary file 1 [file Presentation1.zip › Data/lncRNA/KEGG/Control--Treatment/kegg.point.pdf]

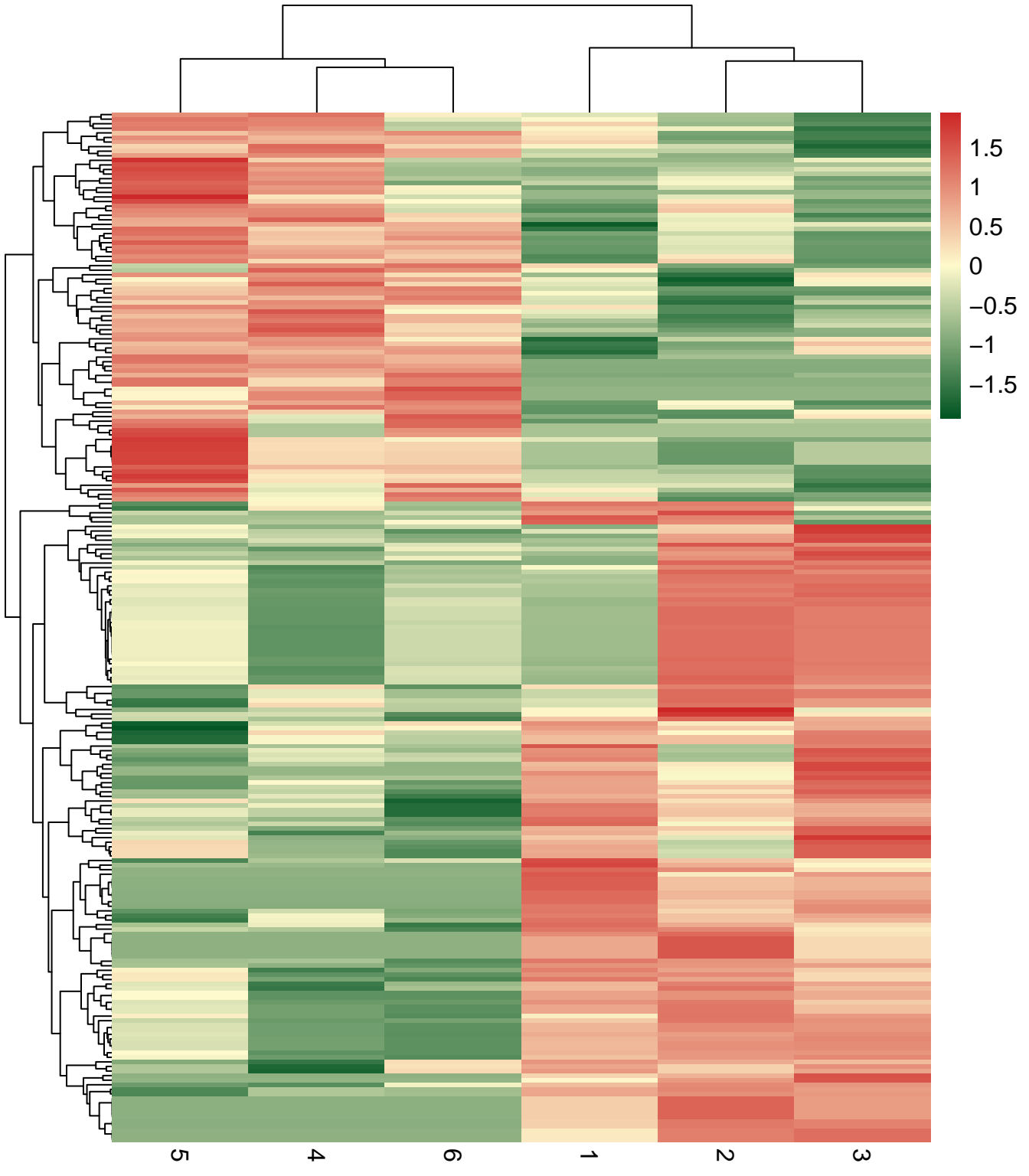

Supplement: Supplementary file 1 [file Presentation1.zip › Data/lncRNA/lncRNA_differential_expression/Control--Treatment/Control--Treatment.heatmap.lncRNA.pdf]

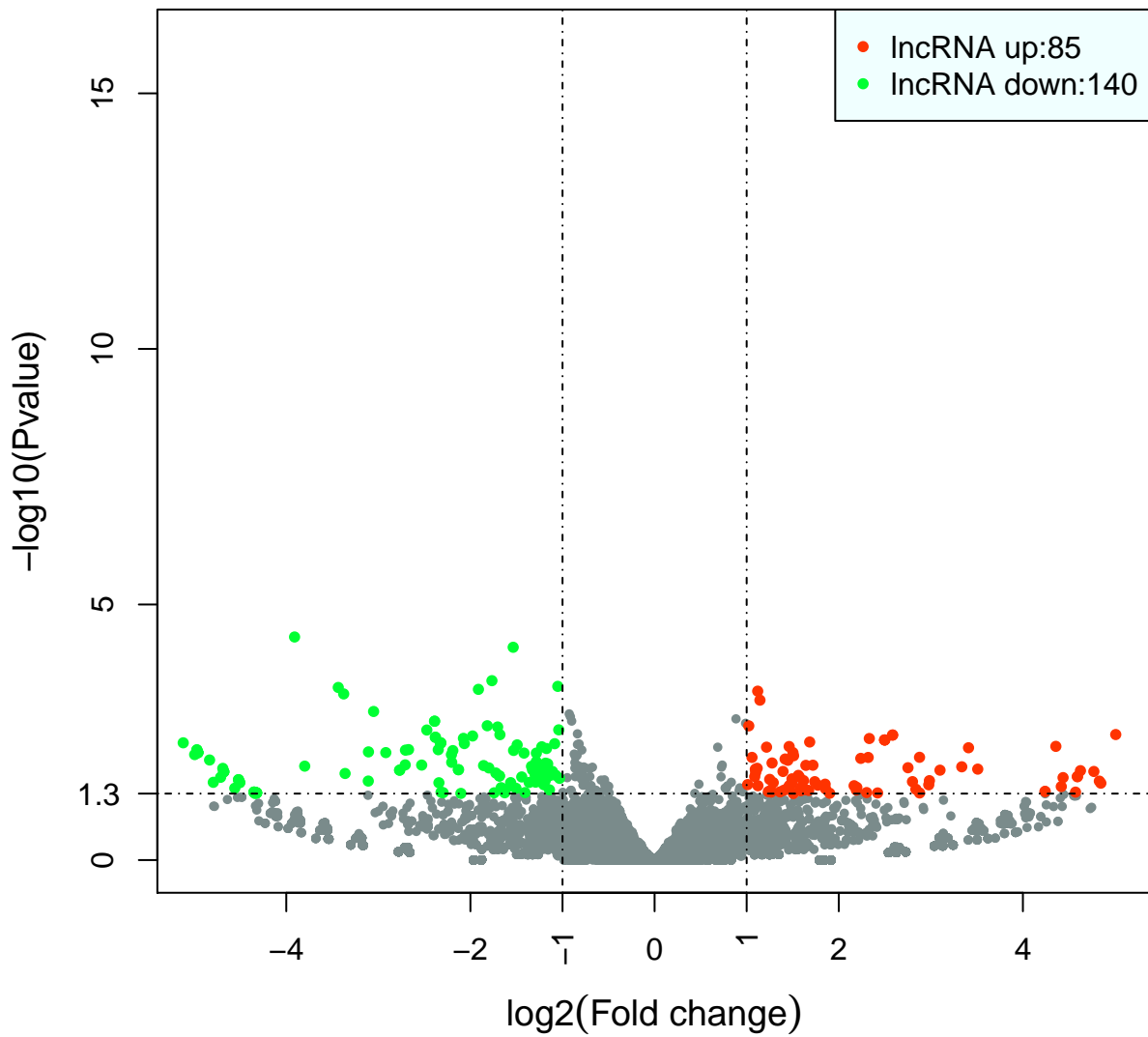

Supplement: Supplementary file 1 [file Presentation1.zip › Data/lncRNA/lncRNA_differential_expression/Control--Treatment/Control--Treatment.volcano.lncRNA.pdf]

gene  
lncRNAs

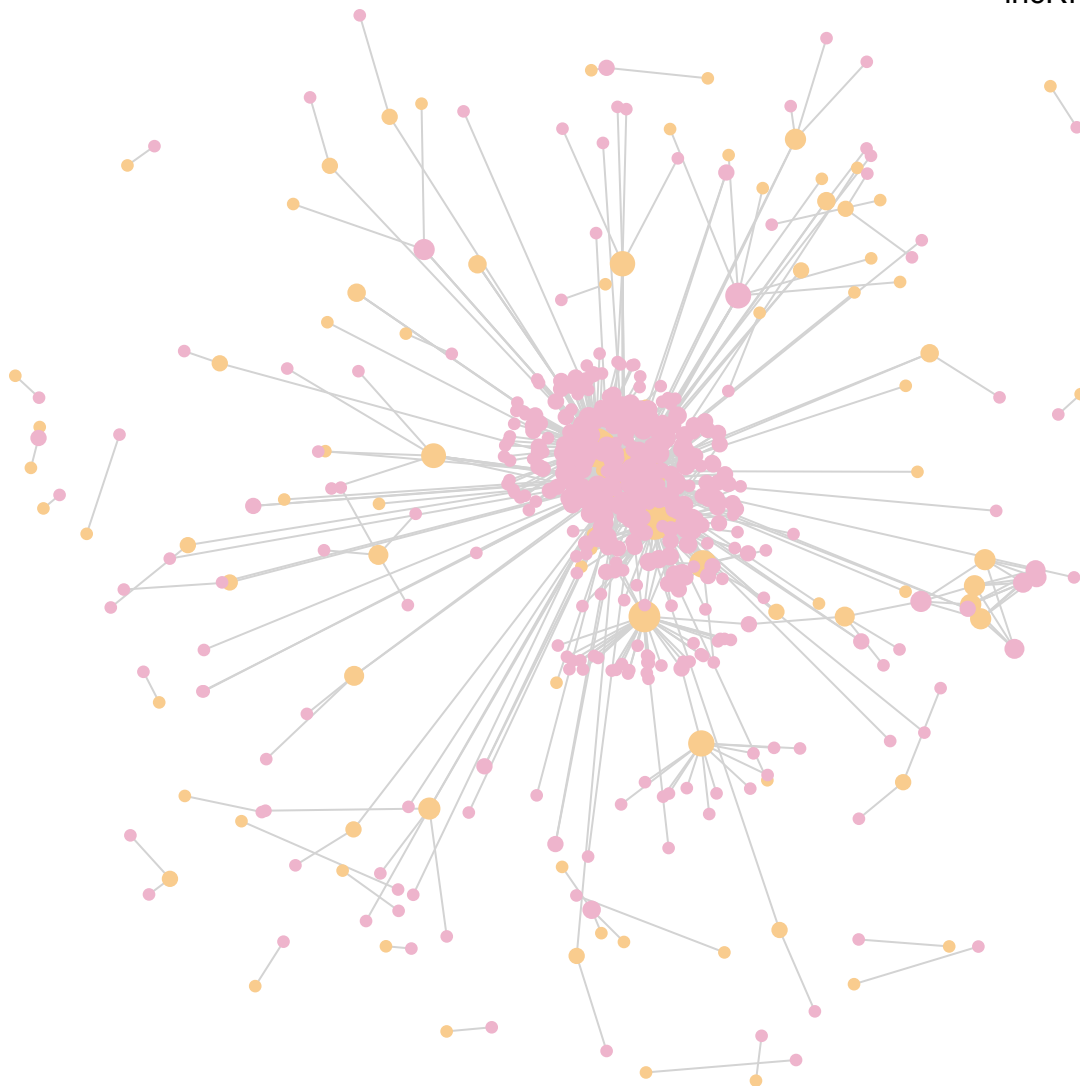

Supplement: Supplementary file 1 [file Presentation1.zip › Data/lncRNA/lncRNA_target/Control--Treatment/network.pdf]

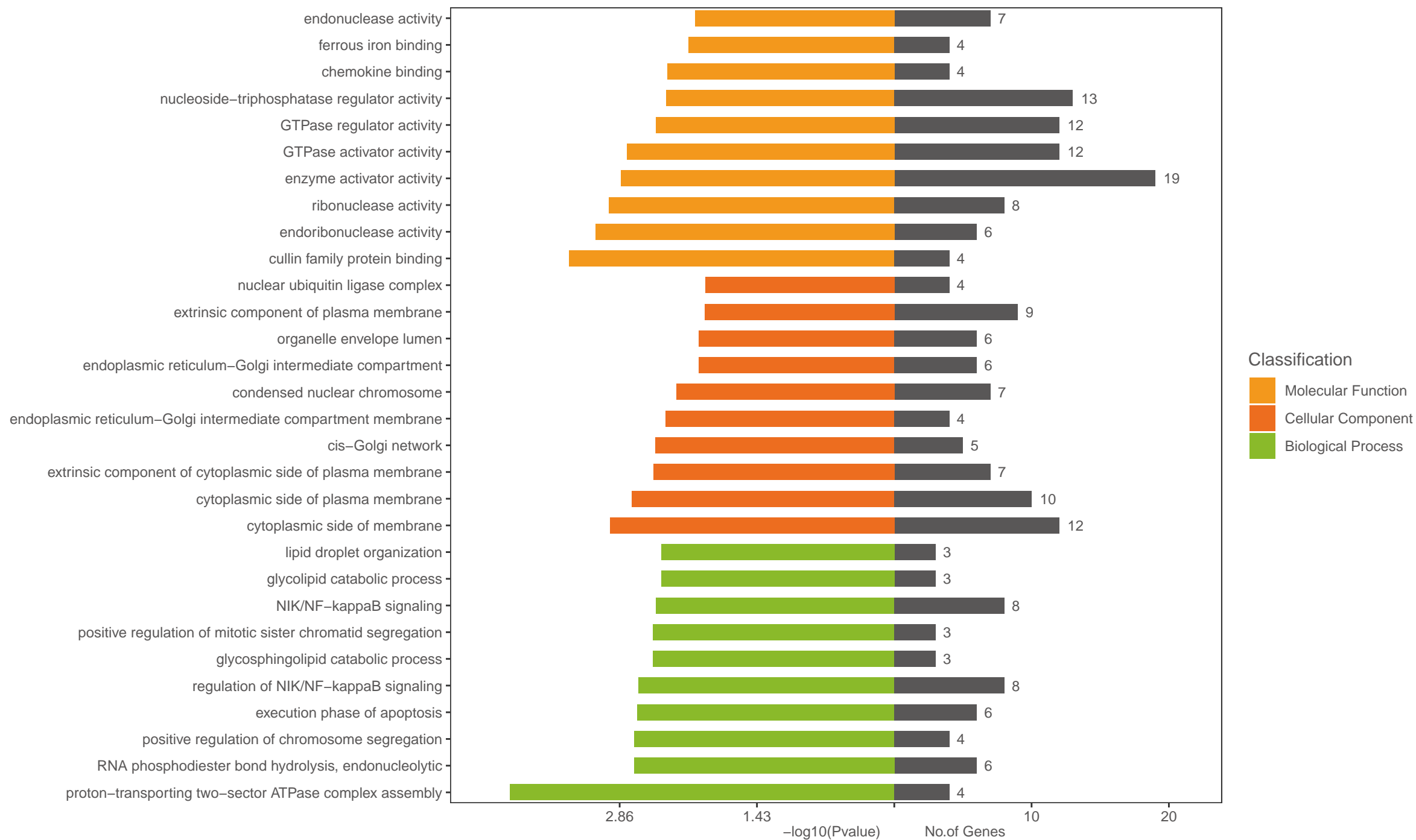

Supplement: Supplementary file 1 [file Presentation1.zip › Data/lncRNA/lncRNA_target_GO/Control--Treatment/go.pdf]

Statistics of Pathway Enrichment

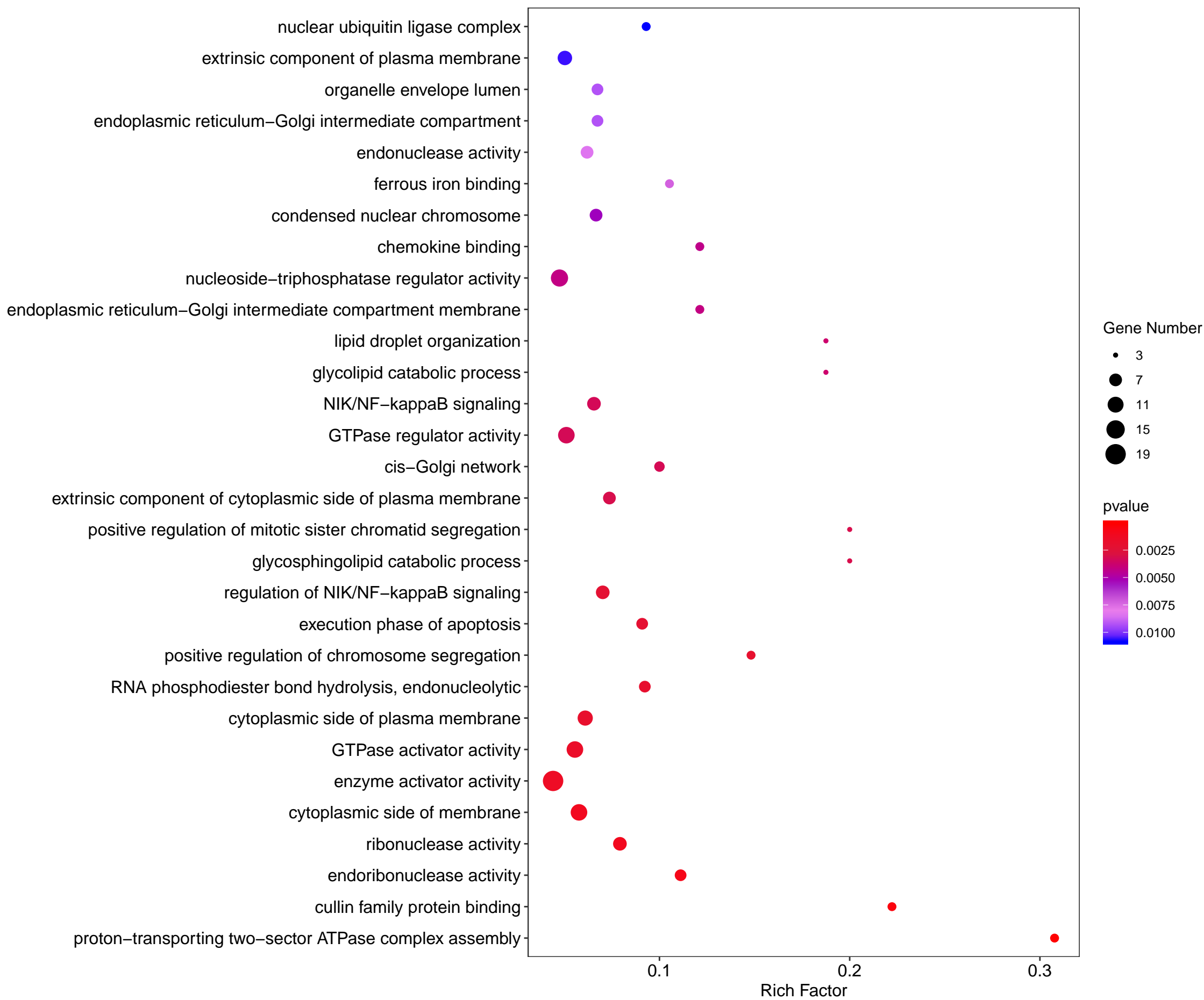

Supplement: Supplementary file 1 [file Presentation1.zip › Data/lncRNA/lncRNA_target_GO/Control--Treatment/go.point.pdf]

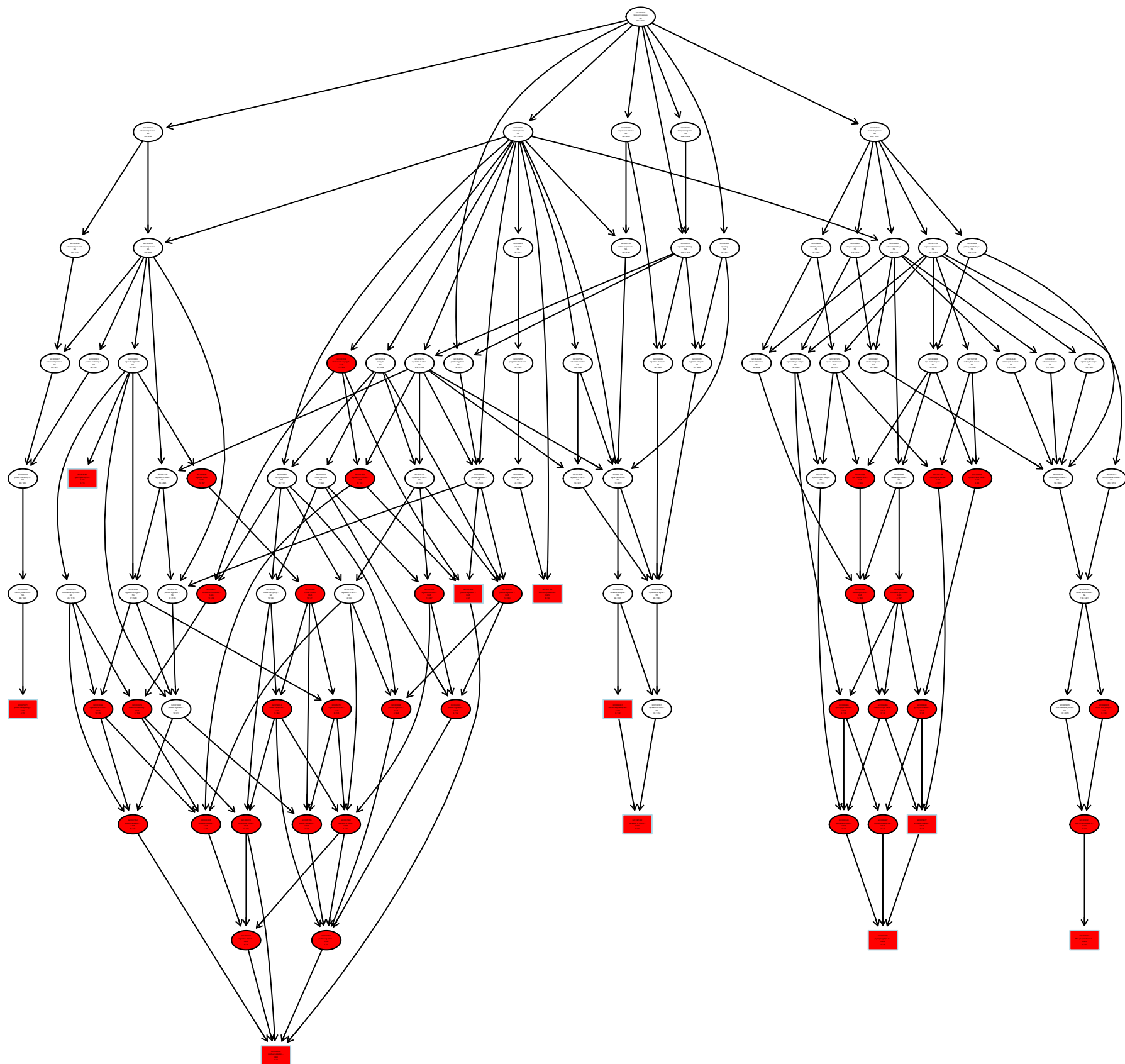

Supplement: Supplementary file 1 [file Presentation1.zip › Data/lncRNA/lncRNA_target_GO/Control--Treatment/GO-Biological_Process.pdf]

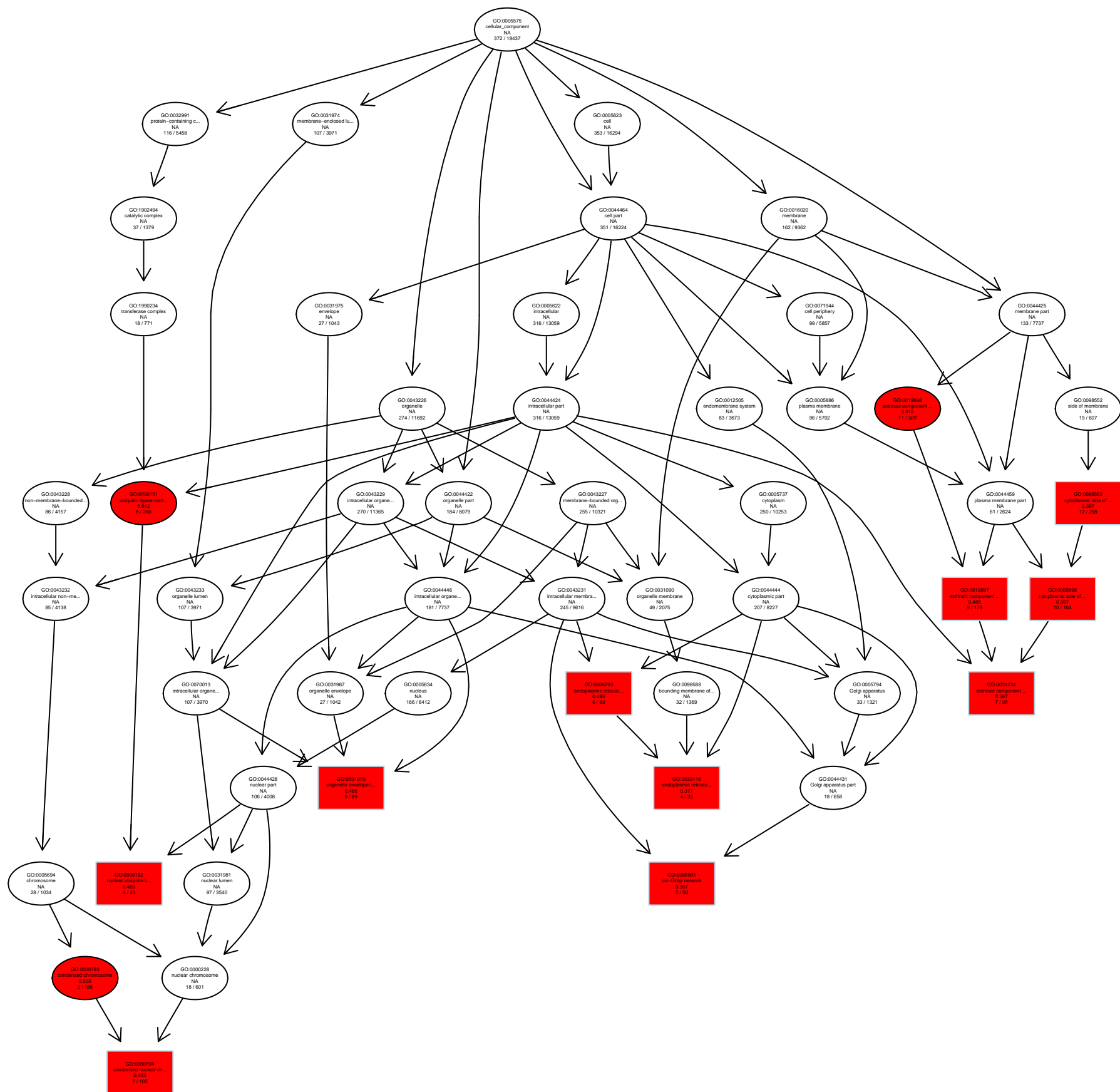

Supplement: Supplementary file 1 [file Presentation1.zip › Data/lncRNA/lncRNA_target_GO/Control--Treatment/GO-Cellular_Component.pdf]

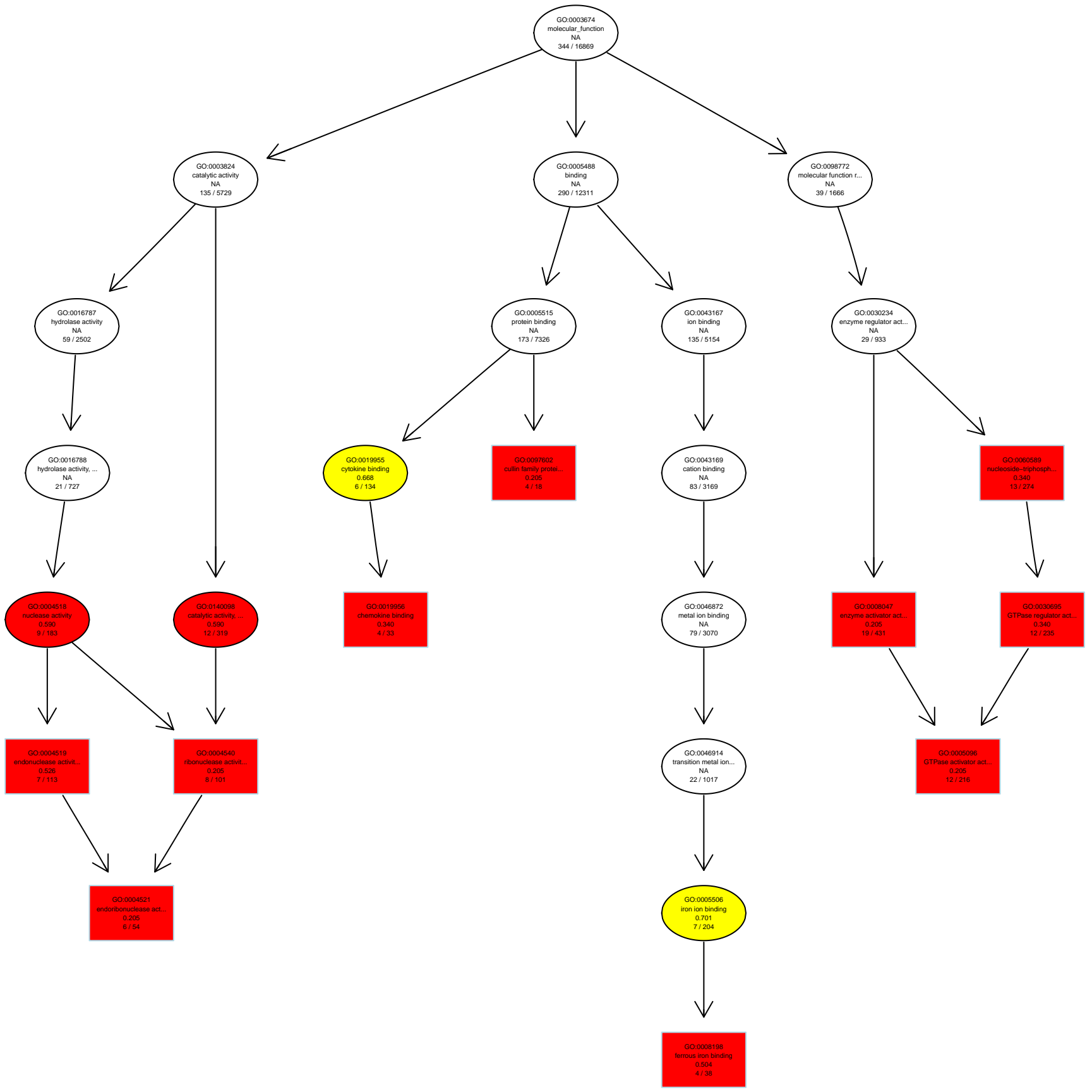

Supplement: Supplementary file 1 [file Presentation1.zip › Data/lncRNA/lncRNA_target_GO/Control--Treatment/GO-Molecular_Function.pdf]

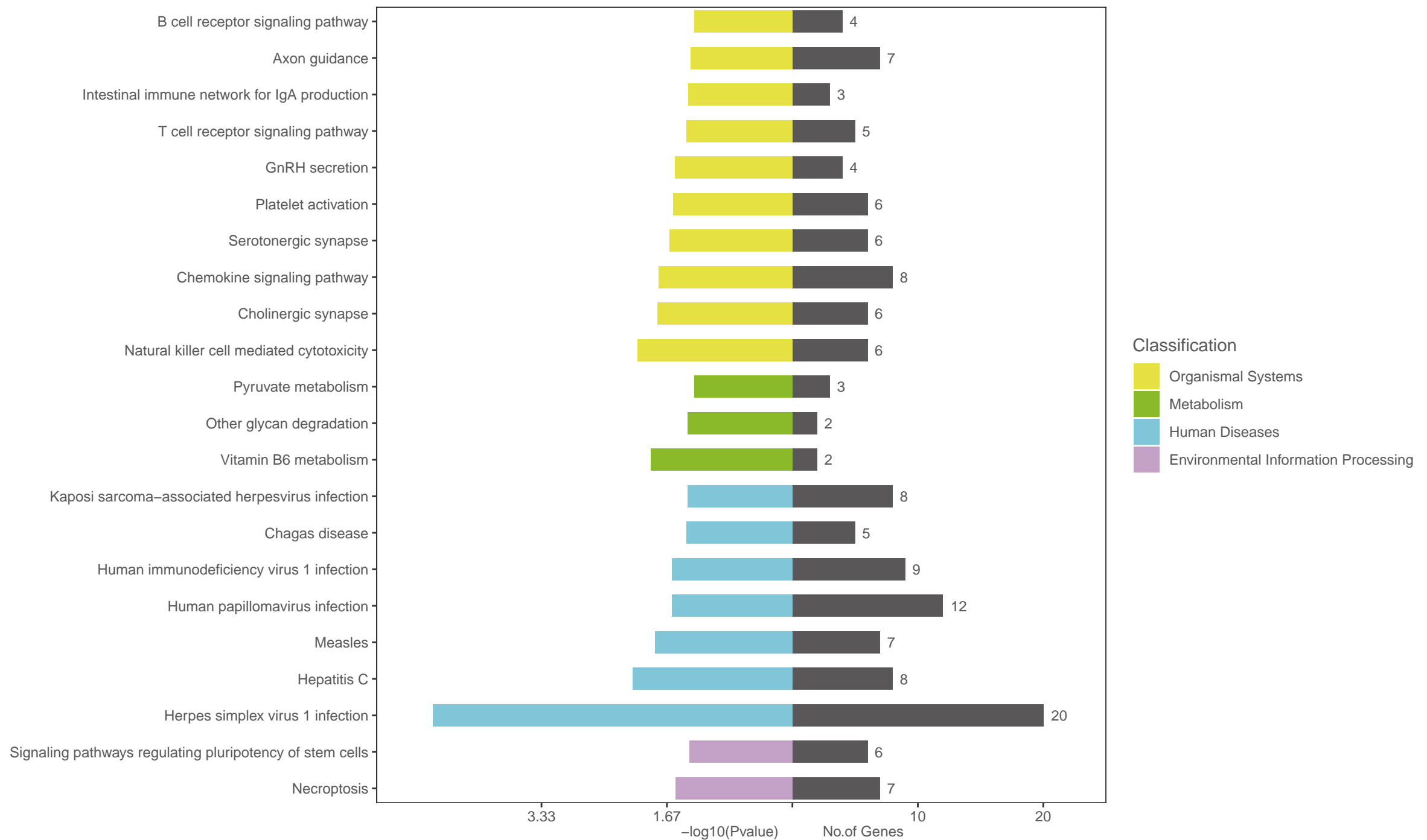

Supplement: Supplementary file 1 [file Presentation1.zip › Data/lncRNA/lncRNA_target_KEGG/Control--Treatment/kegg.pdf]

Statistics of Pathway Enrichment

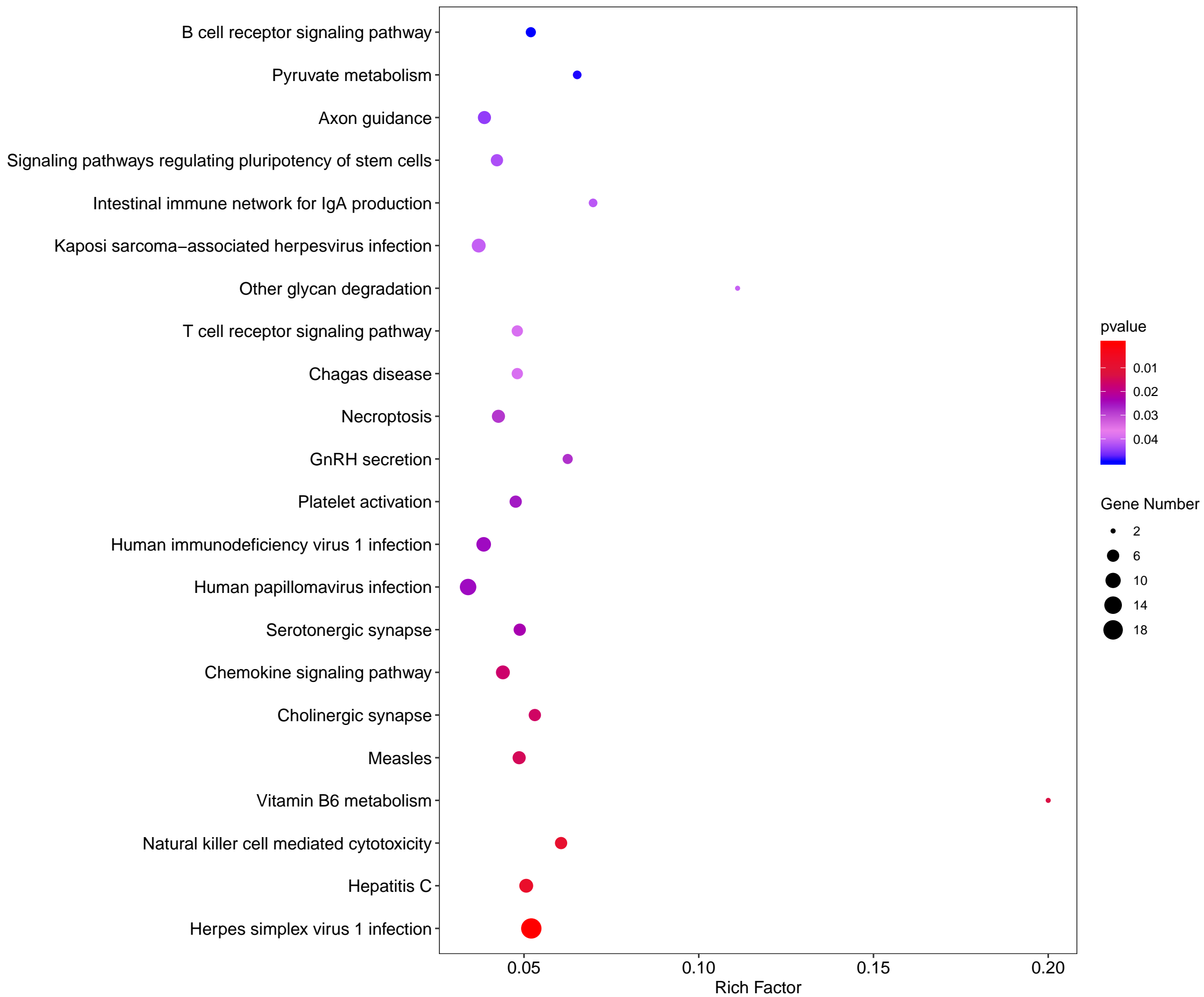

Supplement: Supplementary file 1 [file Presentation1.zip › Data/lncRNA/lncRNA_target_KEGG/Control--Treatment/kegg.point.pdf]

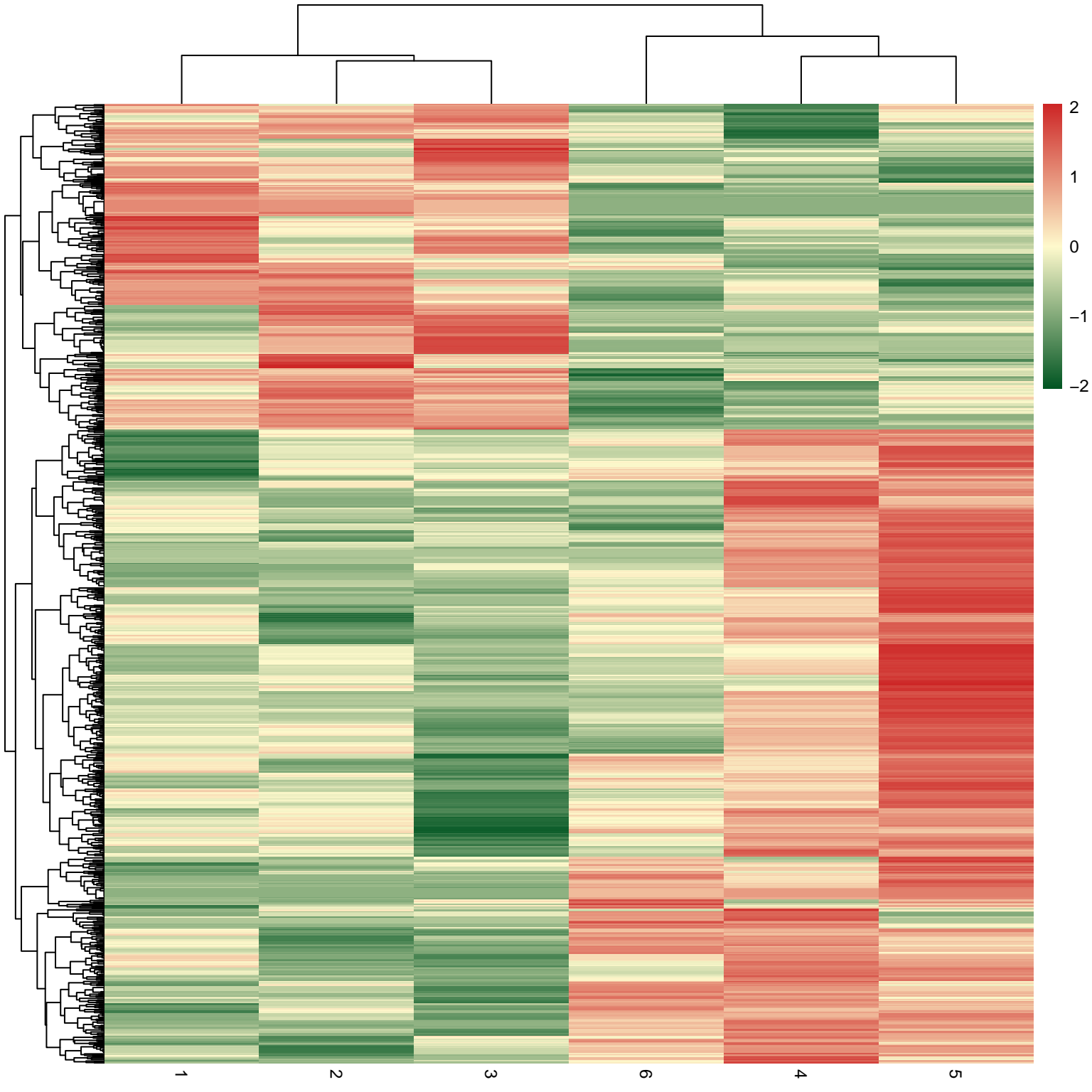

Supplement: Supplementary file 1 [file Presentation1.zip › Data/lncRNA/mRNA_differential_expression/Control--Treatment/Control--Treatment.heatmap.mRNA.pdf]

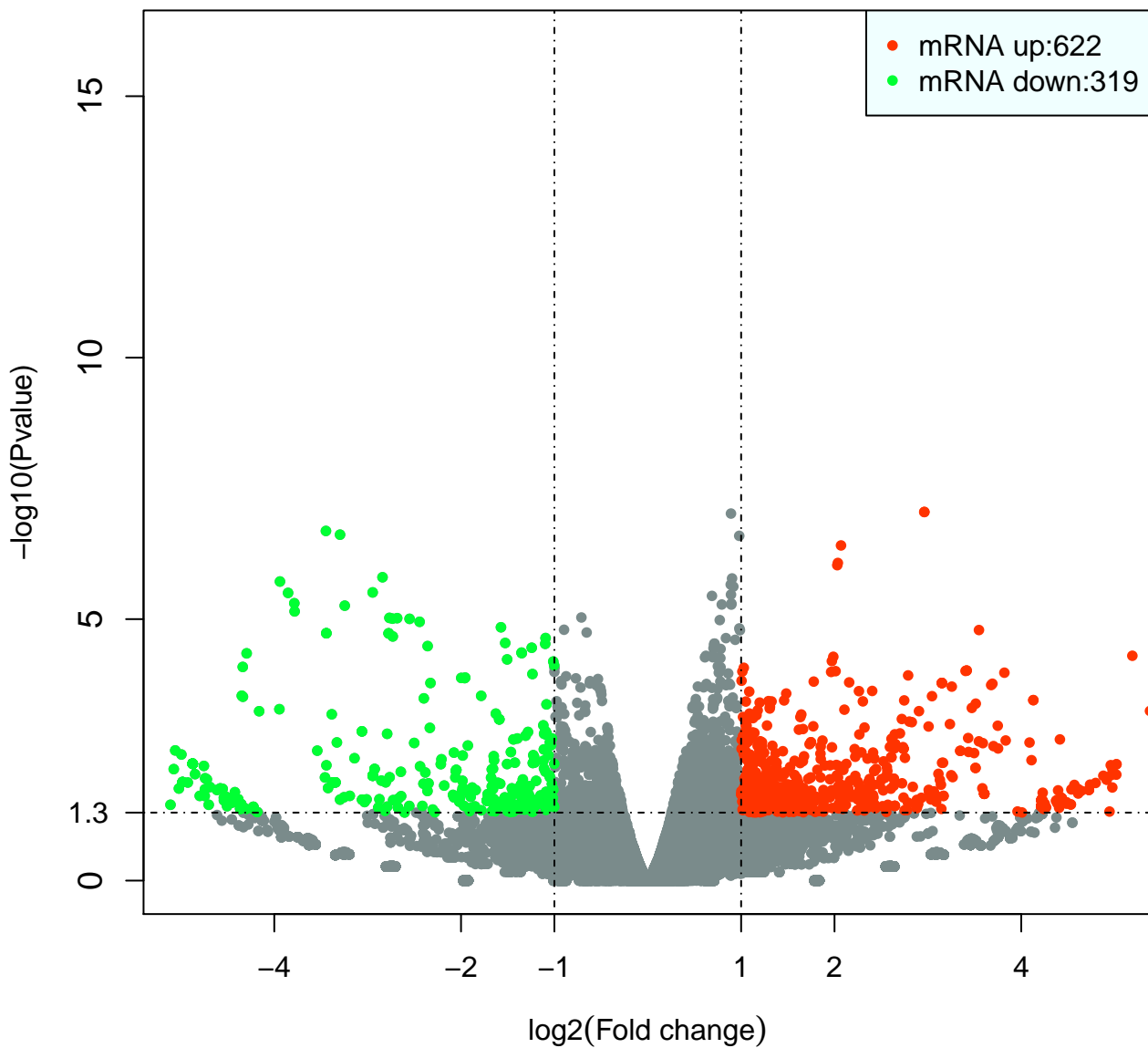

Supplement: Supplementary file 1 [file Presentation1.zip › Data/lncRNA/mRNA_differential_expression/Control--Treatment/Control--Treatment.volcano.mRNA.pdf]

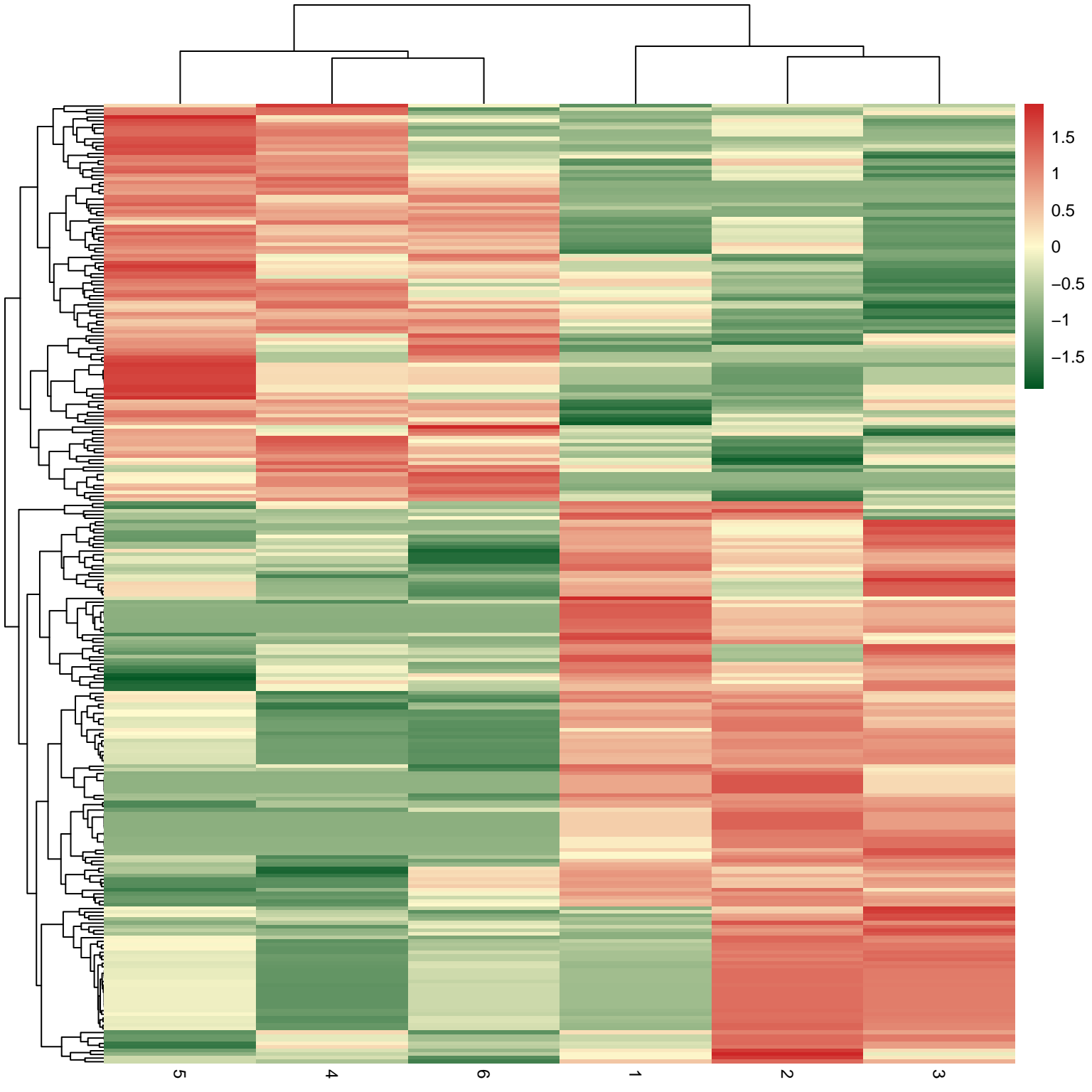

Supplement: Supplementary file 1 [file Presentation1.zip › Data/lncRNA/ncRNA_differential_expression/Control--Treatment/Control--Treatment.heatmap.ncRNA.pdf]

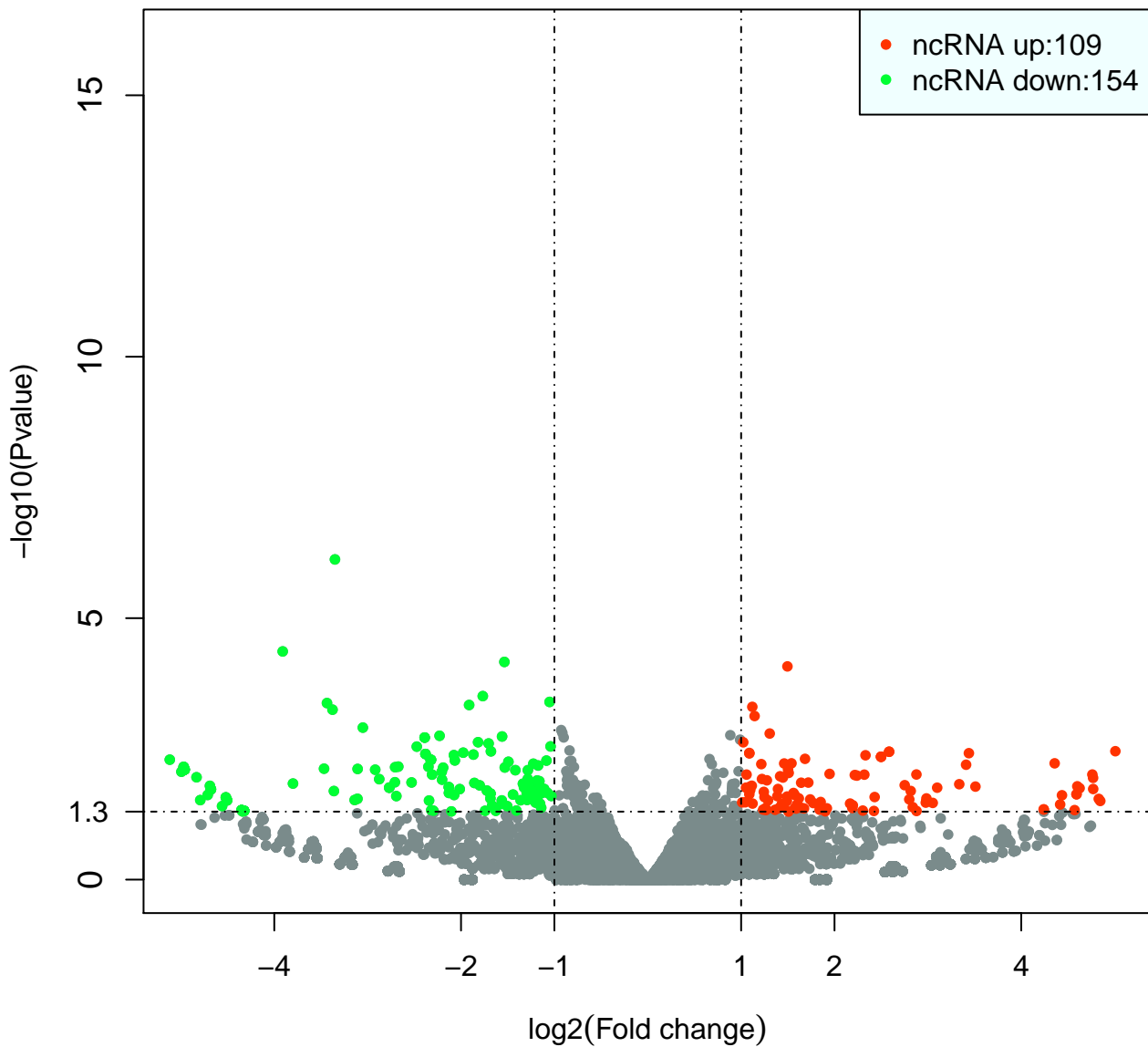

Supplement: Supplementary file 1 [file Presentation1.zip › Data/lncRNA/ncRNA_differential_expression/Control--Treatment/Control--Treatment.volcano.ncRNA.pdf]

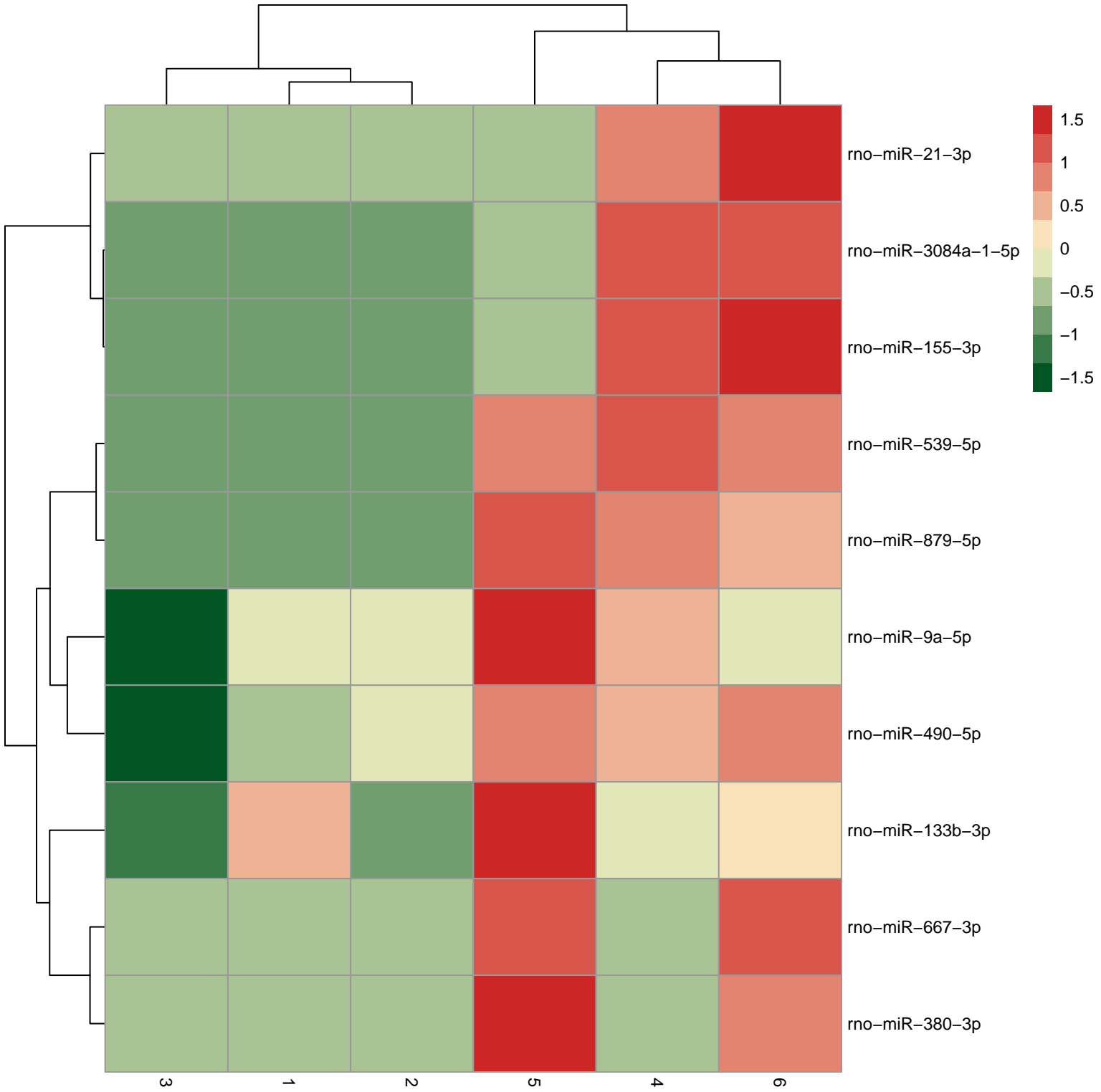

Supplement: Supplementary file 1 [file Presentation1.zip › Data/microRN/differential_expression/Control--Treatment/Control--Treatment.heatmap.miRNA.pdf]

# The Difference of miRNA Profiles

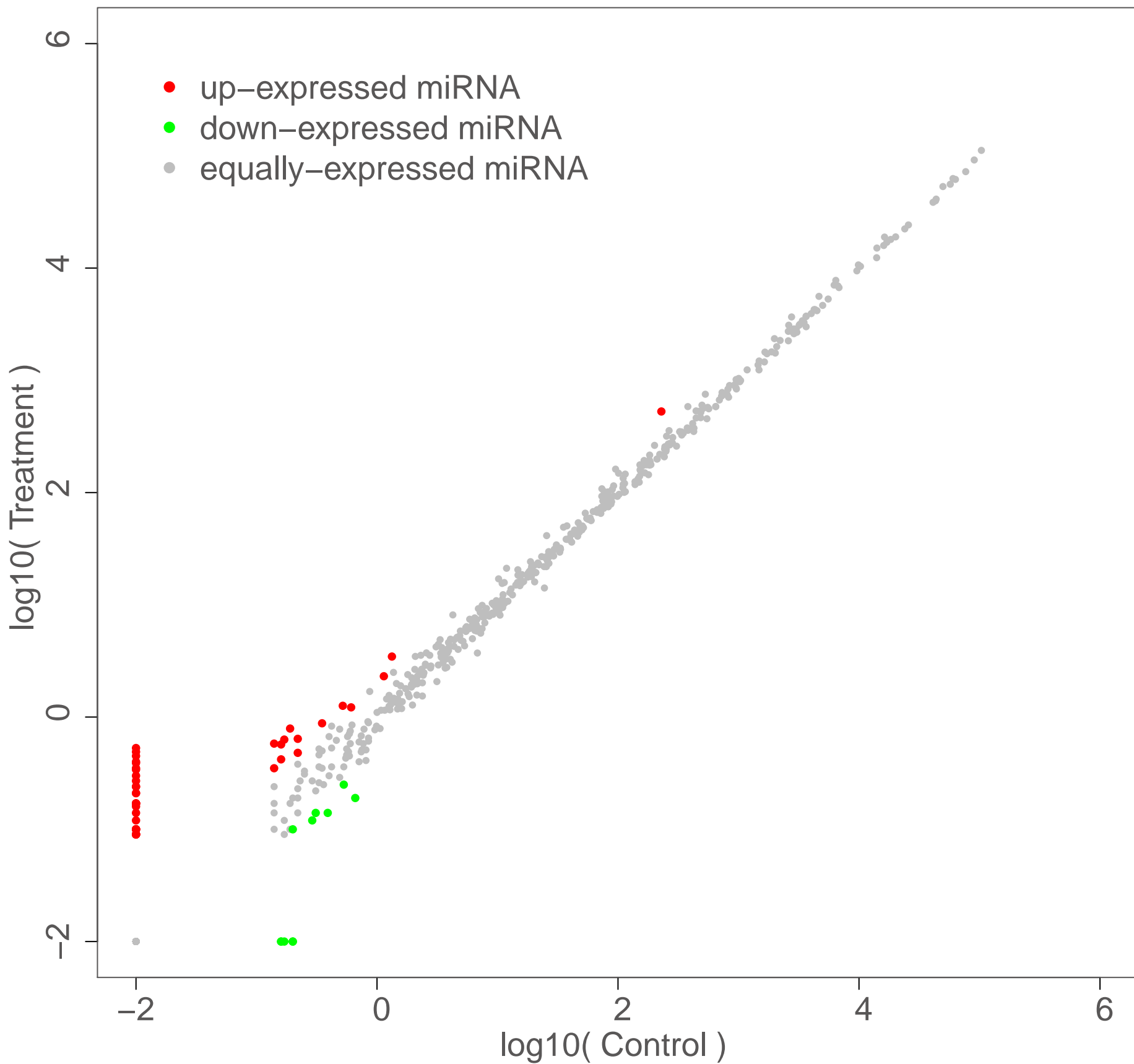

Supplement: Supplementary file 1 [file Presentation1.zip › Data/microRN/differential_expression/Control--Treatment/Control--Treatment.miRNA.point.pdf]

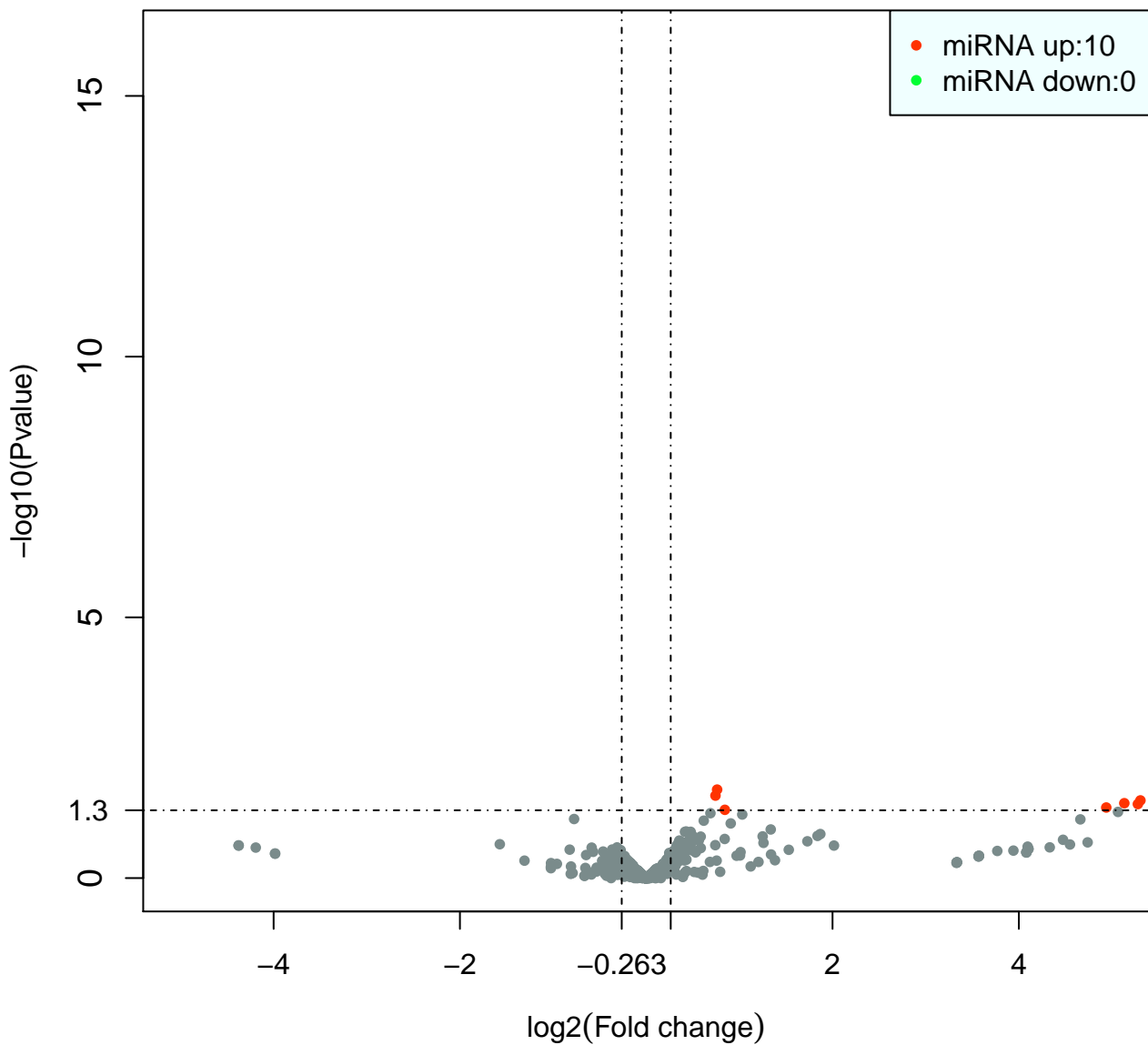

Supplement: Supplementary file 1 [file Presentation1.zip › Data/microRN/differential_expression/Control--Treatment/Control--Treatment.miRNA.volcano.pdf]

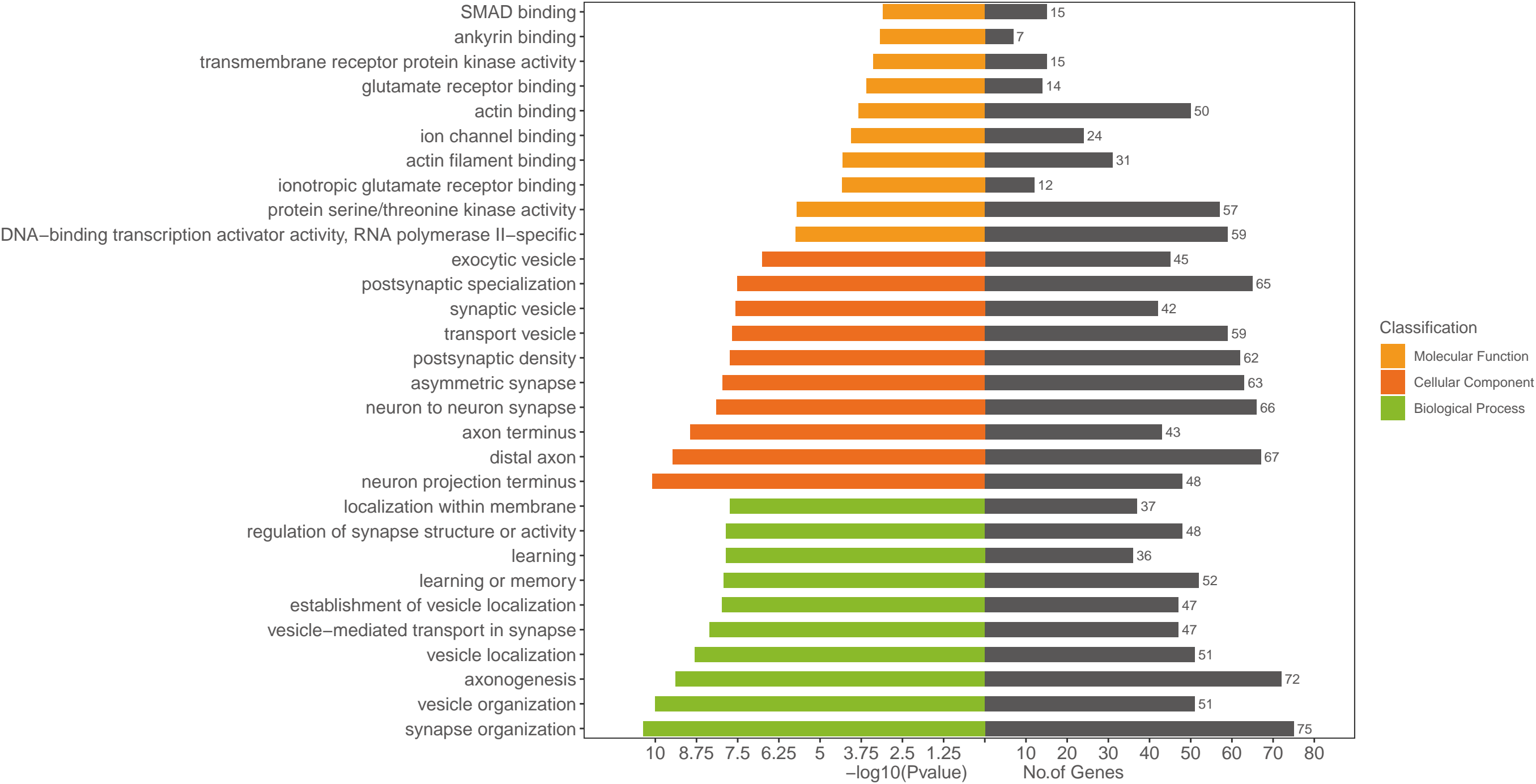

Supplement: Supplementary file 1 [file Presentation1.zip › Data/microRN/GO/Control--Treatment/go.pdf]

Statistics of Pathway Enrichment

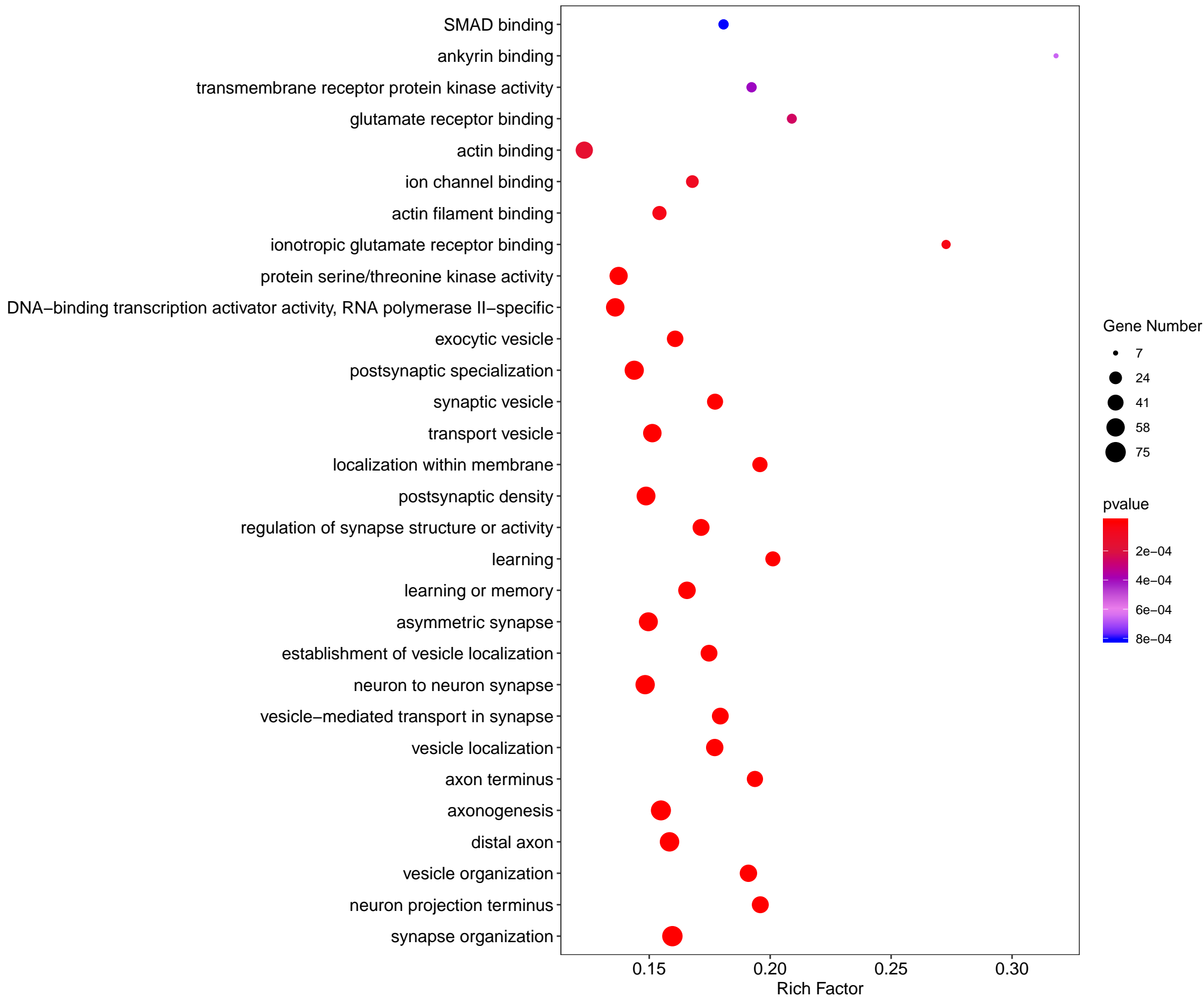

Supplement: Supplementary file 1 [file Presentation1.zip › Data/microRN/GO/Control--Treatment/go.point.pdf]

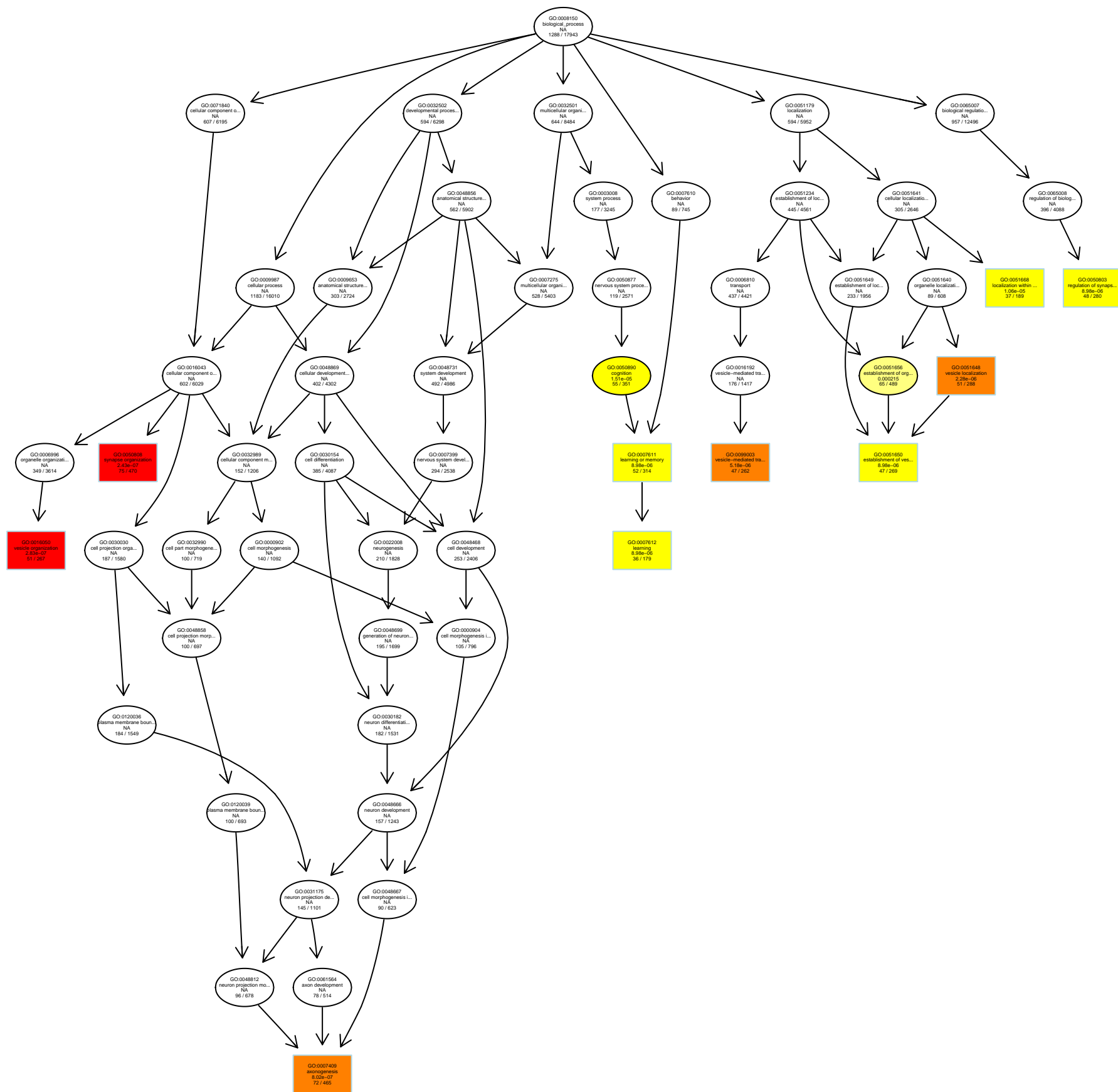

Supplement: Supplementary file 1 [file Presentation1.zip › Data/microRN/GO/Control--Treatment/GO-Biological_Process.pdf]

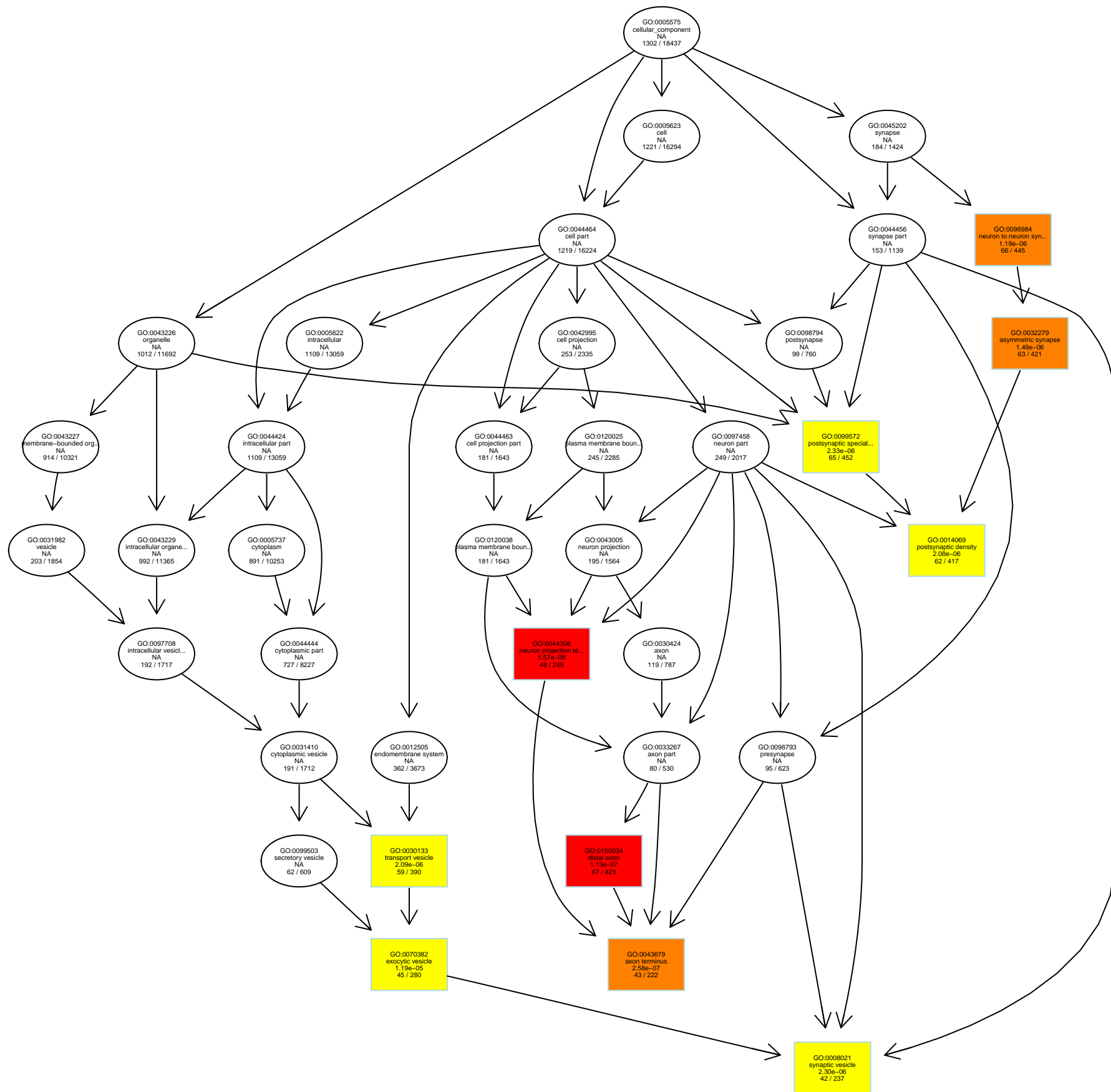

Supplement: Supplementary file 1 [file Presentation1.zip › Data/microRN/GO/Control--Treatment/GO-Cellular_Component.pdf]

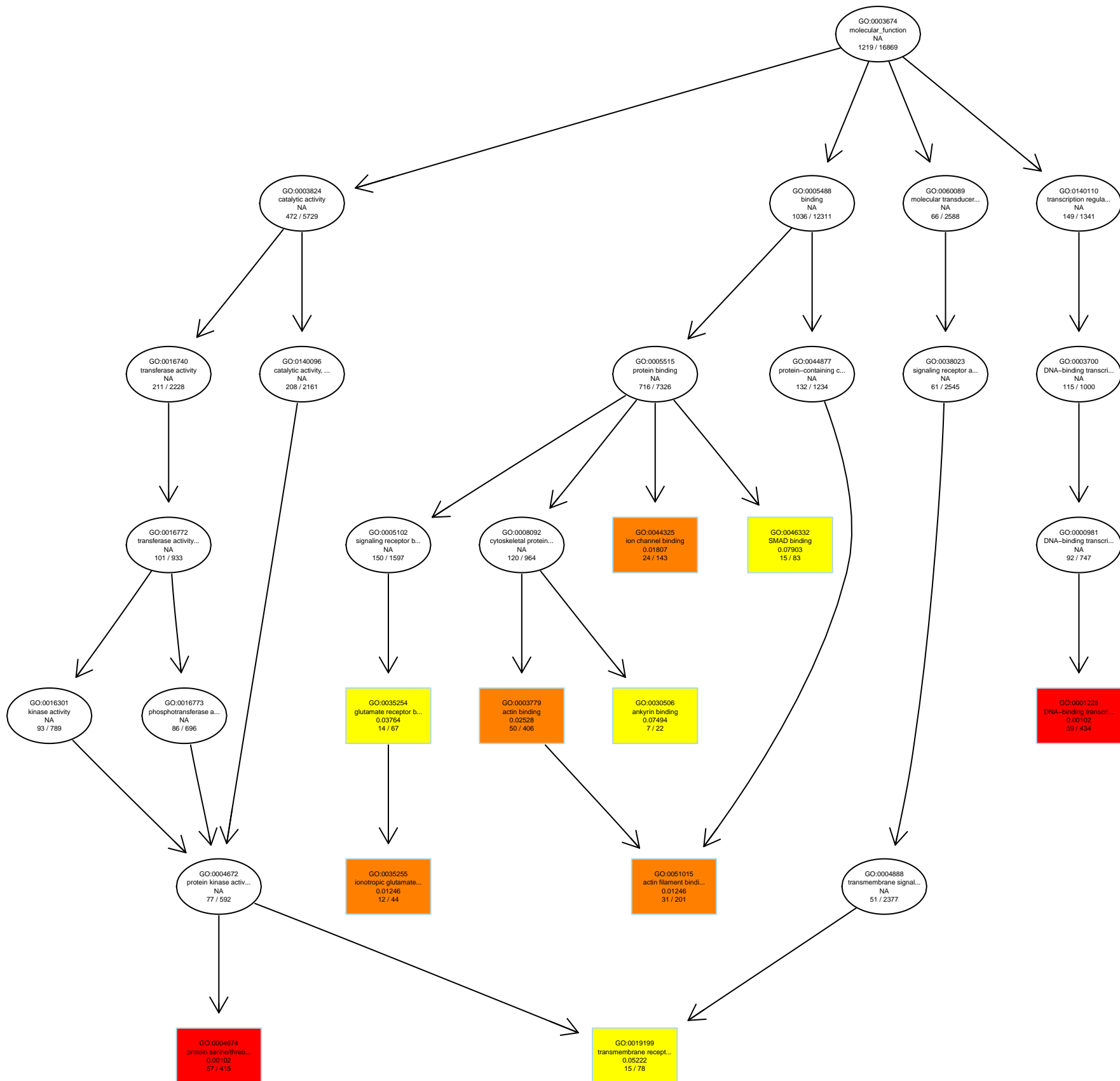

Supplement: Supplementary file 1 [file Presentation1.zip › Data/microRN/GO/Control--Treatment/GO-Molecular_Function.pdf]

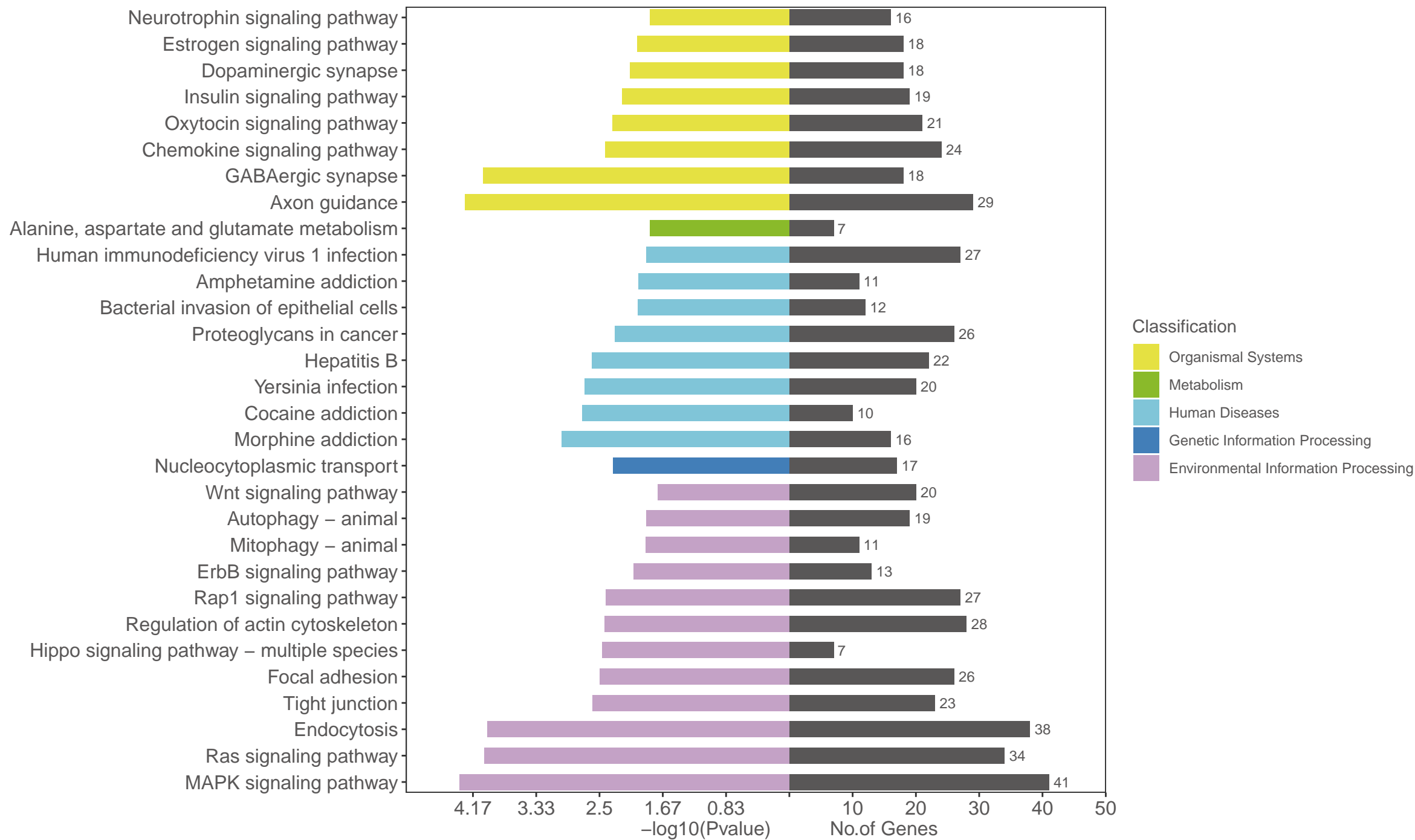

Supplement: Supplementary file 1 [file Presentation1.zip › Data/microRN/KEGG/Control--Treatment/kegg.pdf]

Statistics of Pathway Enrichment

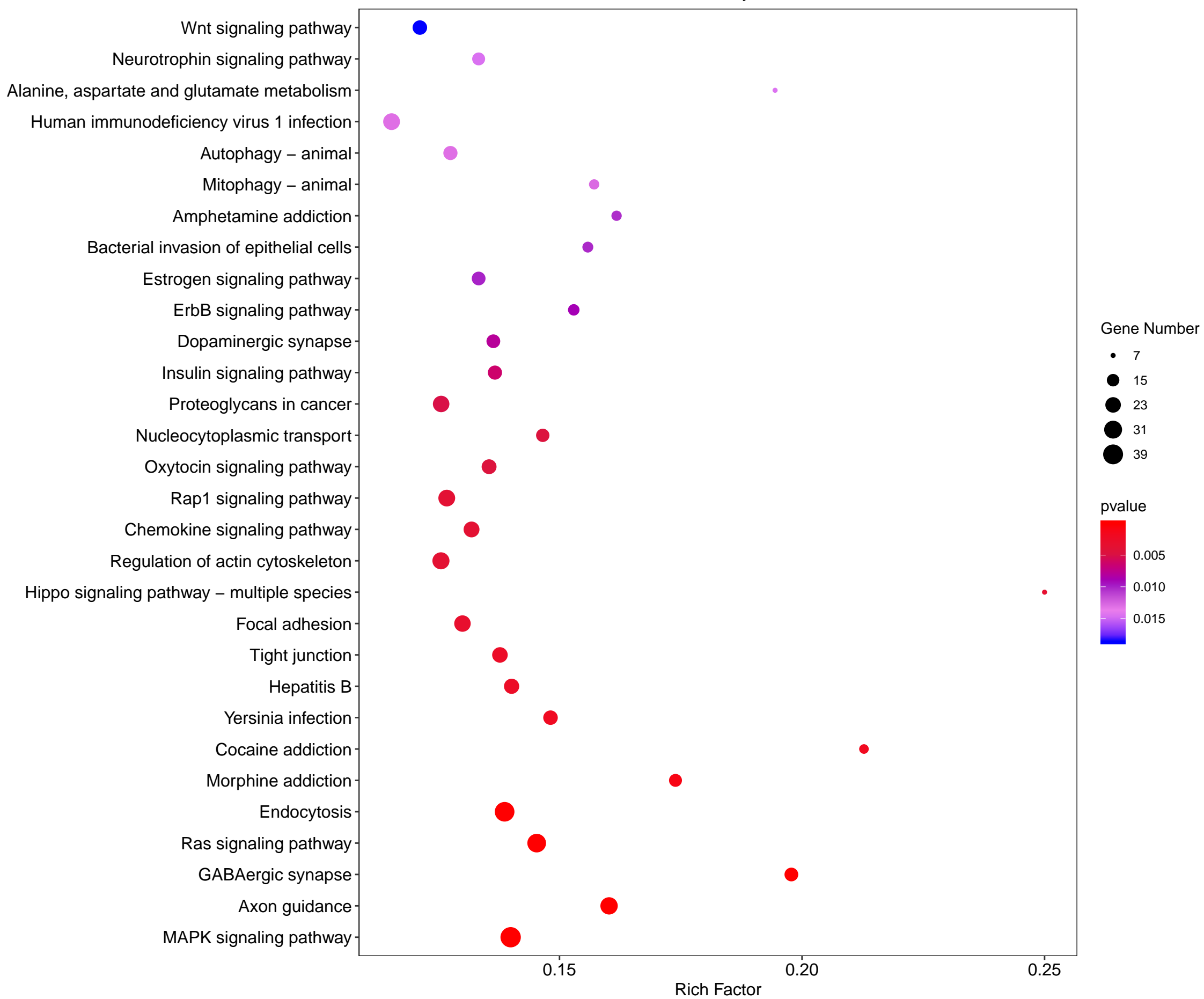

Supplement: Supplementary file 1 [file Presentation1.zip › Data/microRN/KEGG/Control--Treatment/kegg.point.pdf]

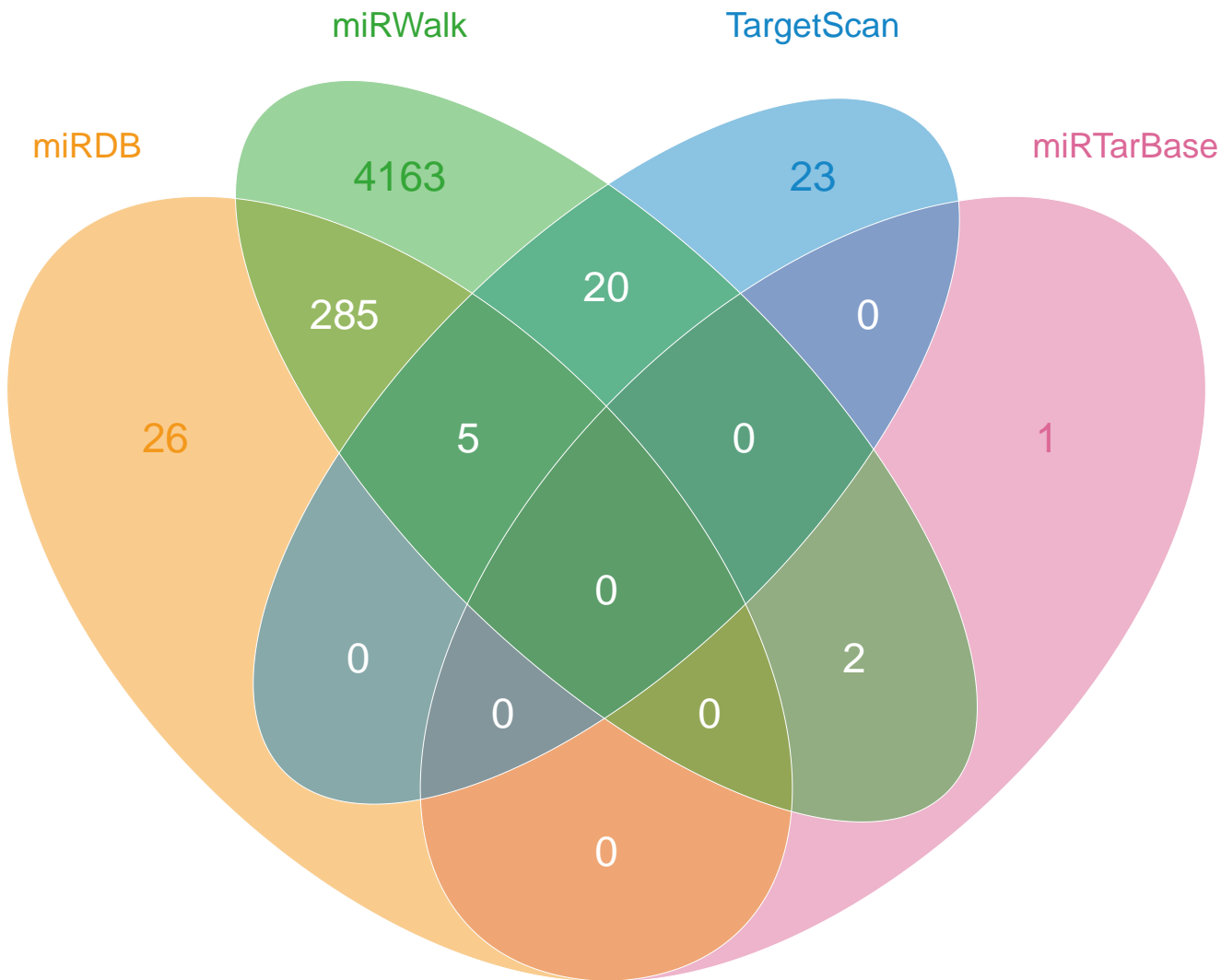

Supplement: Supplementary file 1 [file Presentation1.zip › Data/microRN/miR_target/Control--Treatment/result/rno-miR-133b-3p/rno-miR-133b-3p.pdf]

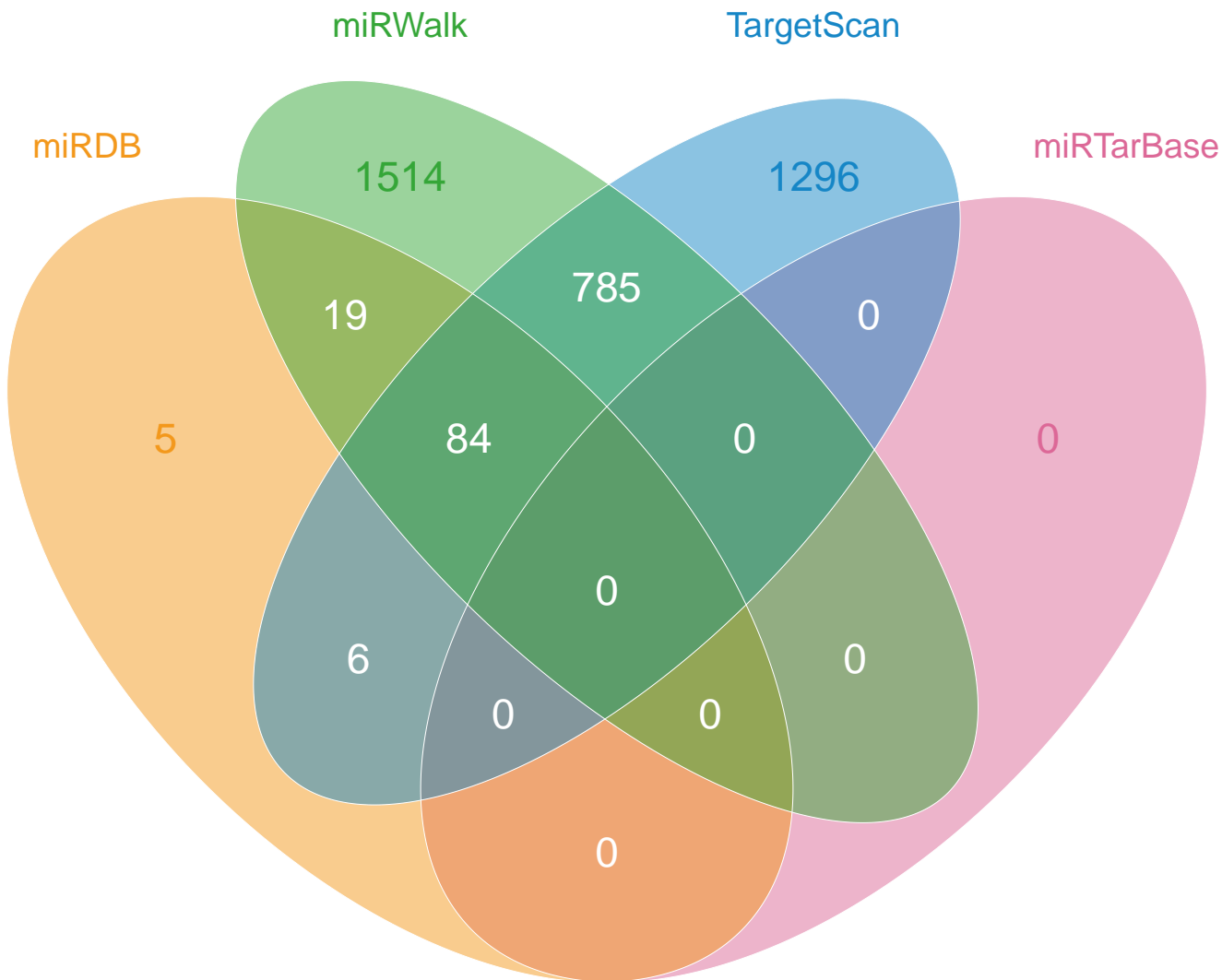

Supplement: Supplementary file 1 [file Presentation1.zip › Data/microRN/miR_target/Control--Treatment/result/rno-miR-155-3p/rno-miR-155-3p.pdf]

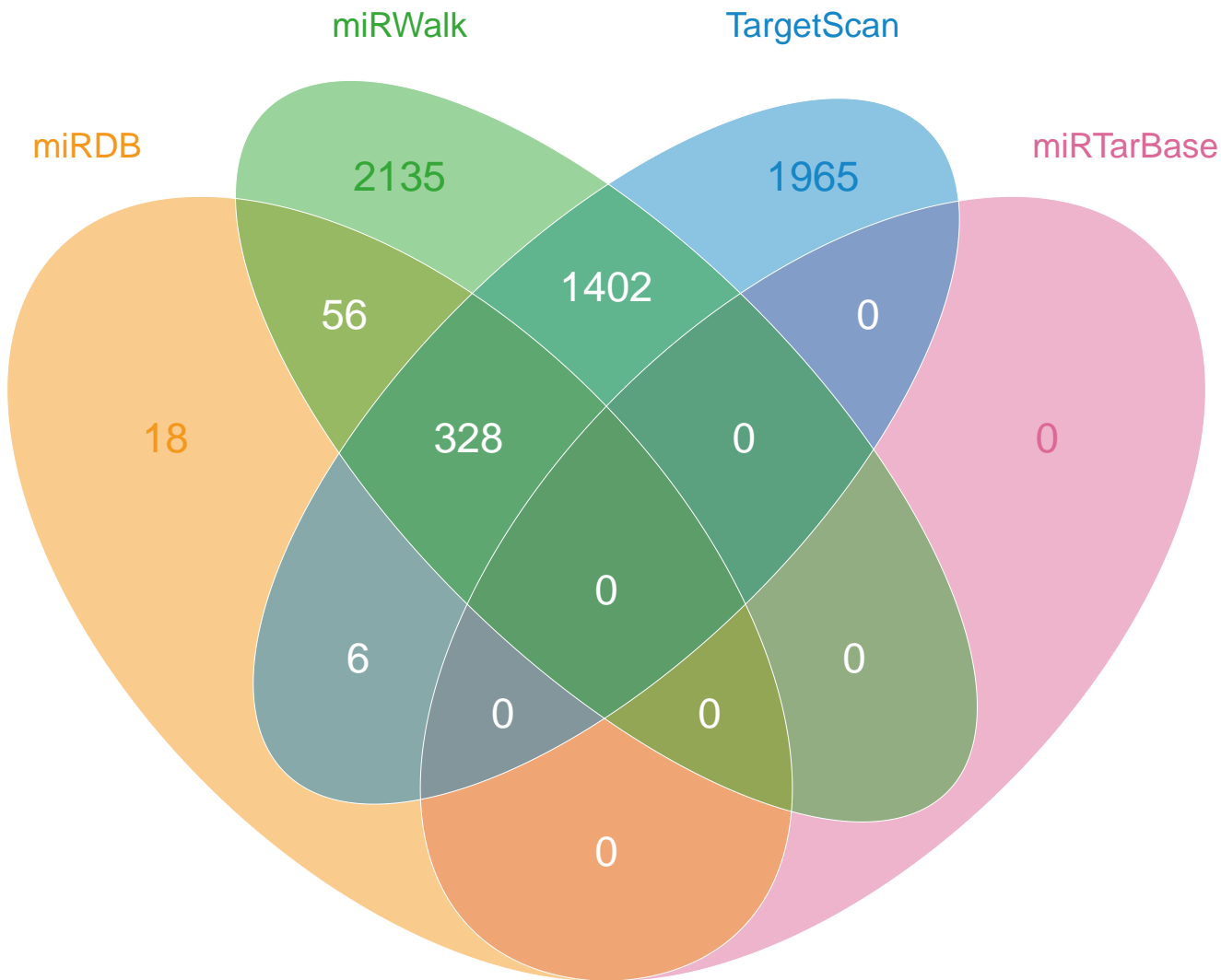

Supplement: Supplementary file 1 [file Presentation1.zip › Data/microRN/miR_target/Control--Treatment/result/rno-miR-21-3p/rno-miR-21-3p.pdf]

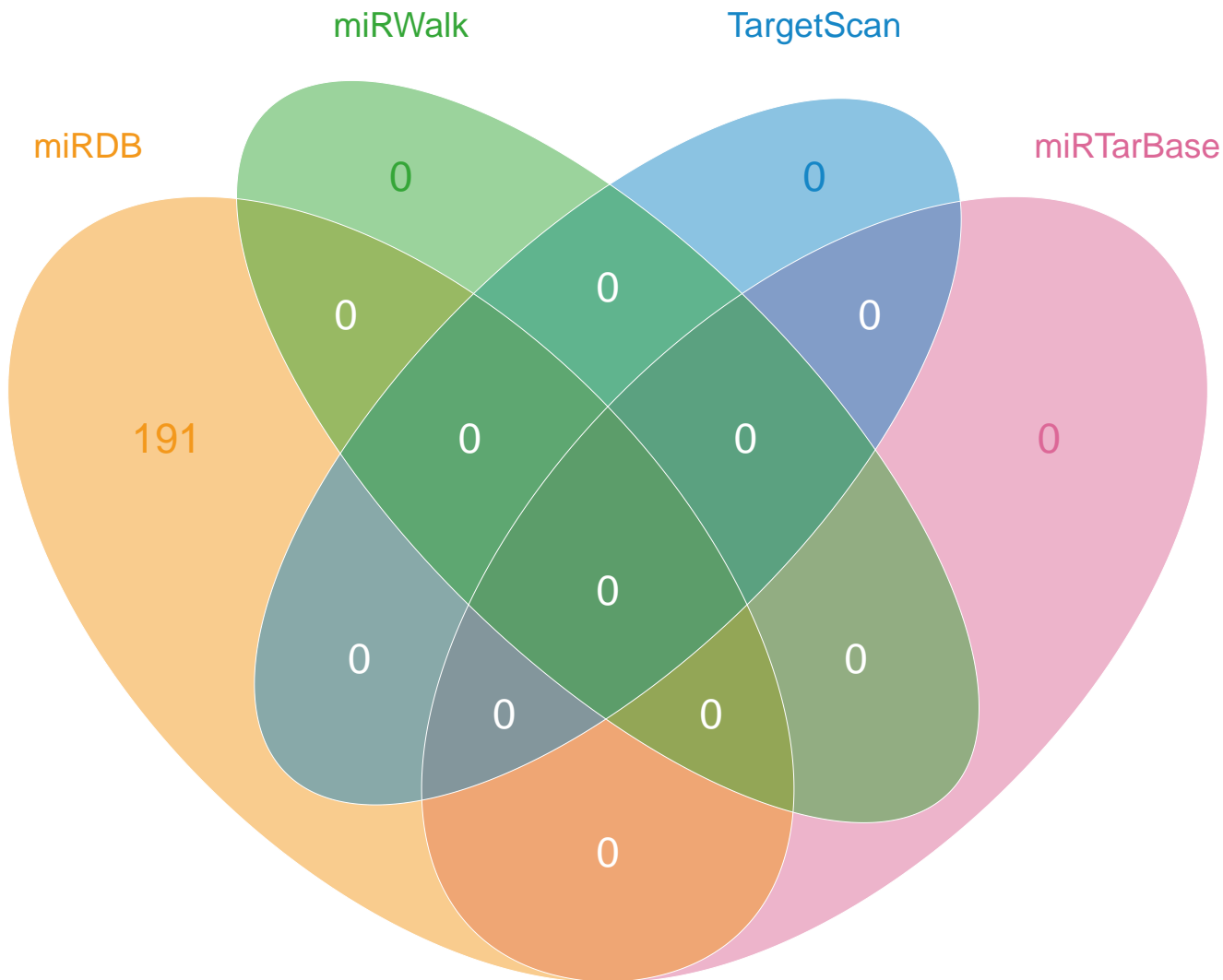

Supplement: Supplementary file 1 [file Presentation1.zip › Data/microRN/miR_target/Control--Treatment/result/rno-miR-3084a-1-5p/rno-miR-3084a-1-5p.pdf]

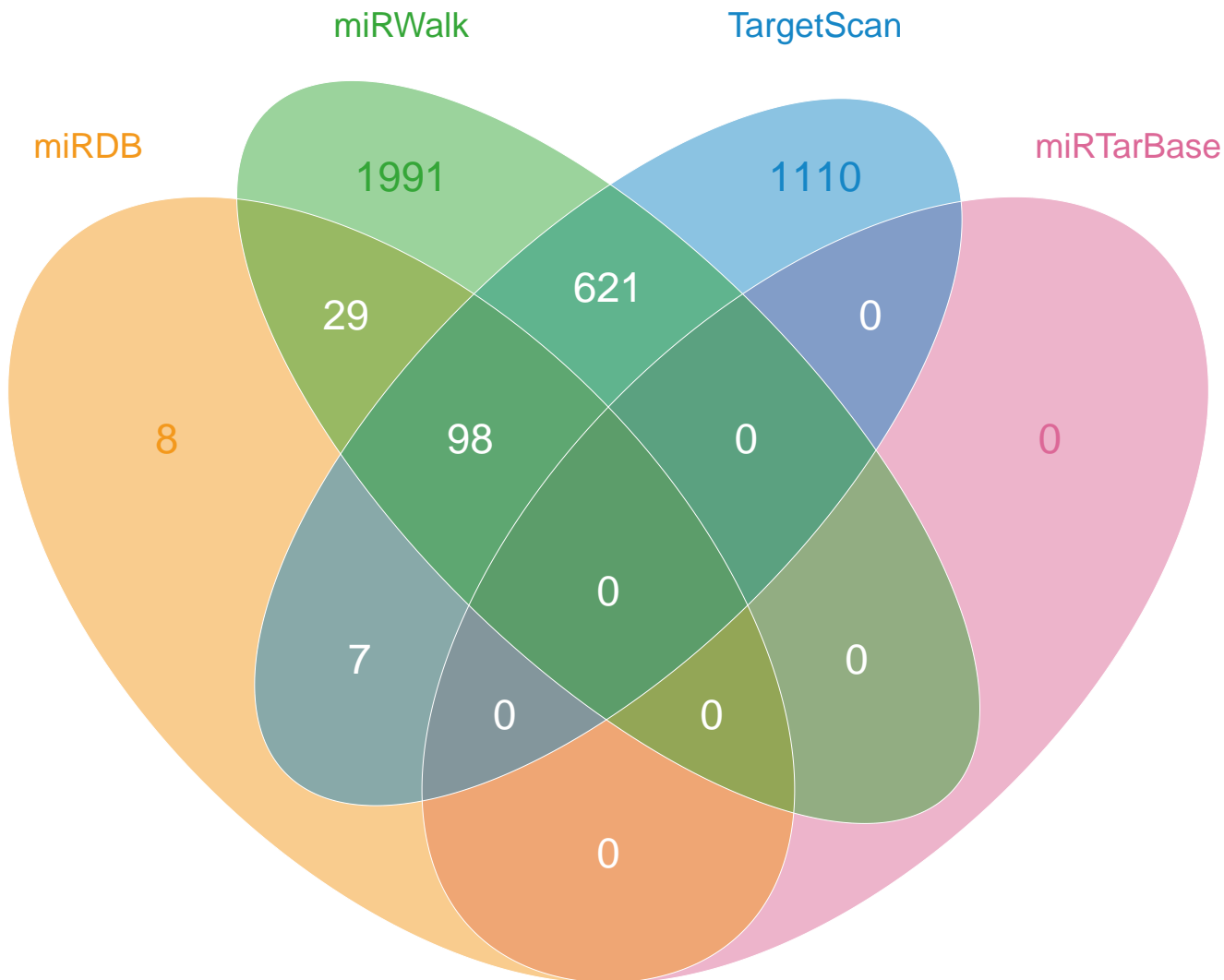

Supplement: Supplementary file 1 [file Presentation1.zip › Data/microRN/miR_target/Control--Treatment/result/rno-miR-380-3p/rno-miR-380-3p.pdf]

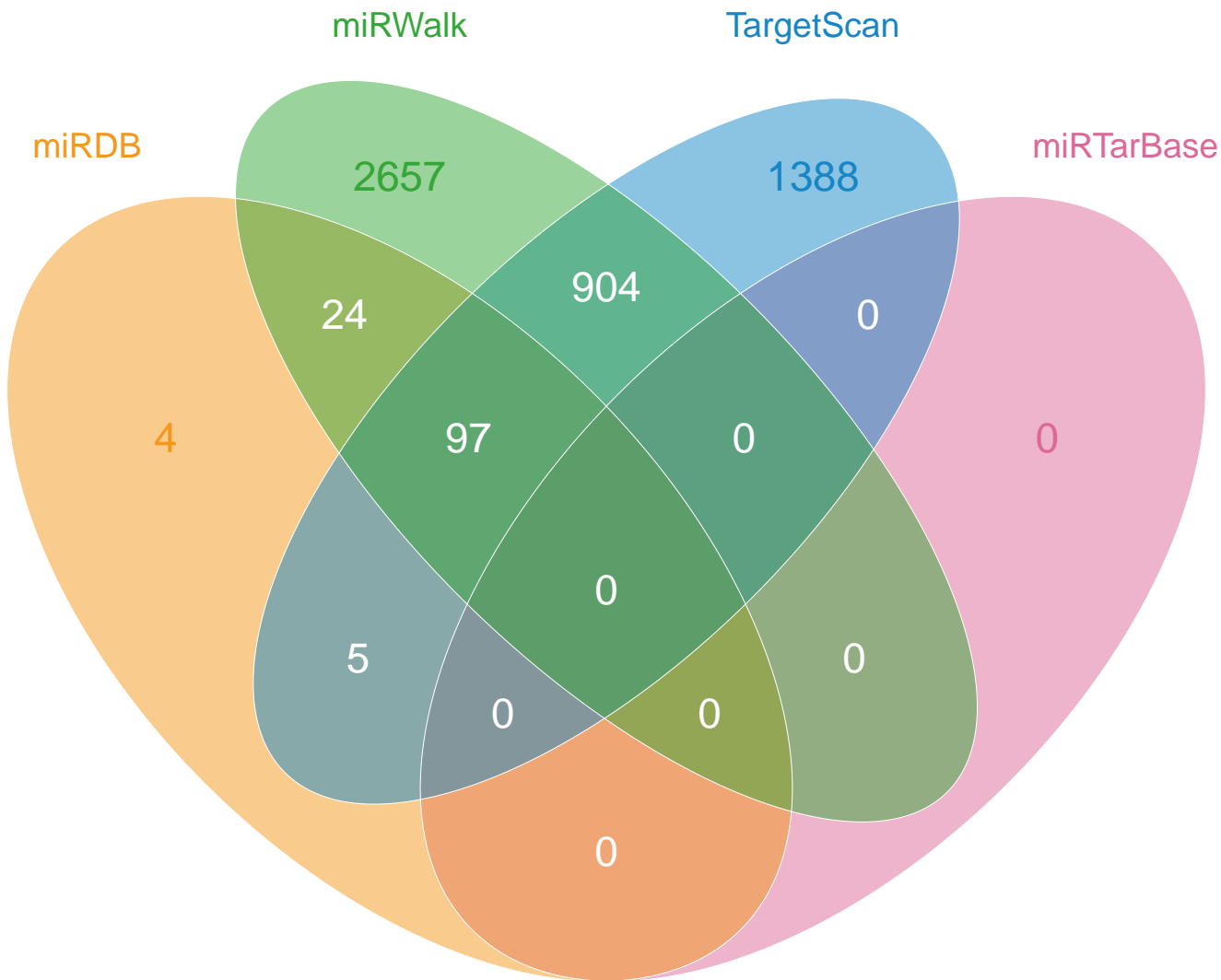

Supplement: Supplementary file 1 [file Presentation1.zip › Data/microRN/miR_target/Control--Treatment/result/rno-miR-490-5p/rno-miR-490-5p.pdf]

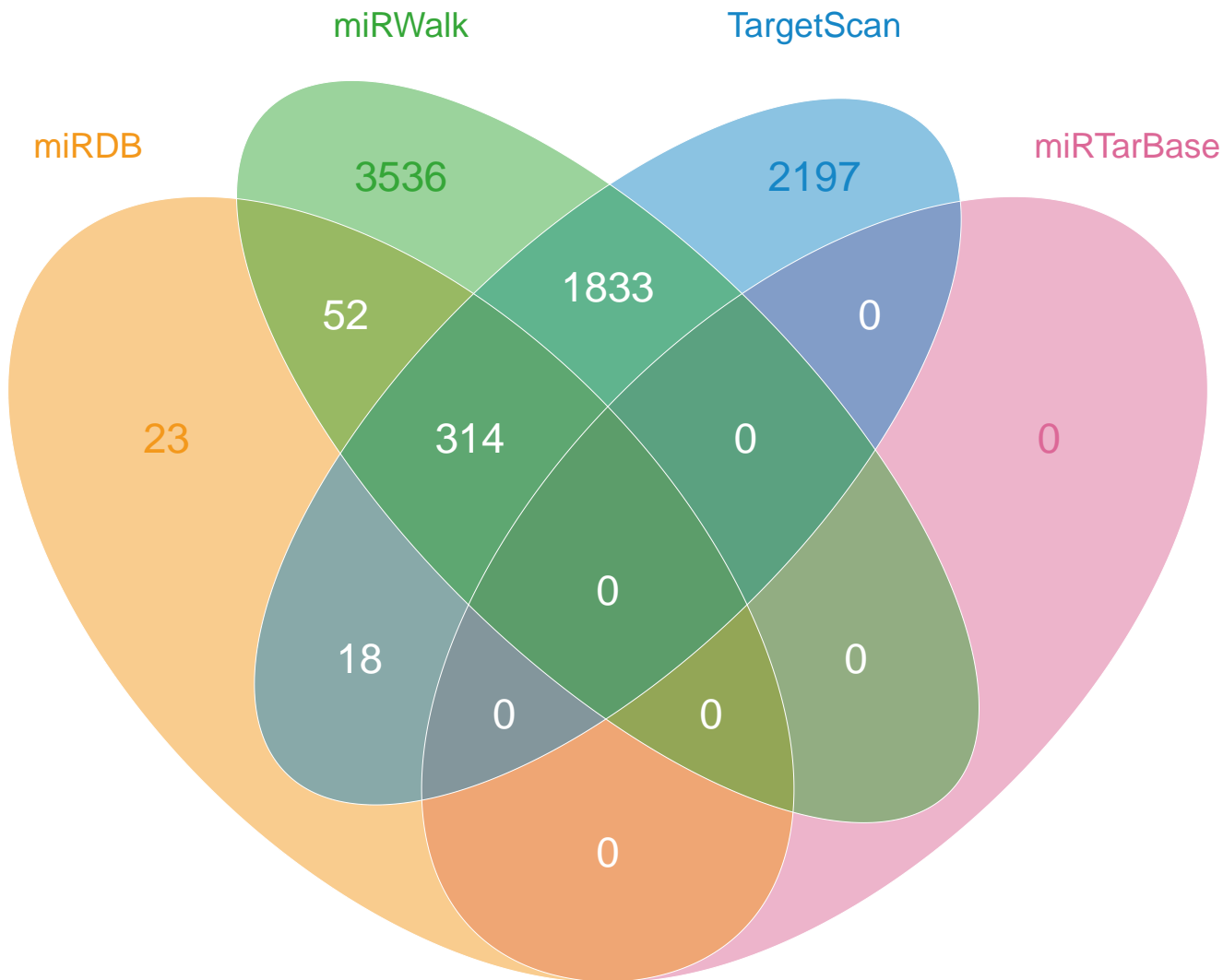

Supplement: Supplementary file 1 [file Presentation1.zip › Data/microRN/miR_target/Control--Treatment/result/rno-miR-539-5p/rno-miR-539-5p.pdf]

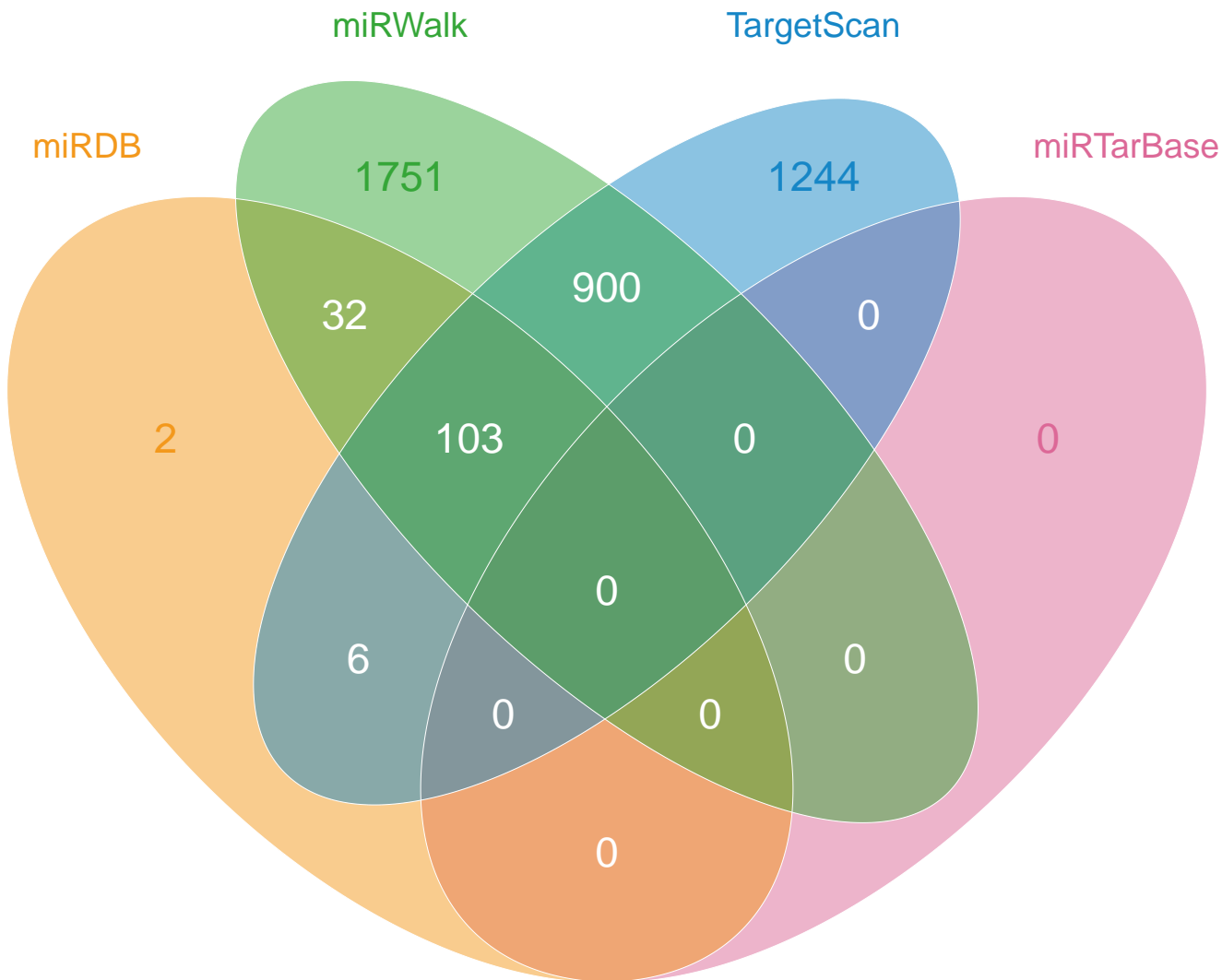

Supplement: Supplementary file 1 [file Presentation1.zip › Data/microRN/miR_target/Control--Treatment/result/rno-miR-667-3p/rno-miR-667-3p.pdf]

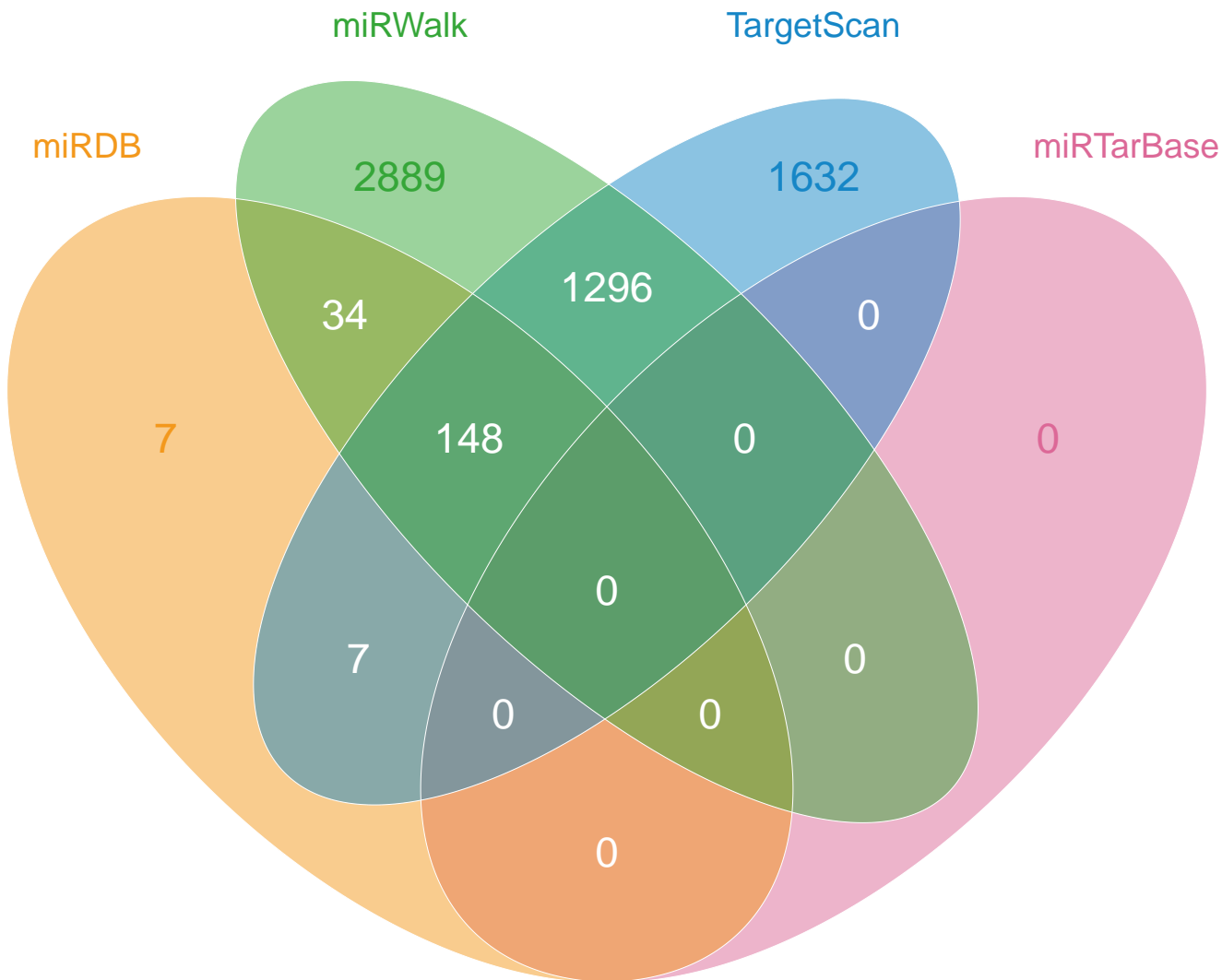

Supplement: Supplementary file 1 [file Presentation1.zip › Data/microRN/miR_target/Control--Treatment/result/rno-miR-879-5p/rno-miR-879-5p.pdf]

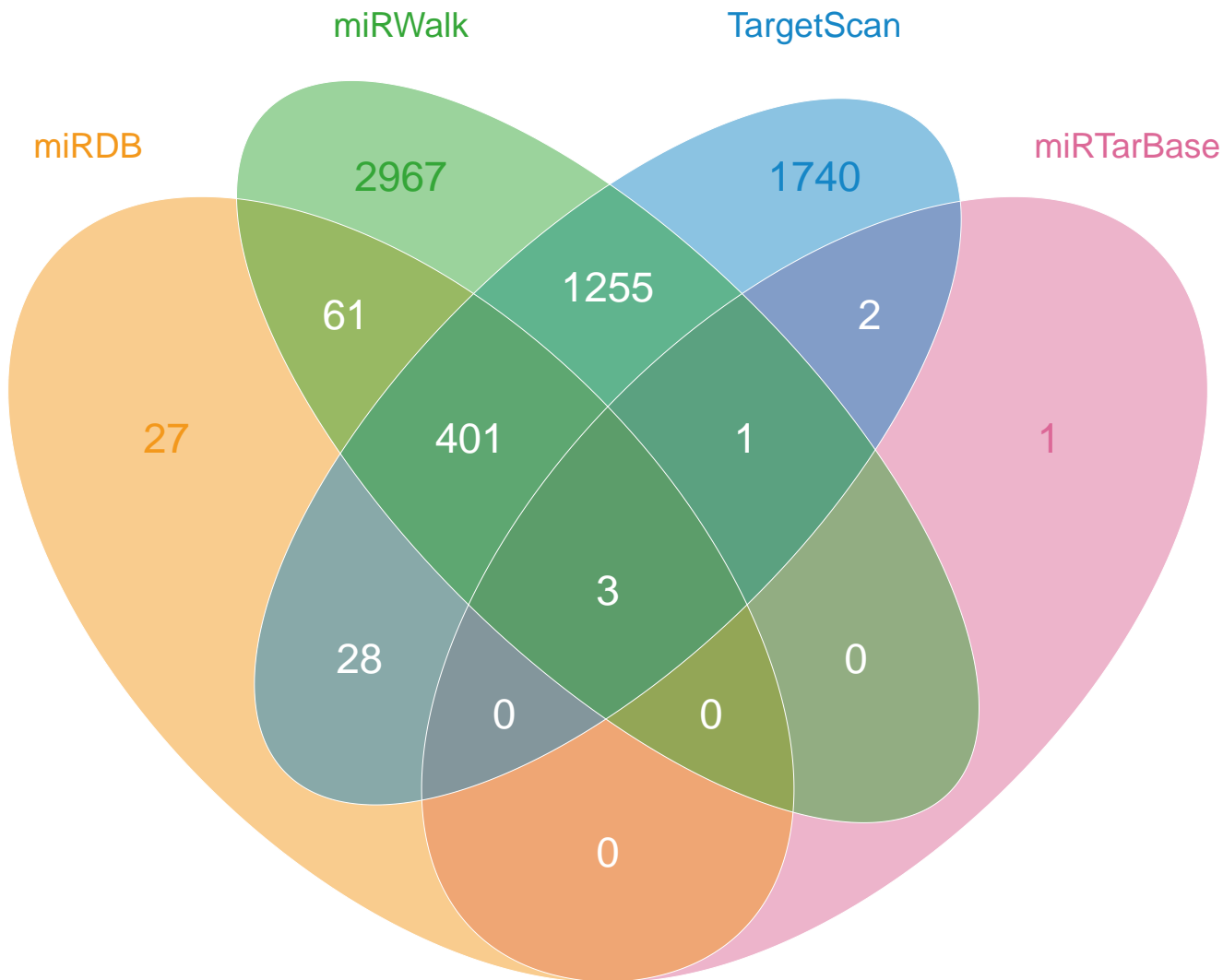

Supplement: Supplementary file 1 [file Presentation1.zip › Data/microRN/miR_target/Control--Treatment/result/rno-miR-9a-5p/rno-miR-9a-5p.pdf]

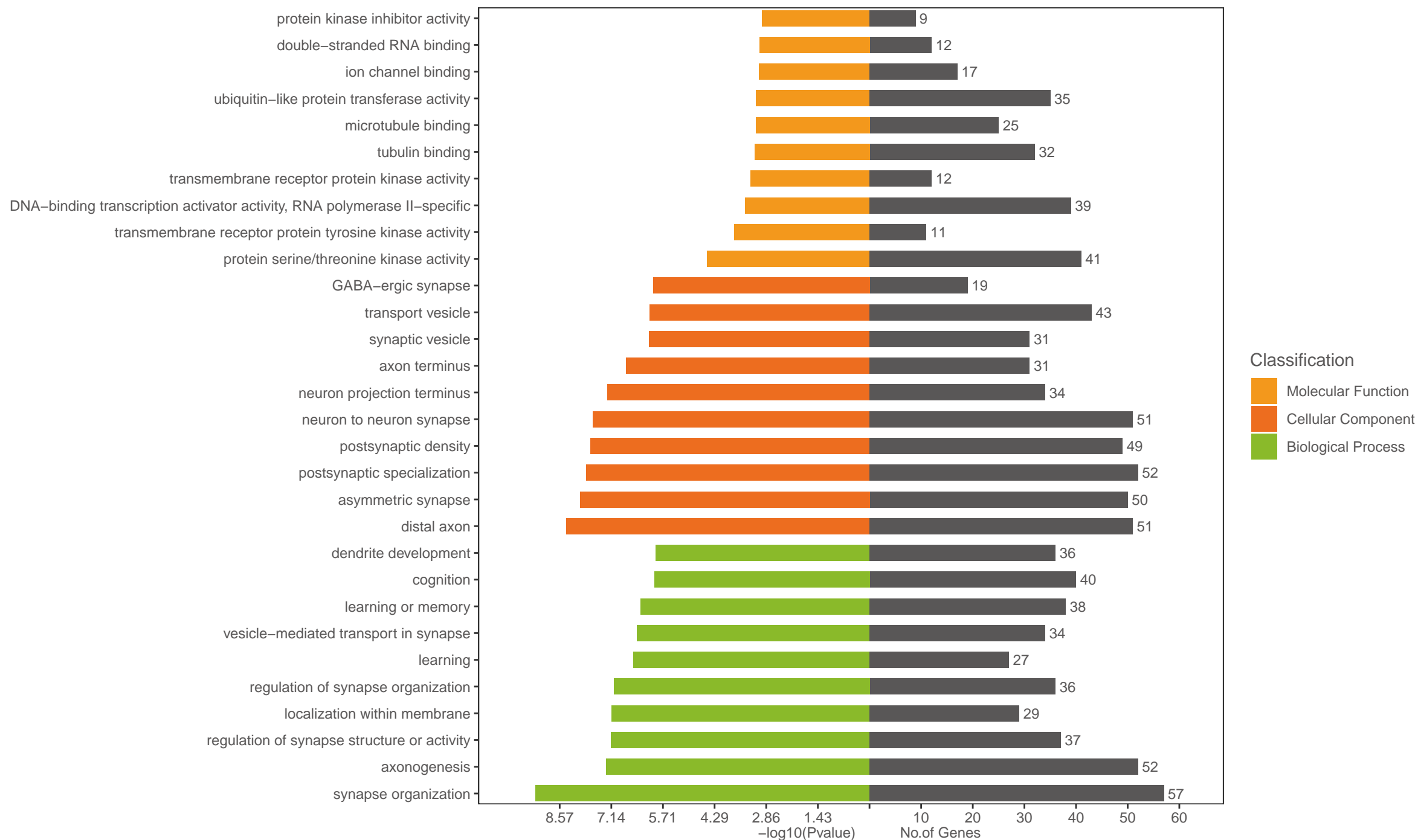

Supplement: Supplementary file 1 [file Presentation1.zip › Data/miRNA/GO/Control--Treatment/go.pdf]

Statistics of Pathway Enrichment

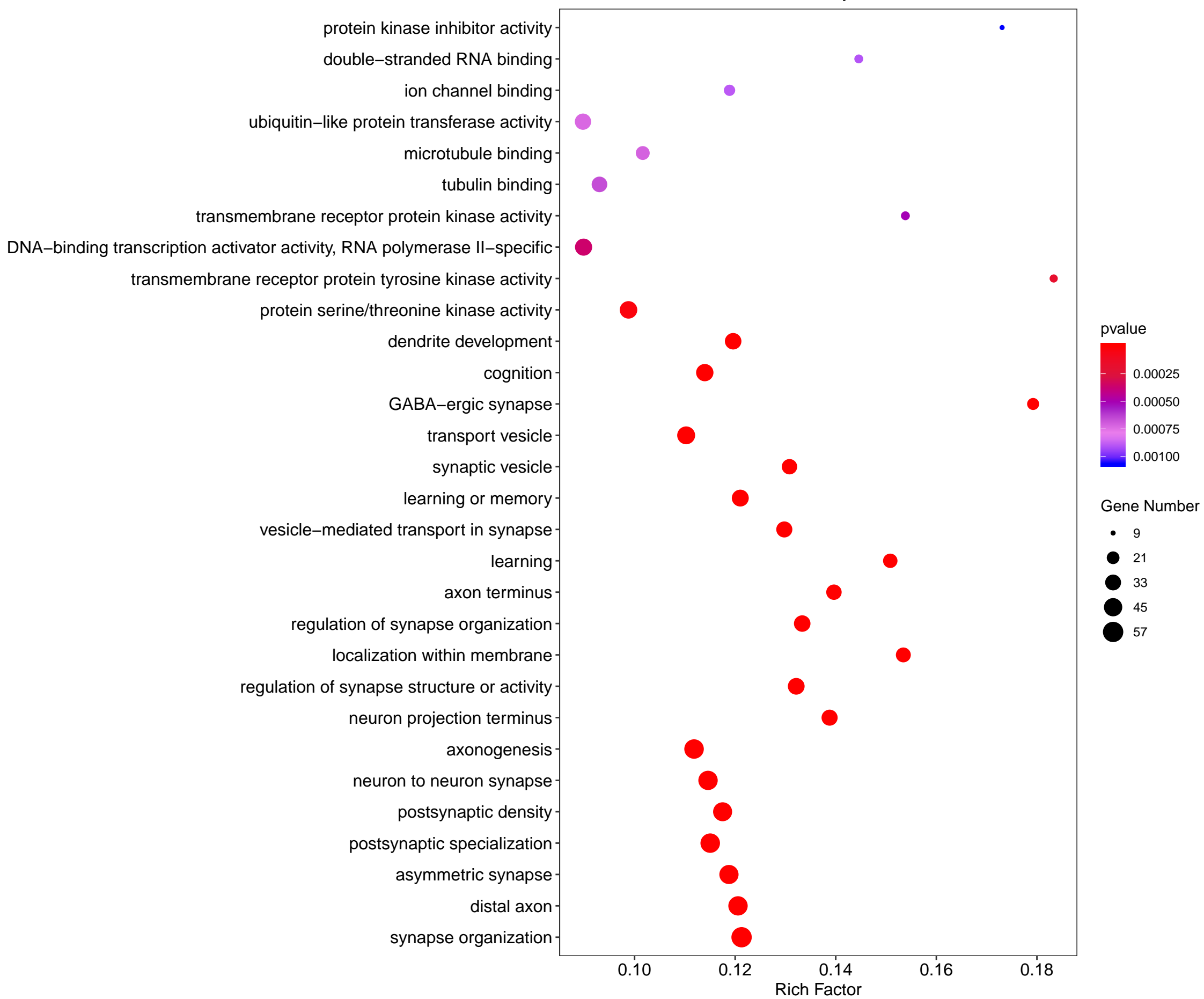

Supplement: Supplementary file 1 [file Presentation1.zip › Data/miRNA/GO/Control--Treatment/go.point.pdf]

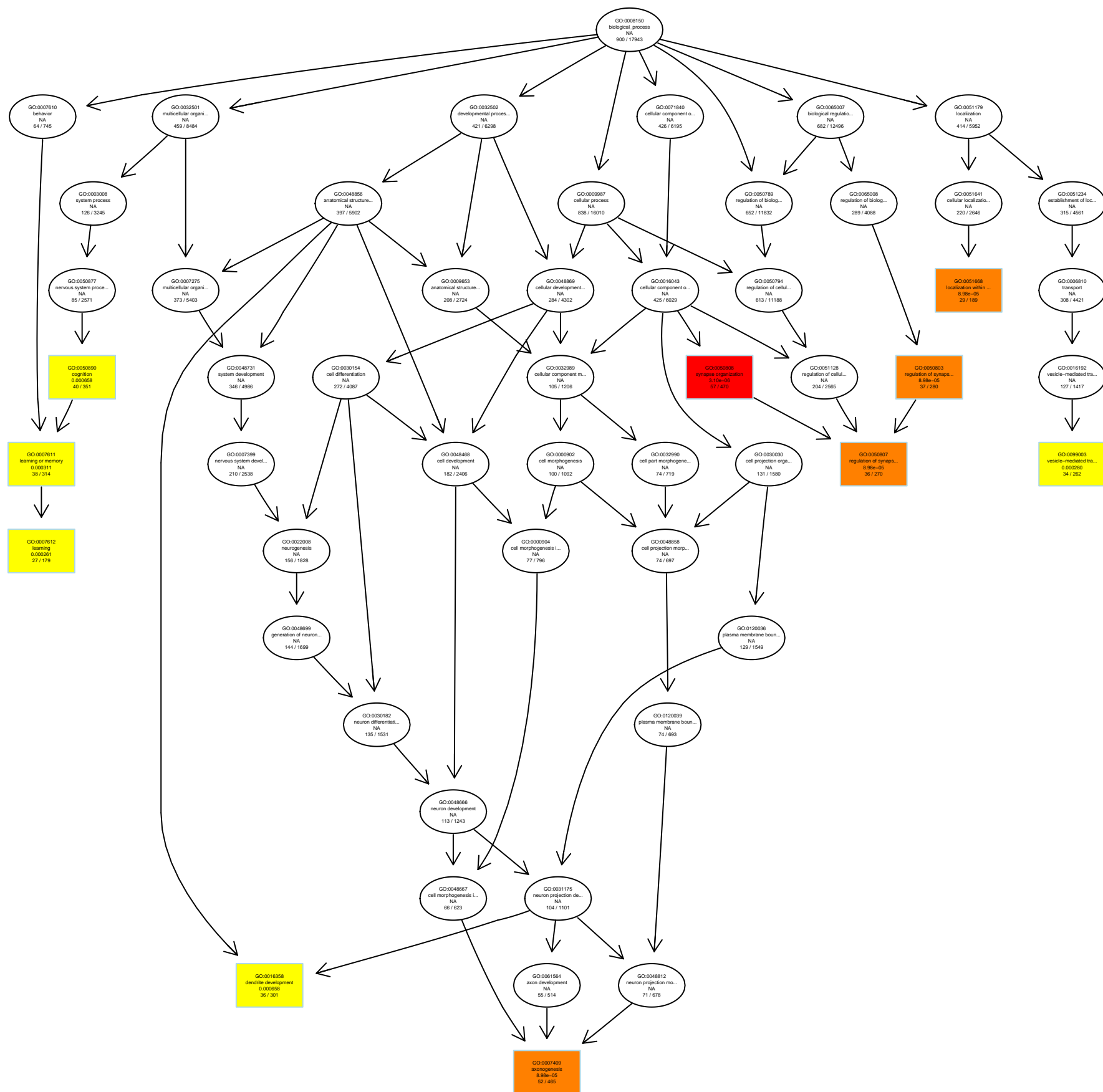

Supplement: Supplementary file 1 [file Presentation1.zip › Data/miRNA/GO/Control--Treatment/GO-Biological_Process.pdf]

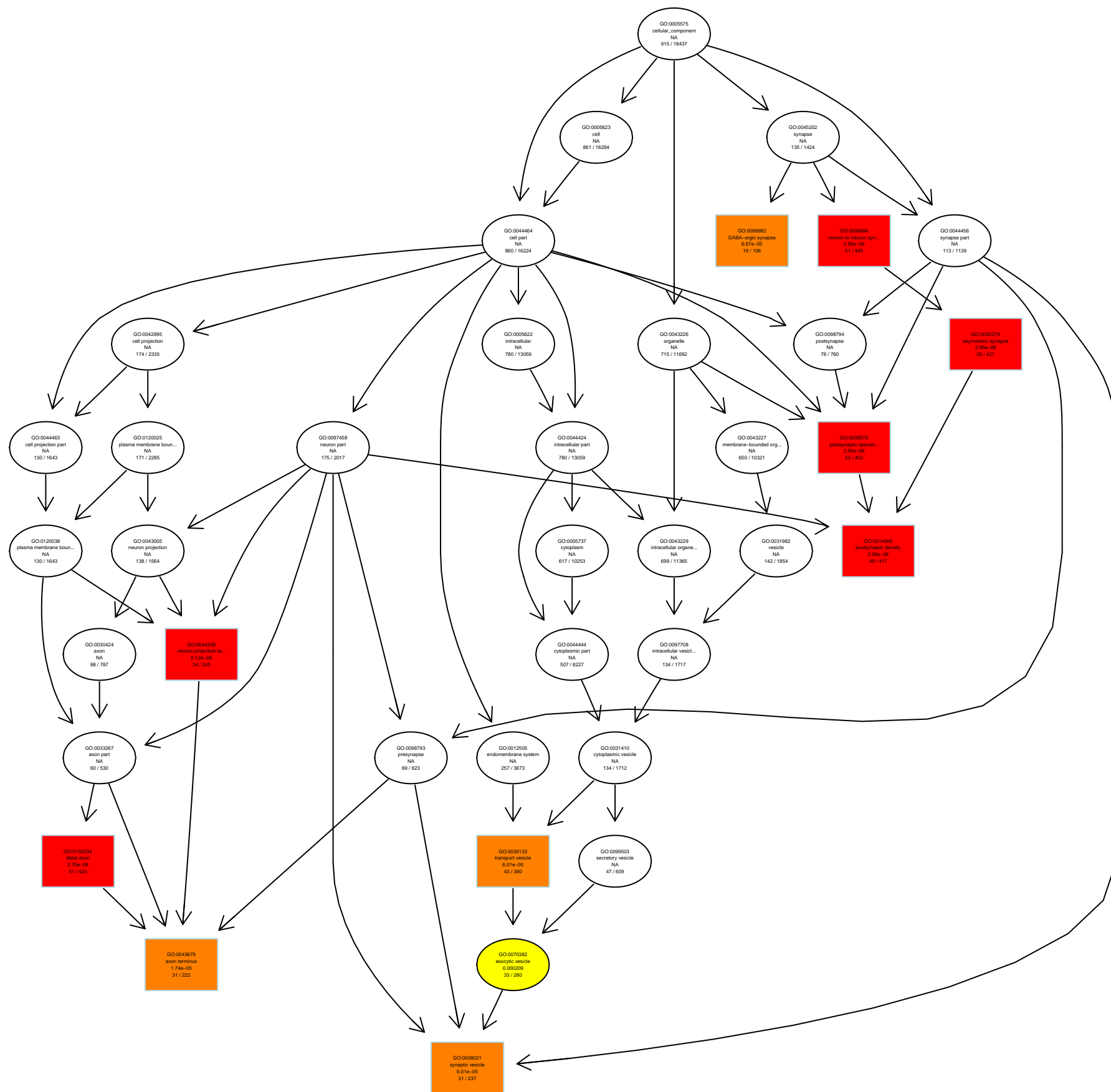

Supplement: Supplementary file 1 [file Presentation1.zip › Data/miRNA/GO/Control--Treatment/GO-Cellular_Component.pdf]

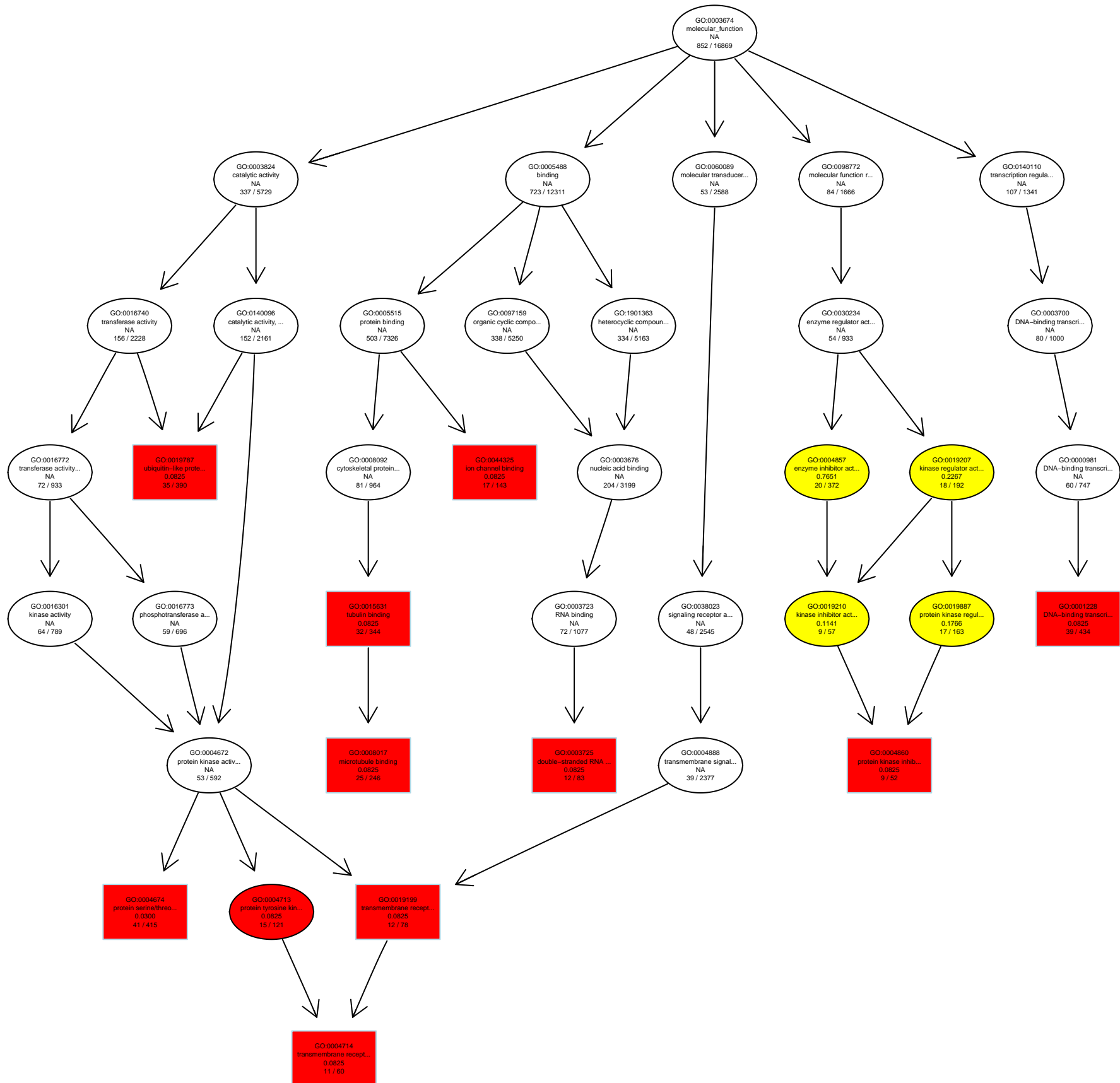

Supplement: Supplementary file 1 [file Presentation1.zip › Data/miRNA/GO/Control--Treatment/GO-Molecular_Function.pdf]

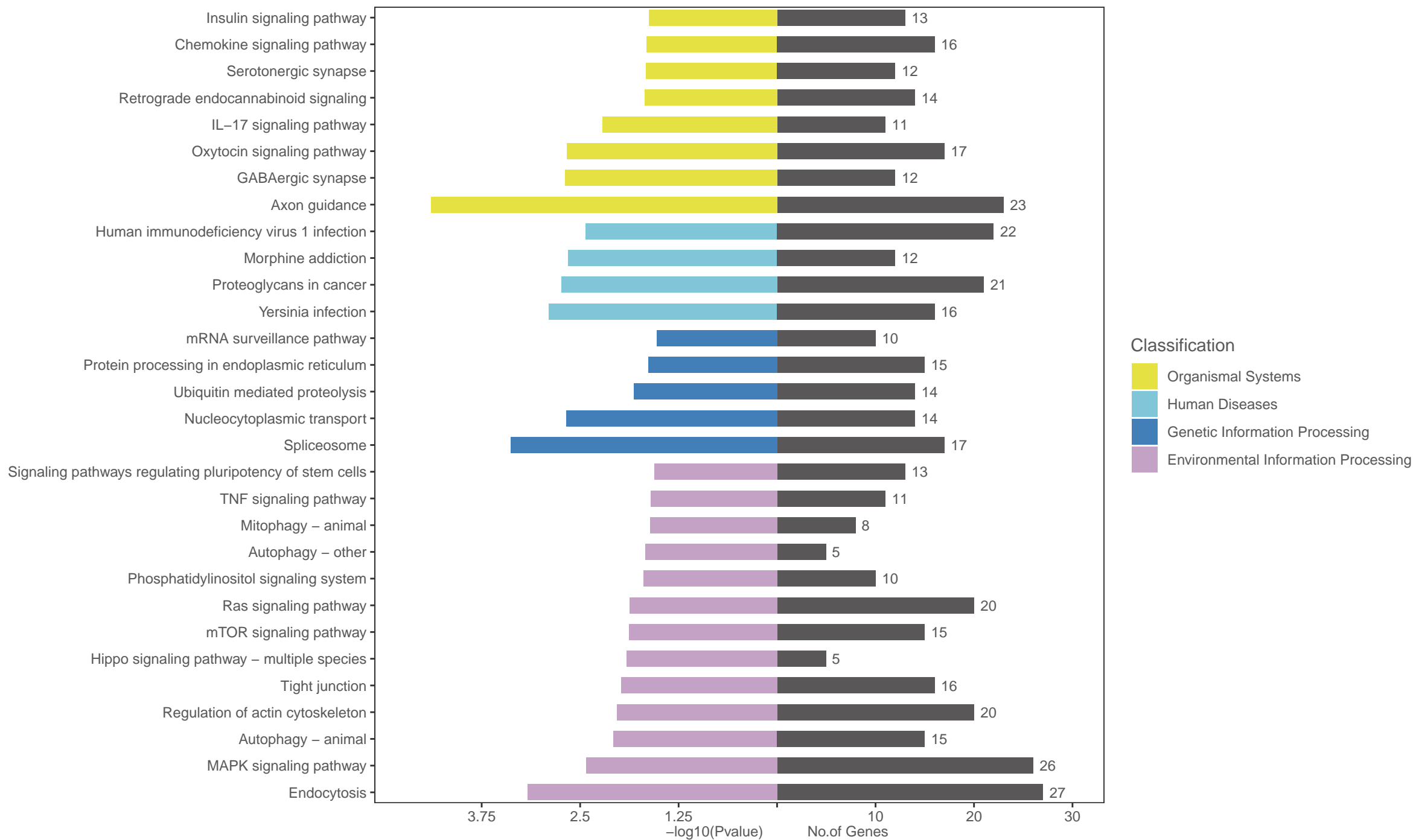

Supplement: Supplementary file 1 [file Presentation1.zip › Data/miRNA/KEGG/Control--Treatment/kegg.pdf]

Statistics of Pathway Enrichment

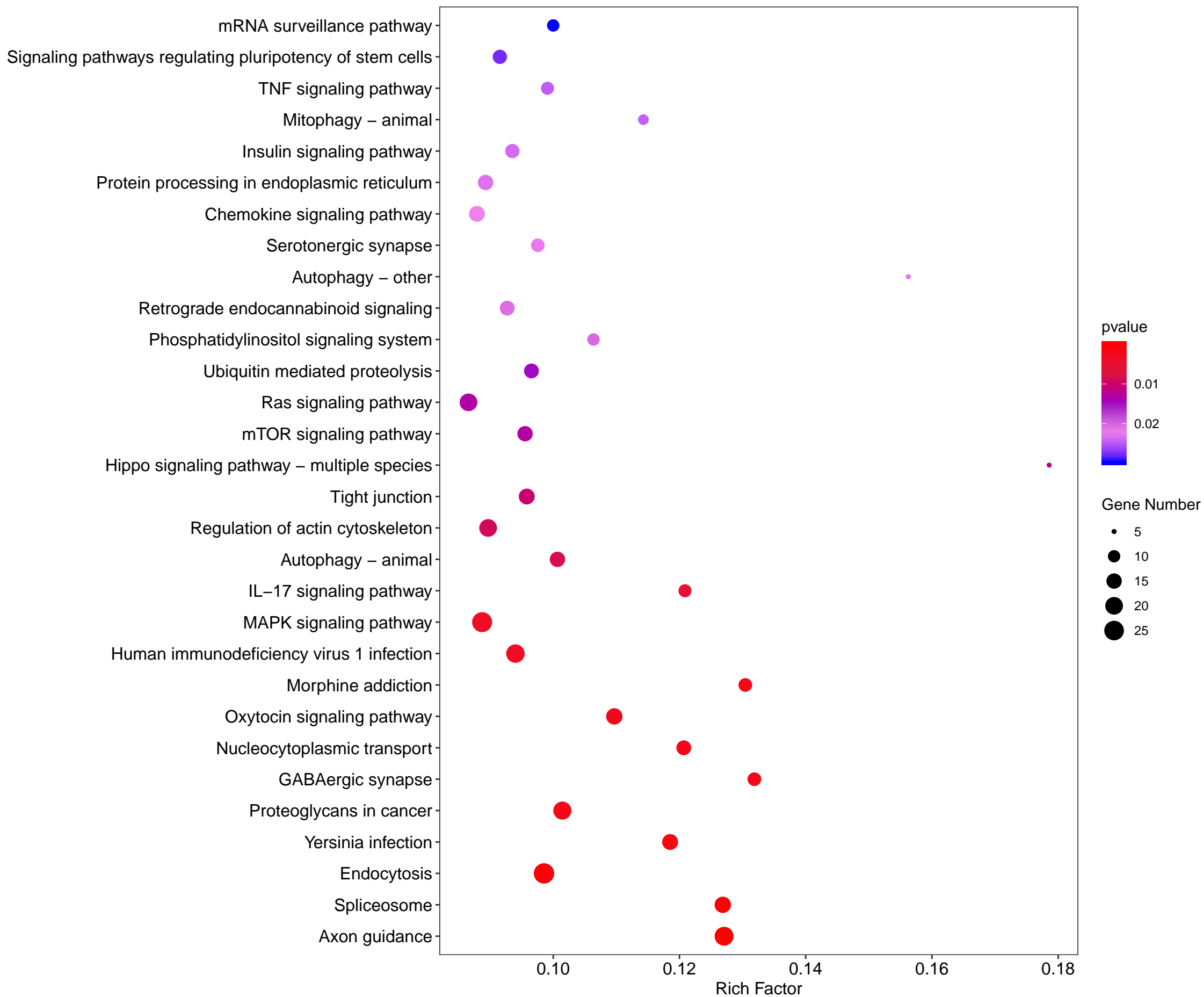

Supplement: Supplementary file 1 [file Presentation1.zip › Data/miRNA/KEGG/Control--Treatment/kegg.point.pdf]

# sRNA nucleotide bias at each position

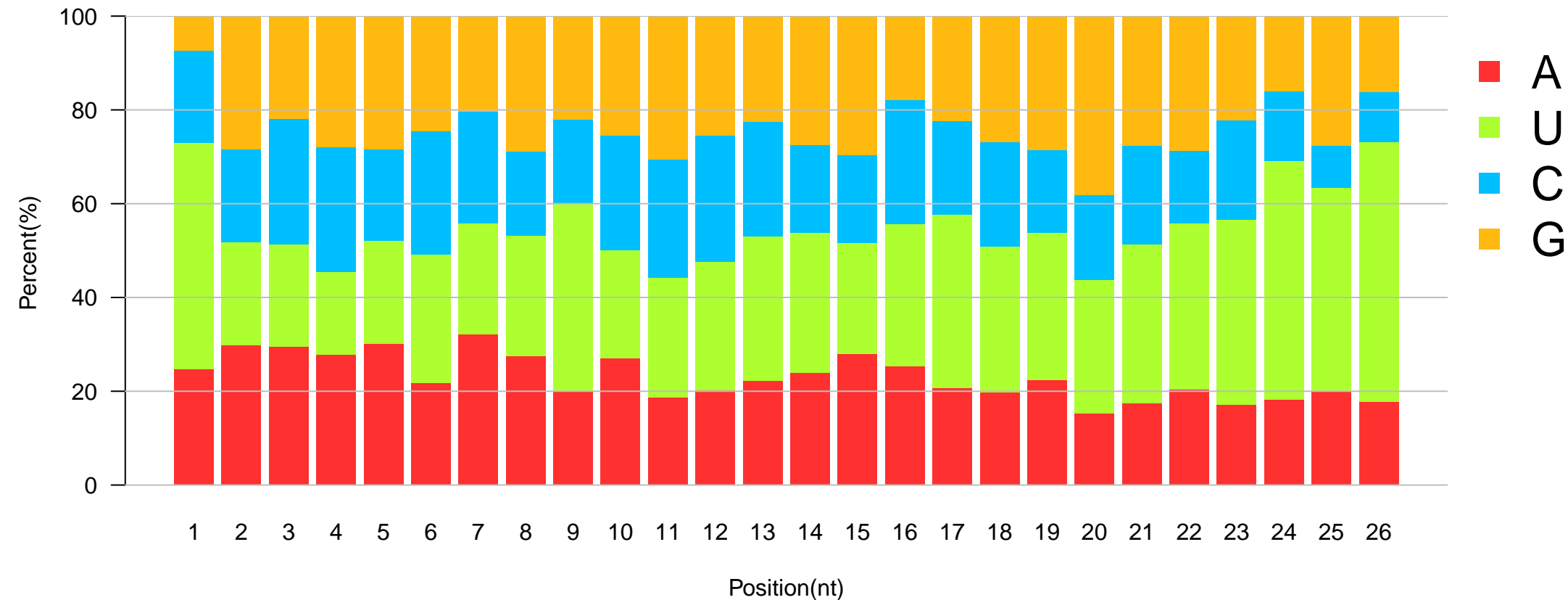

Supplement: Supplementary file 1 [file Presentation1.zip › Data/miRNA/know_miRNA_expression/1/1_base_preference.pdf]

**Read Length Distribution**

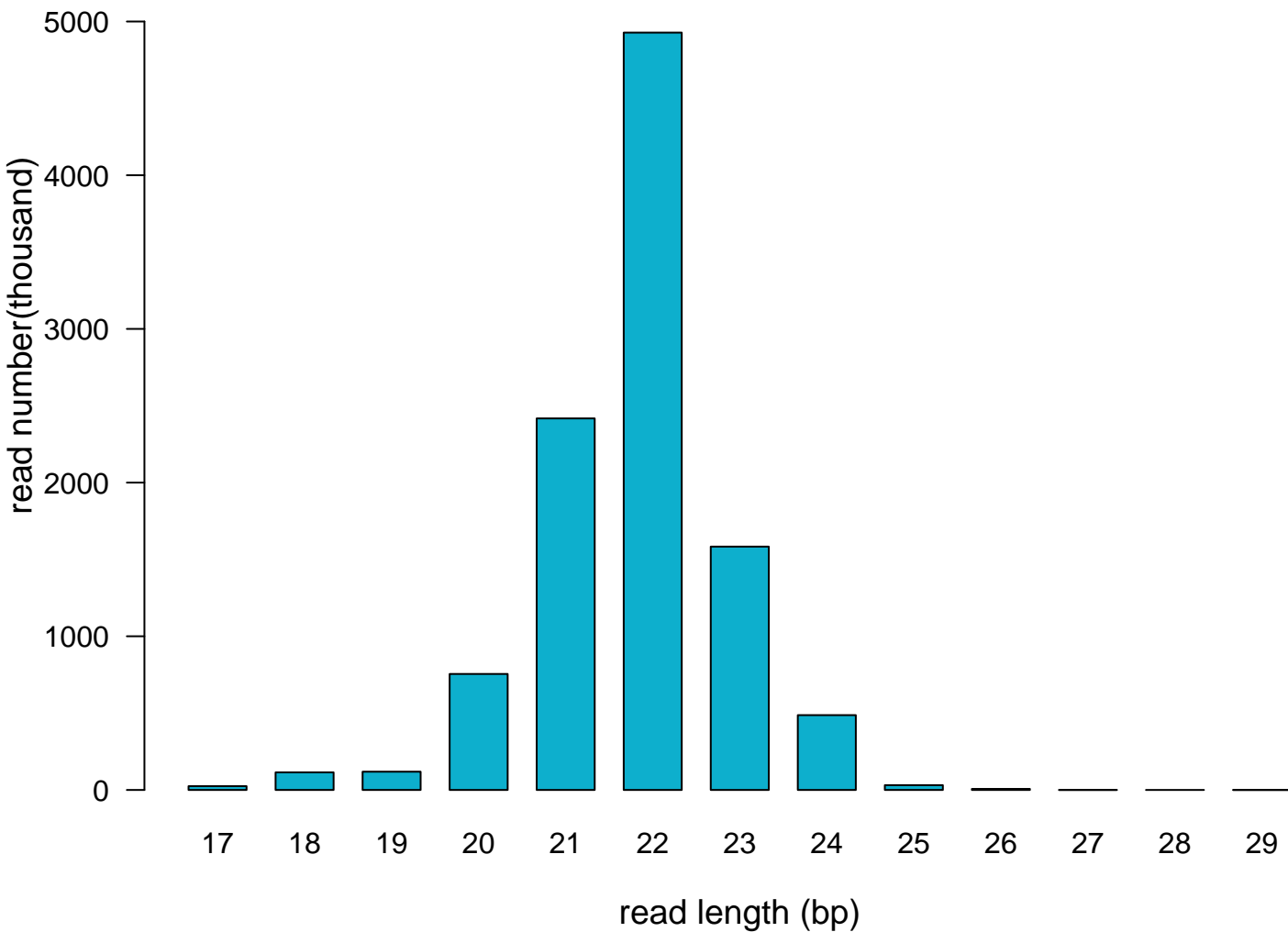

Supplement: Supplementary file 1 [file Presentation1.zip › Data/miRNA/know_miRNA_expression/1/1_miRNA_length.pdf]

# sRNA nucleotide bias at each position

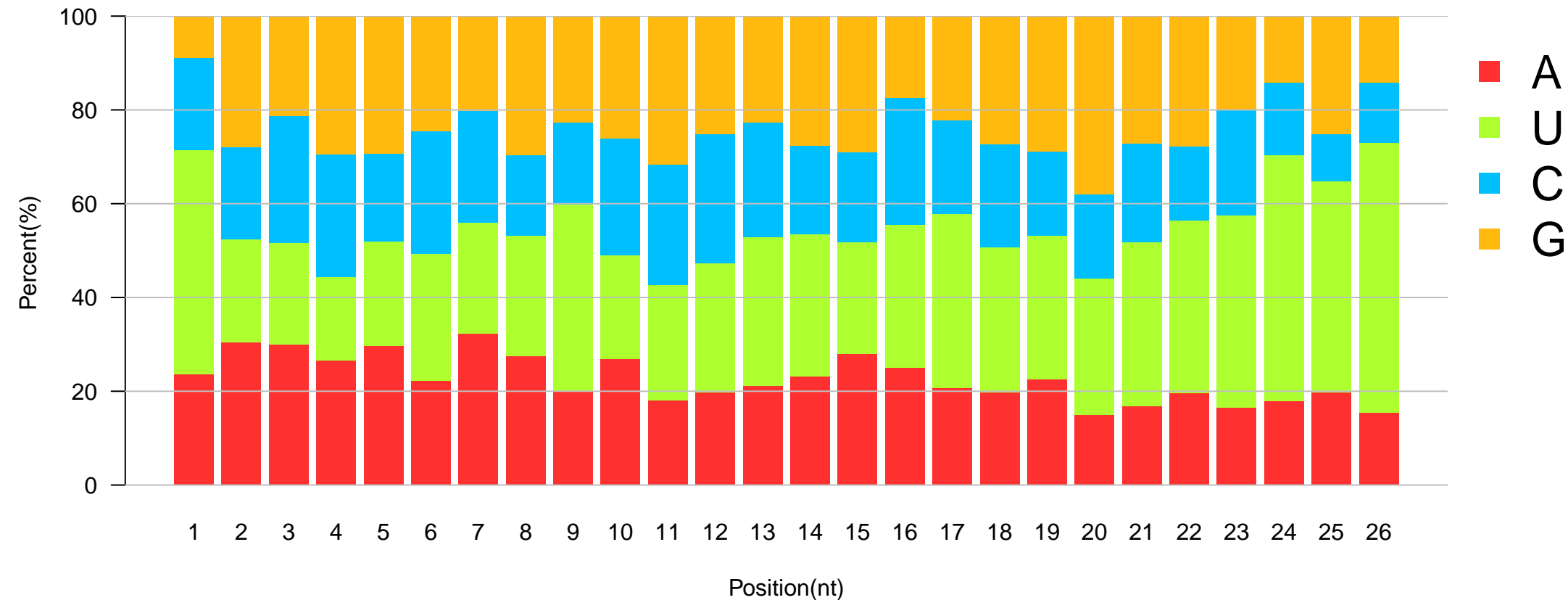

Supplement: Supplementary file 1 [file Presentation1.zip › Data/miRNA/know_miRNA_expression/2/2_base_preference.pdf]

**Read Length Distribution**

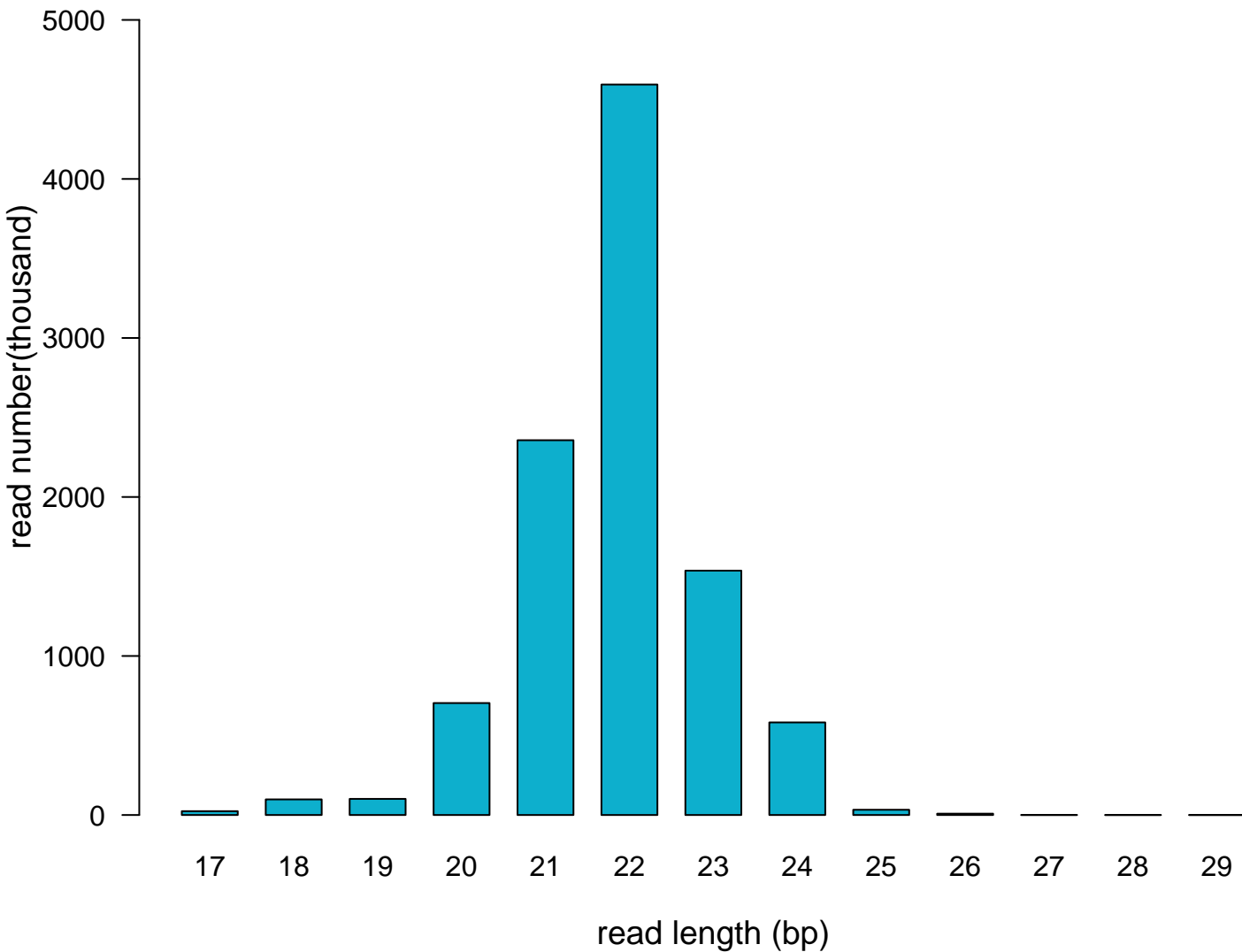

Supplement: Supplementary file 1 [file Presentation1.zip › Data/miRNA/know_miRNA_expression/2/2_miRNA_length.pdf]

# sRNA nucleotide bias at each position

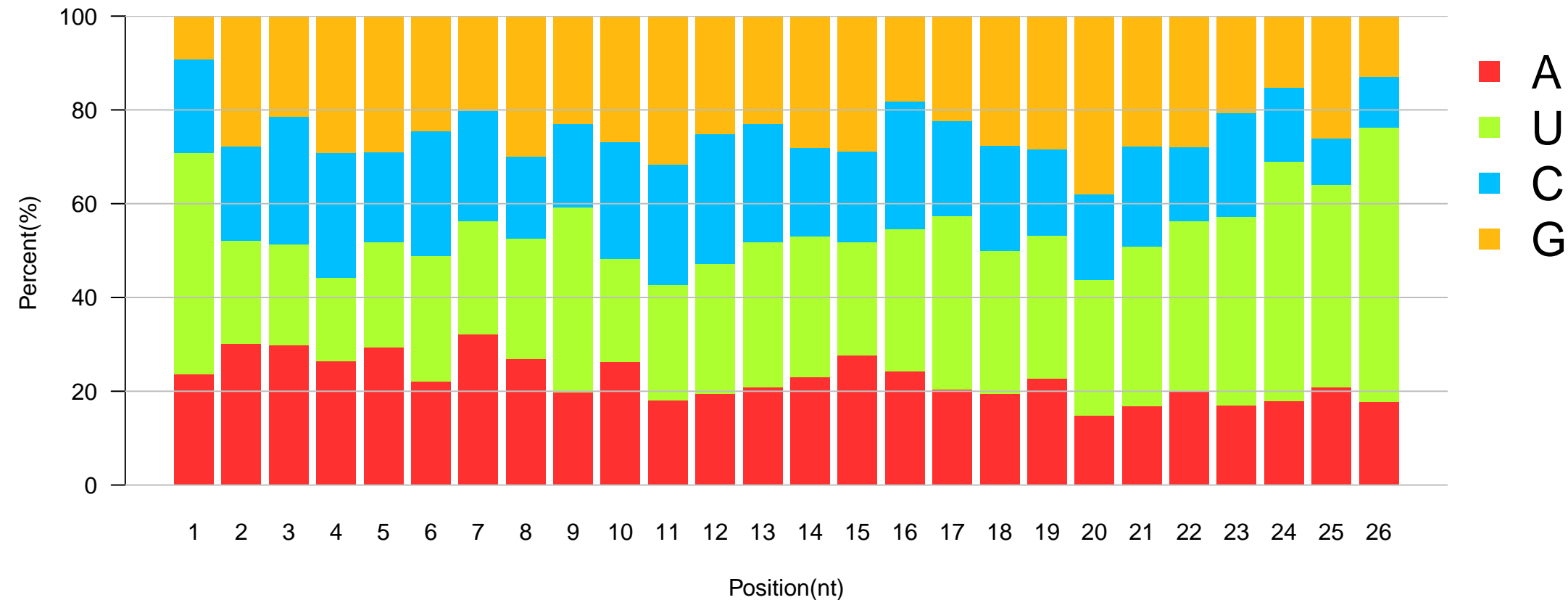

Supplement: Supplementary file 1 [file Presentation1.zip › Data/miRNA/know_miRNA_expression/3/3_base_preference.pdf]

**Read Length Distribution**

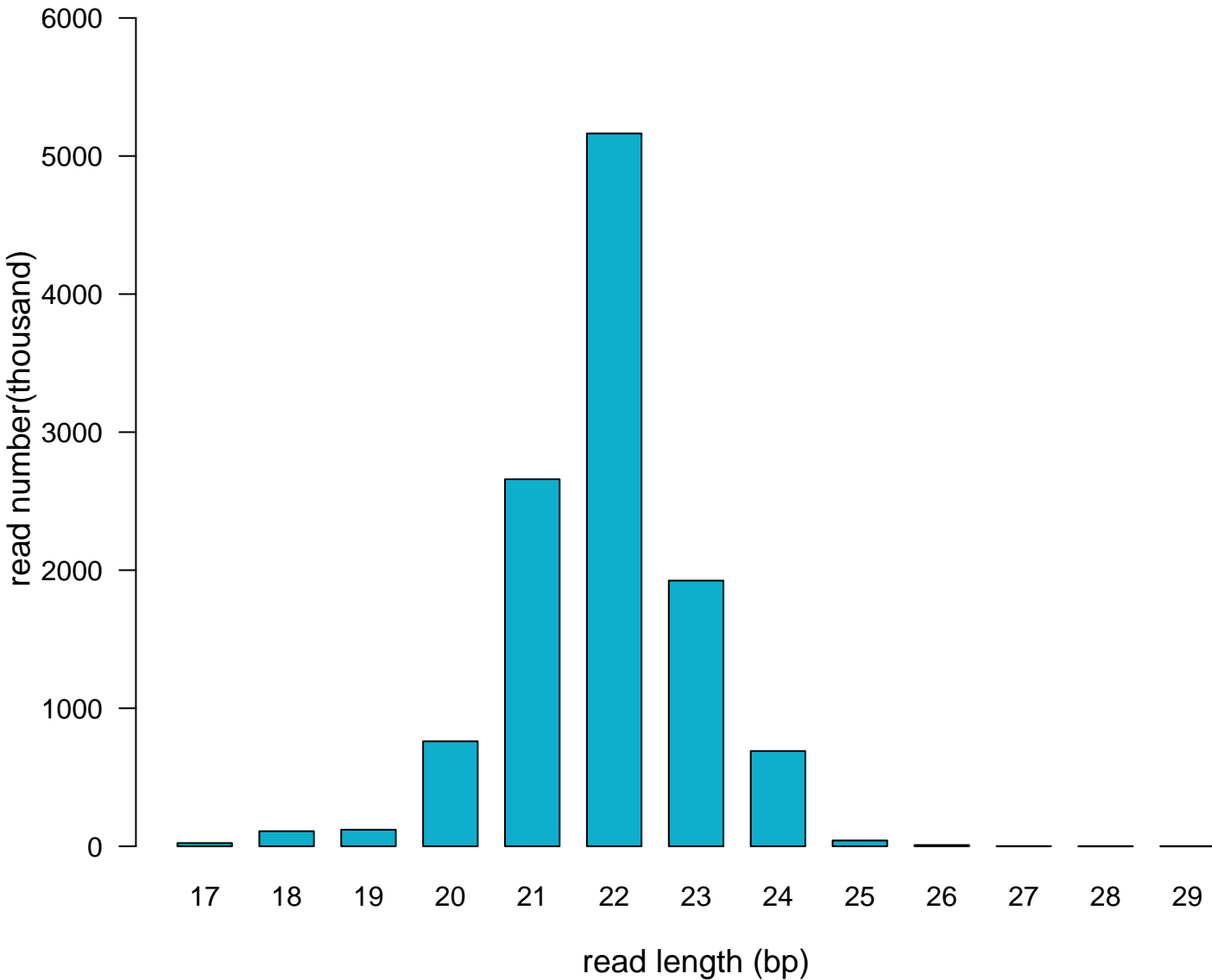

Supplement: Supplementary file 1 [file Presentation1.zip › Data/miRNA/know_miRNA_expression/3/3_miRNA_length.pdf]

# sRNA nucleotide bias at each position

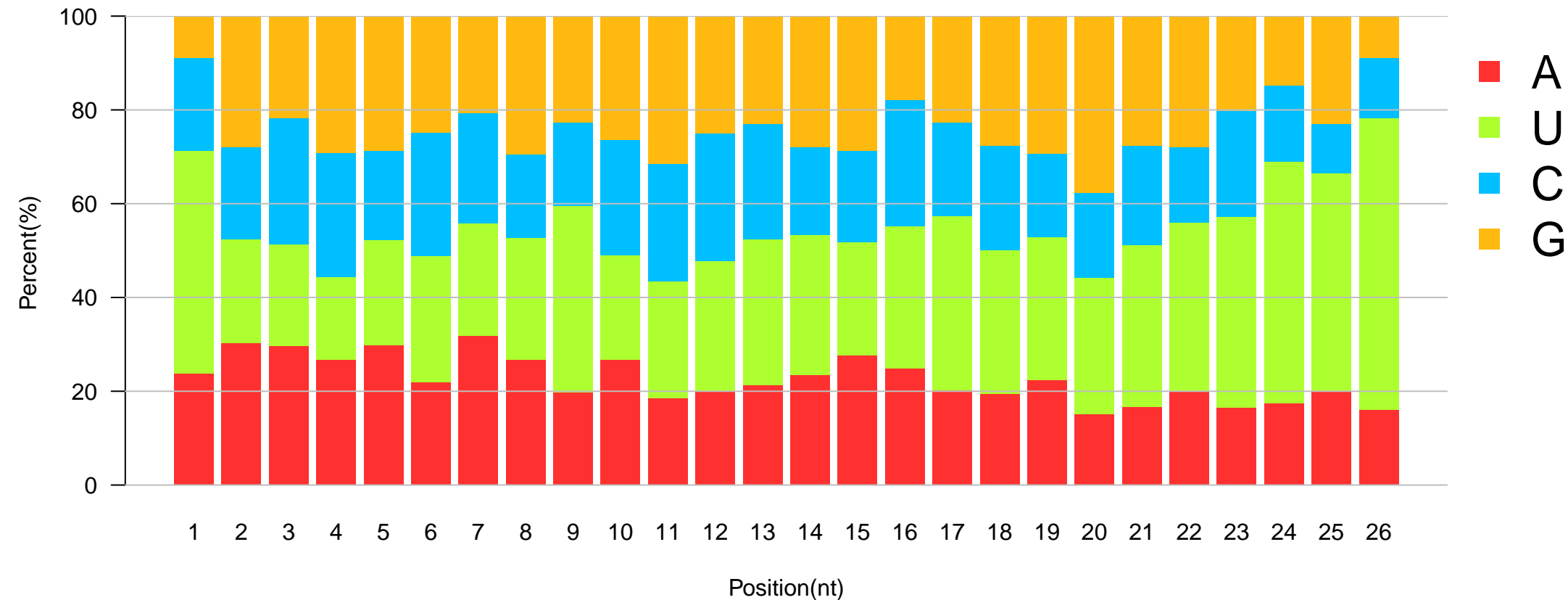

Supplement: Supplementary file 1 [file Presentation1.zip › Data/miRNA/know_miRNA_expression/4/4_base_preference.pdf]

# Read Length Distribution

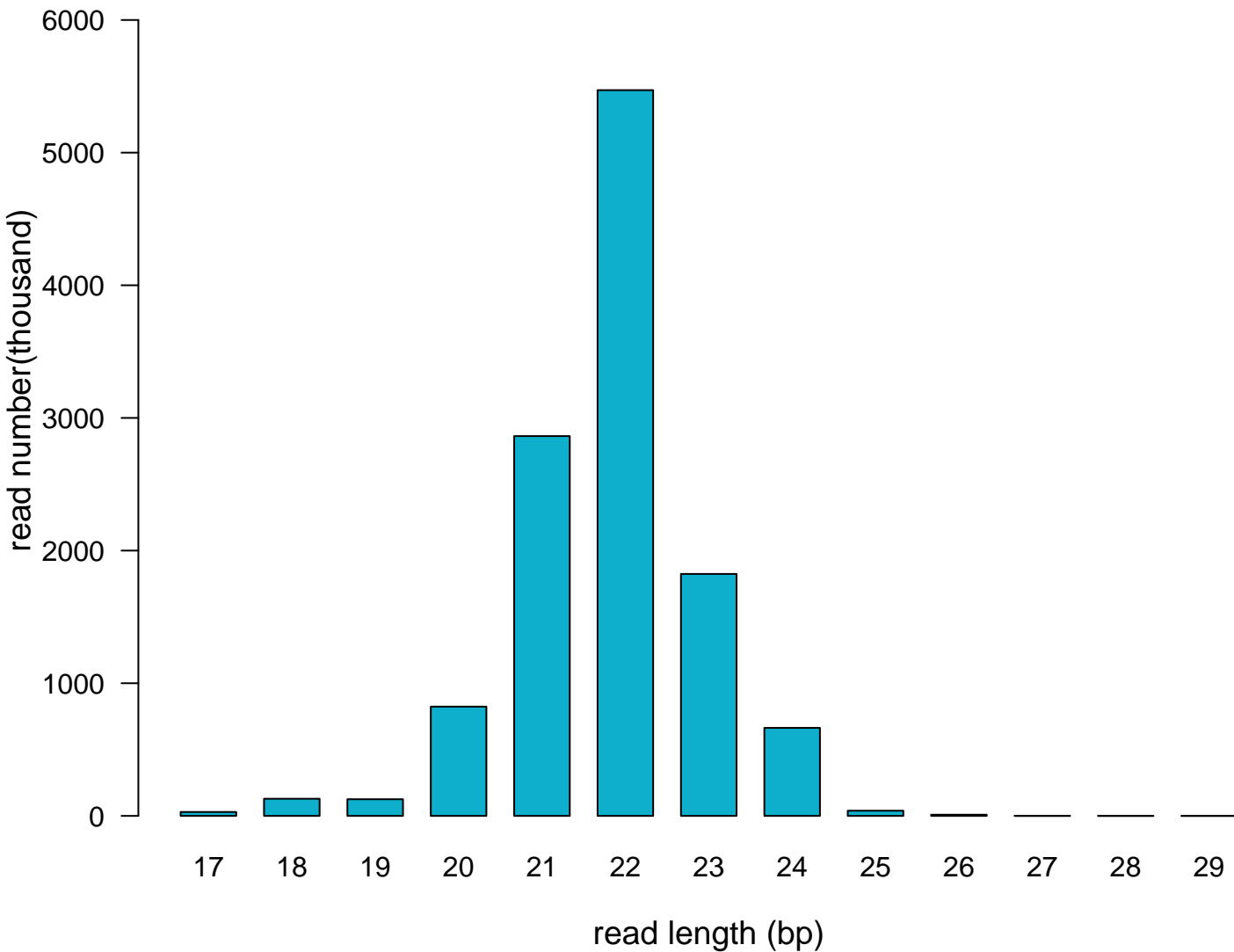

Supplement: Supplementary file 1 [file Presentation1.zip › Data/miRNA/know_miRNA_expression/4/4_miRNA_length.pdf]

# sRNA nucleotide bias at each position

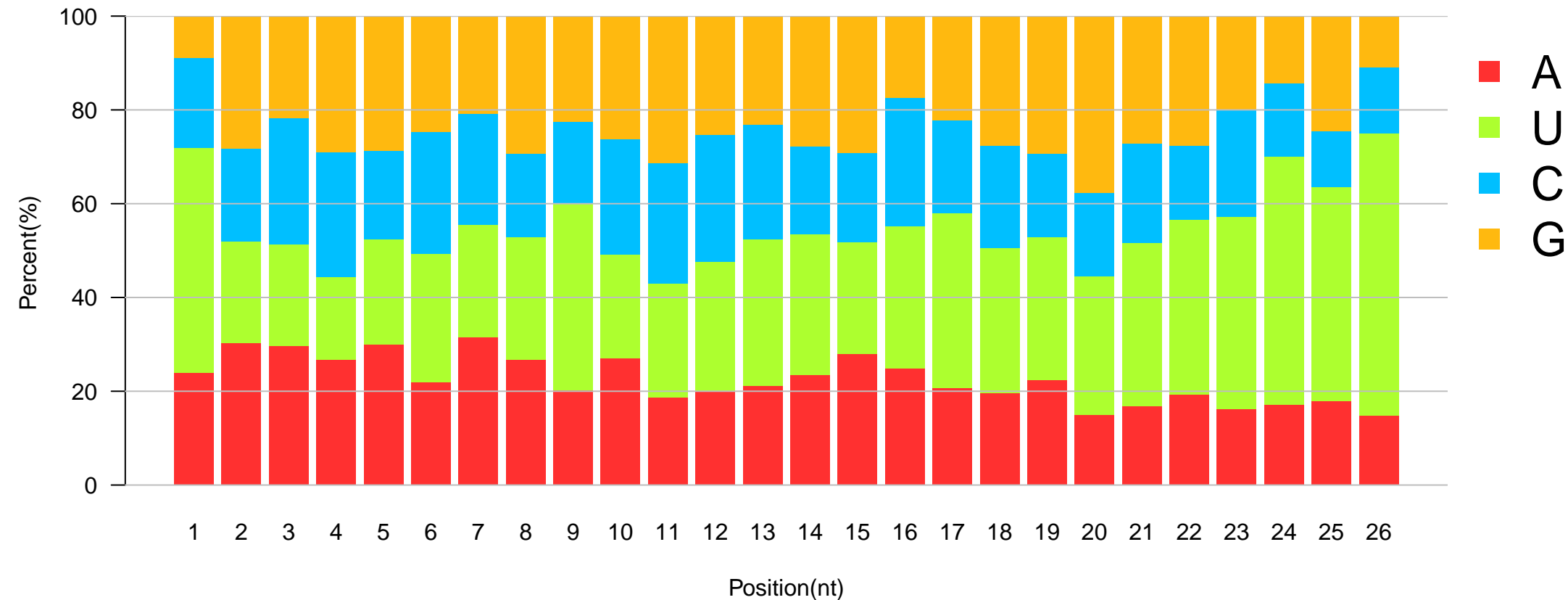

Supplement: Supplementary file 1 [file Presentation1.zip › Data/miRNA/know_miRNA_expression/5/5_base_preference.pdf]

**Read Length Distribution**

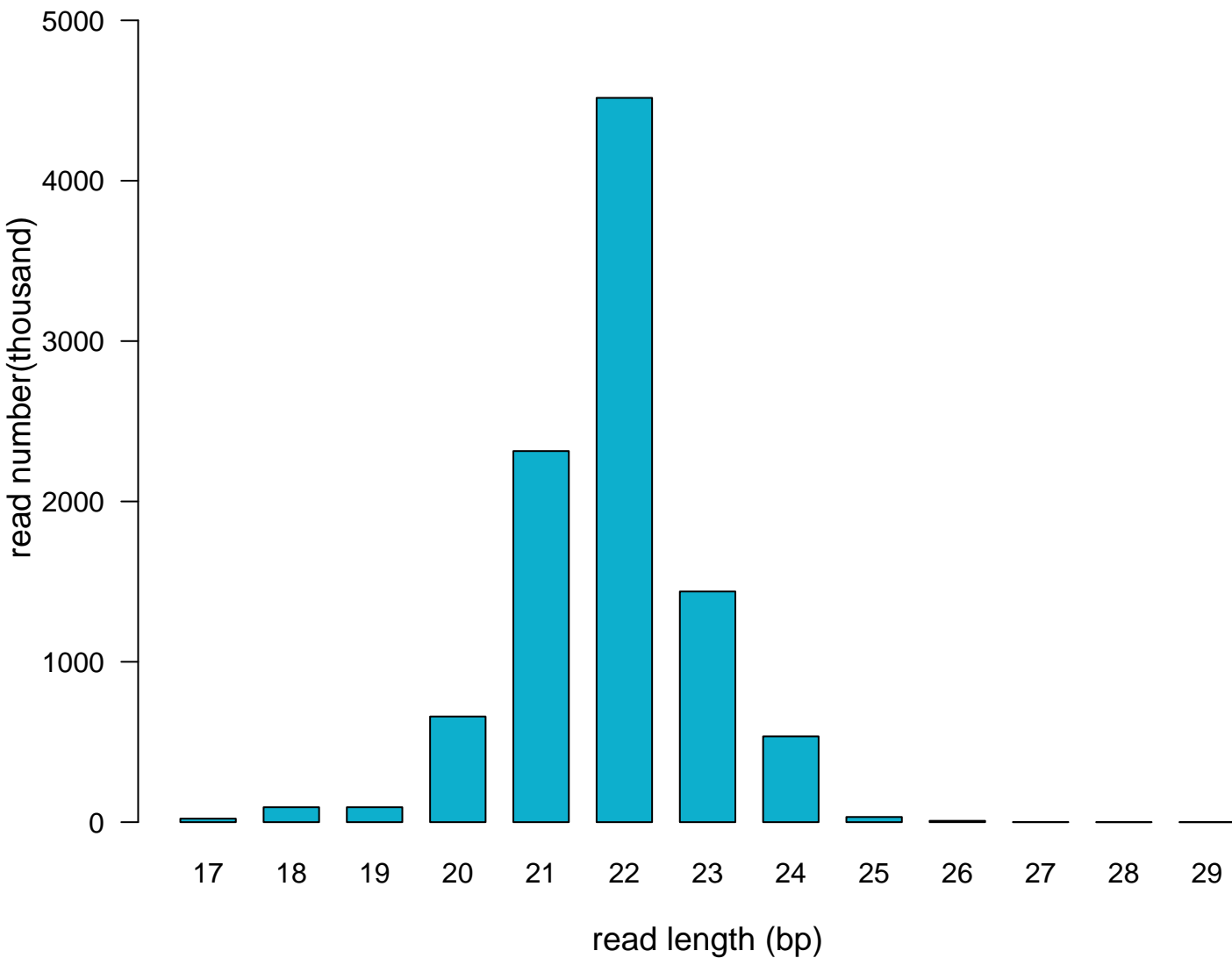

Supplement: Supplementary file 1 [file Presentation1.zip › Data/miRNA/know_miRNA_expression/5/5_miRNA_length.pdf]

# sRNA nucleotide bias at each position

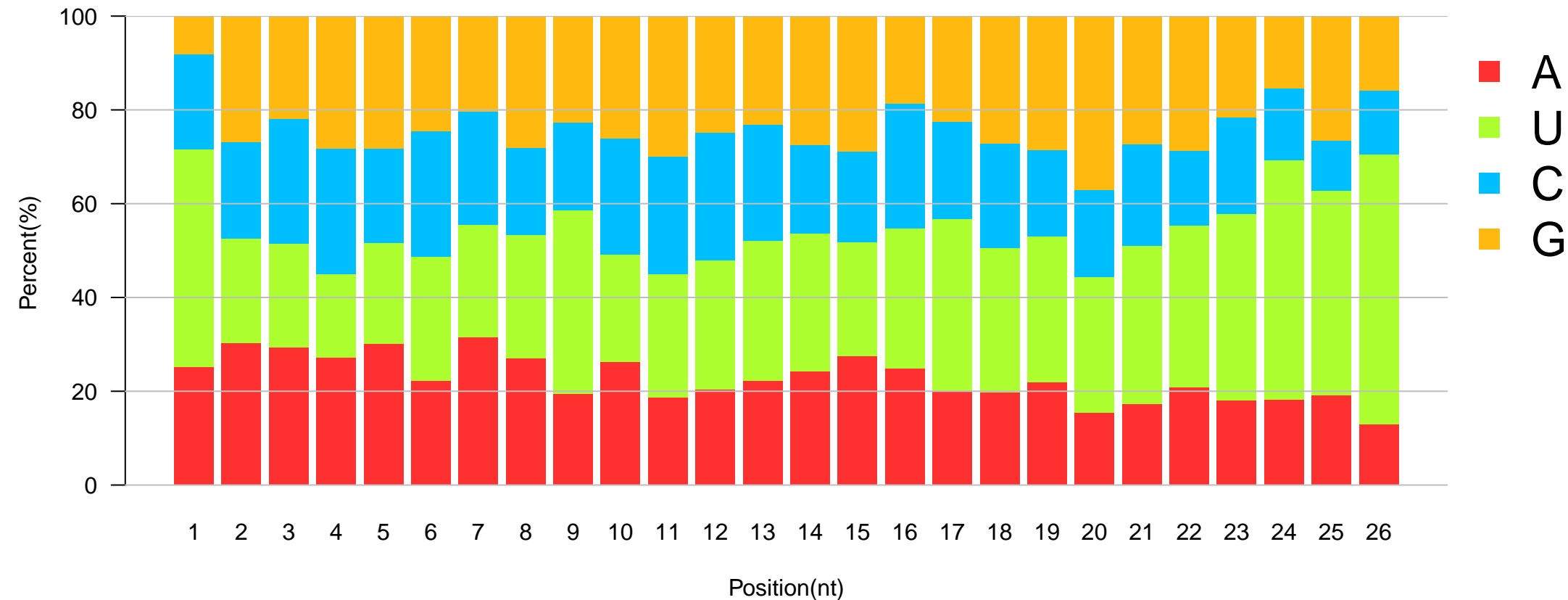

Supplement: Supplementary file 1 [file Presentation1.zip › Data/miRNA/know_miRNA_expression/6/6_base_preference.pdf]

**Read Length Distribution**

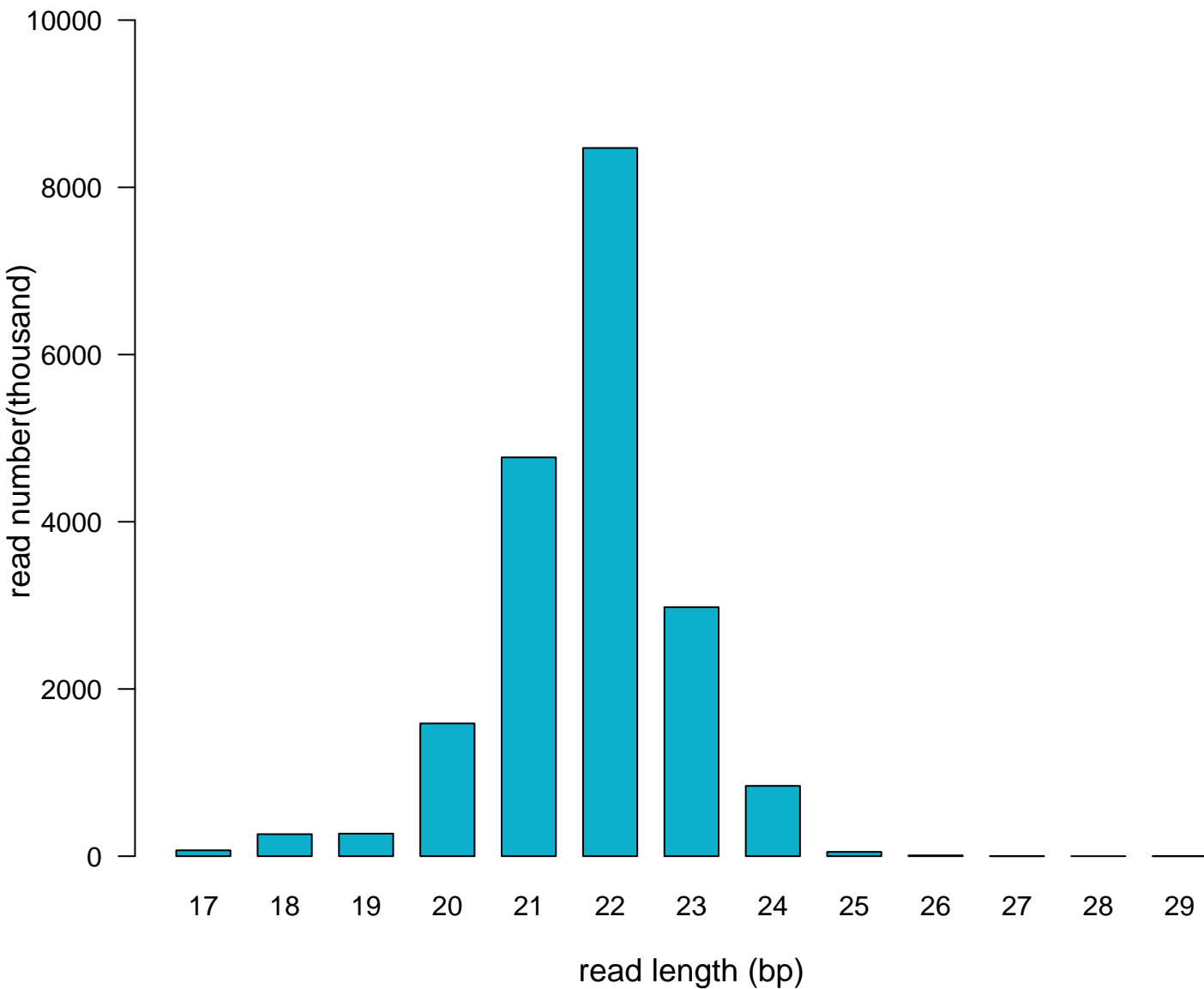

Supplement: Supplementary file 1 [file Presentation1.zip › Data/miRNA/know_miRNA_expression/6/6_miRNA_length.pdf]

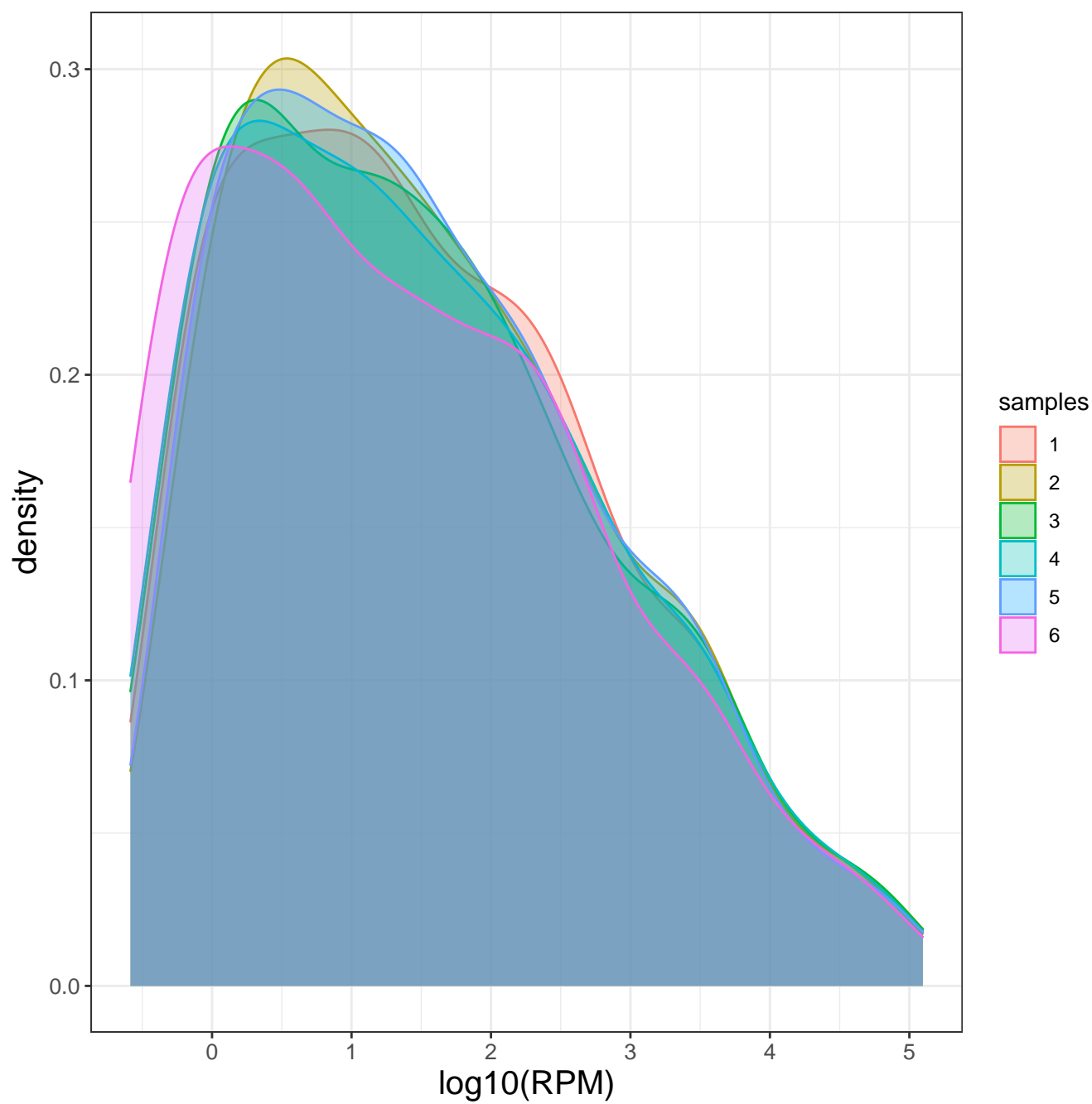

Supplement: Supplementary file 1 [file Presentation1.zip › Data/miRNA/know_miRNA_expression/density.pdf]

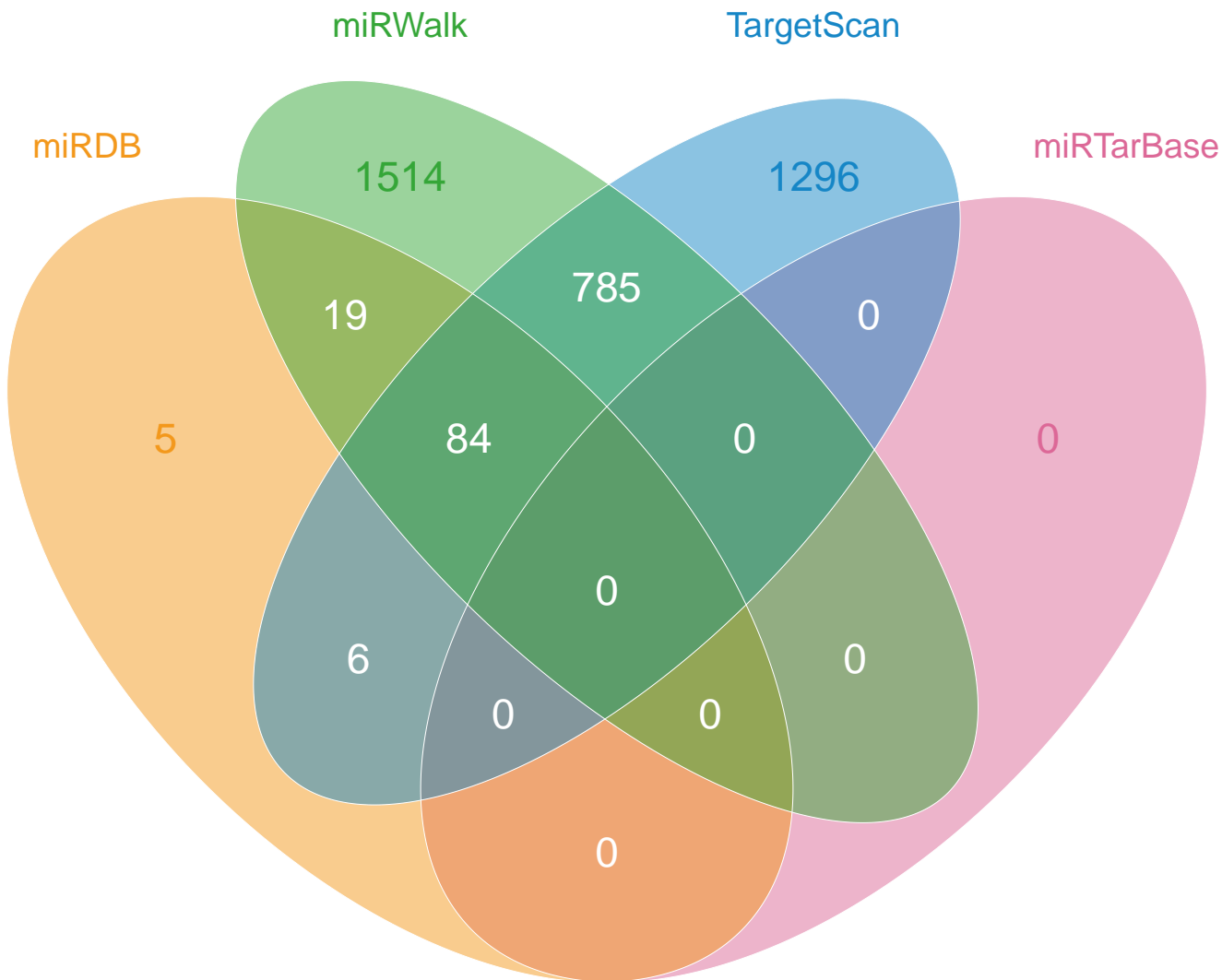

Supplement: Supplementary file 1 [file Presentation1.zip › Data/miRNA/miR_target/Control--Treatment/result/rno-miR-155-3p/rno-miR-155-3p.pdf]

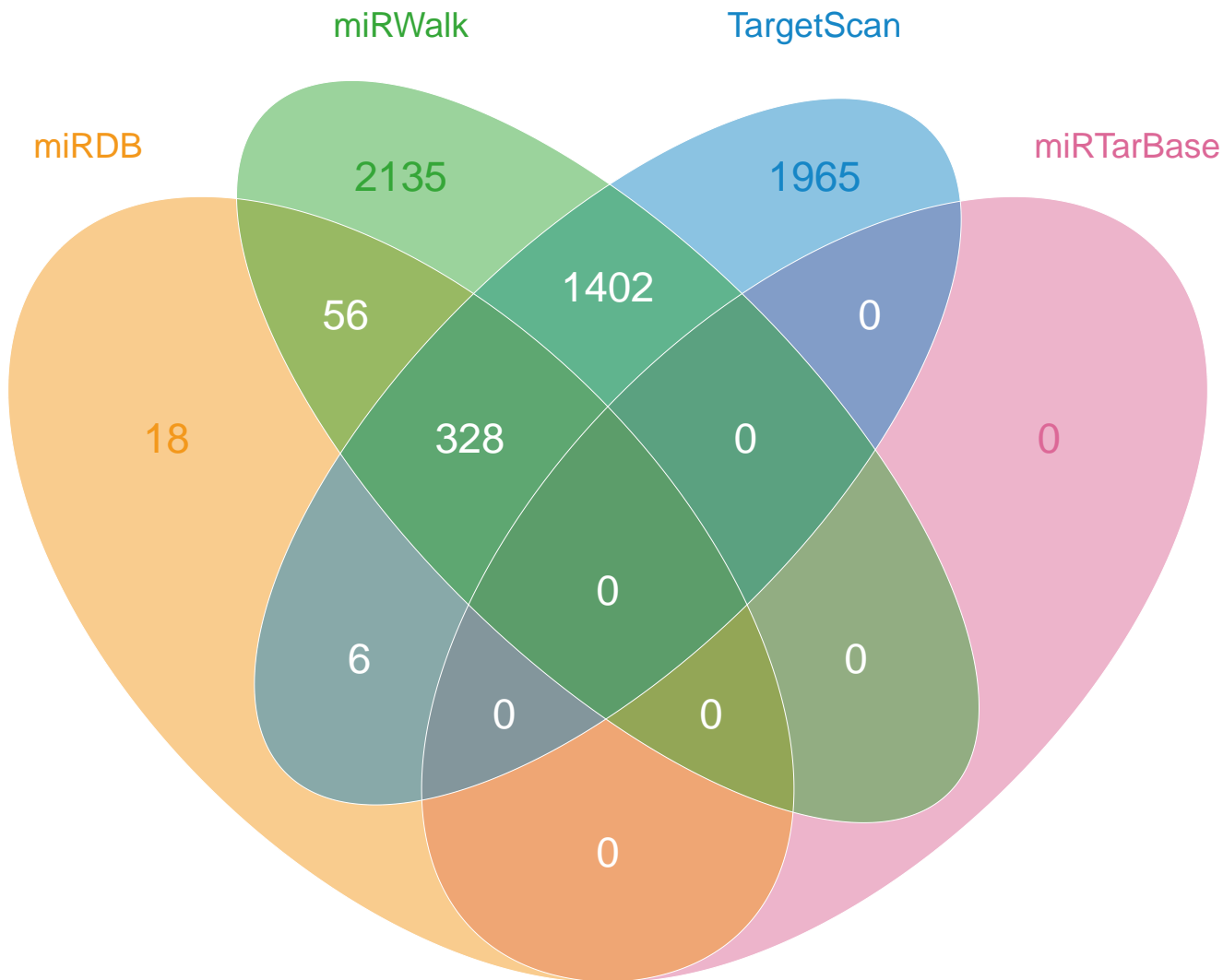

Supplement: Supplementary file 1 [file Presentation1.zip › Data/miRNA/miR_target/Control--Treatment/result/rno-miR-21-3p/rno-miR-21-3p.pdf]

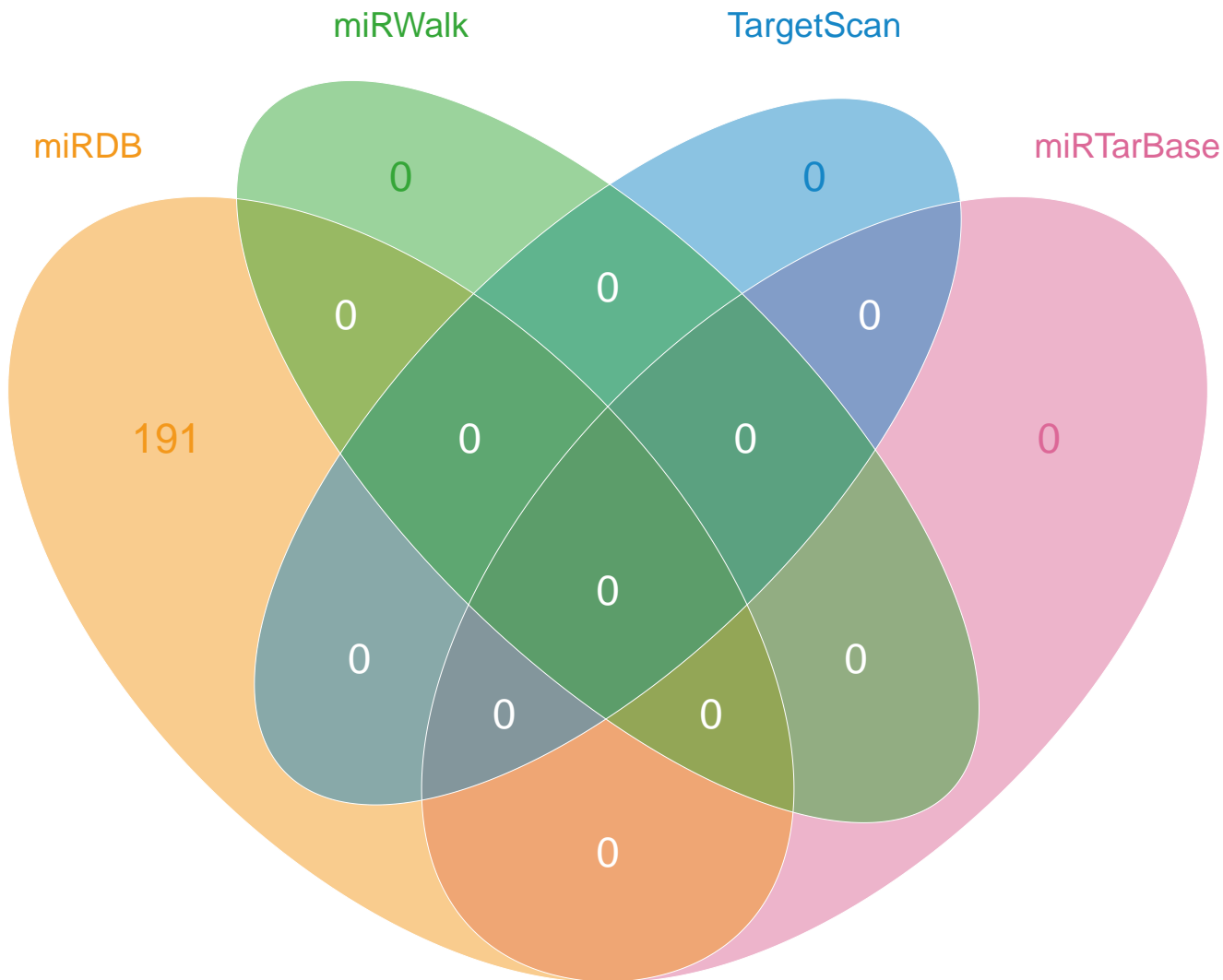

Supplement: Supplementary file 1 [file Presentation1.zip › Data/miRNA/miR_target/Control--Treatment/result/rno-miR-3084a-1-5p/rno-miR-3084a-1-5p.pdf]

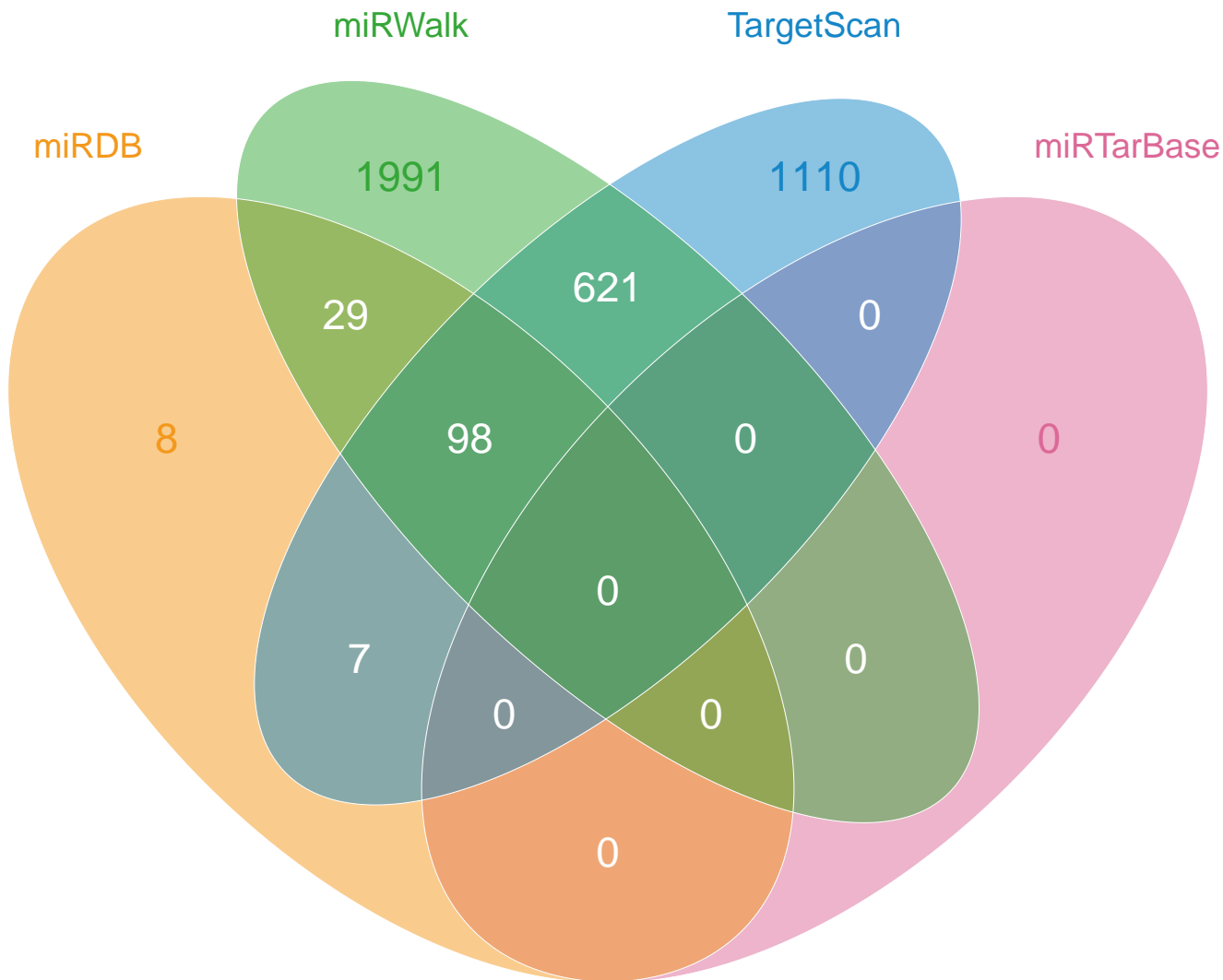

Supplement: Supplementary file 1 [file Presentation1.zip › Data/miRNA/miR_target/Control--Treatment/result/rno-miR-380-3p/rno-miR-380-3p.pdf]

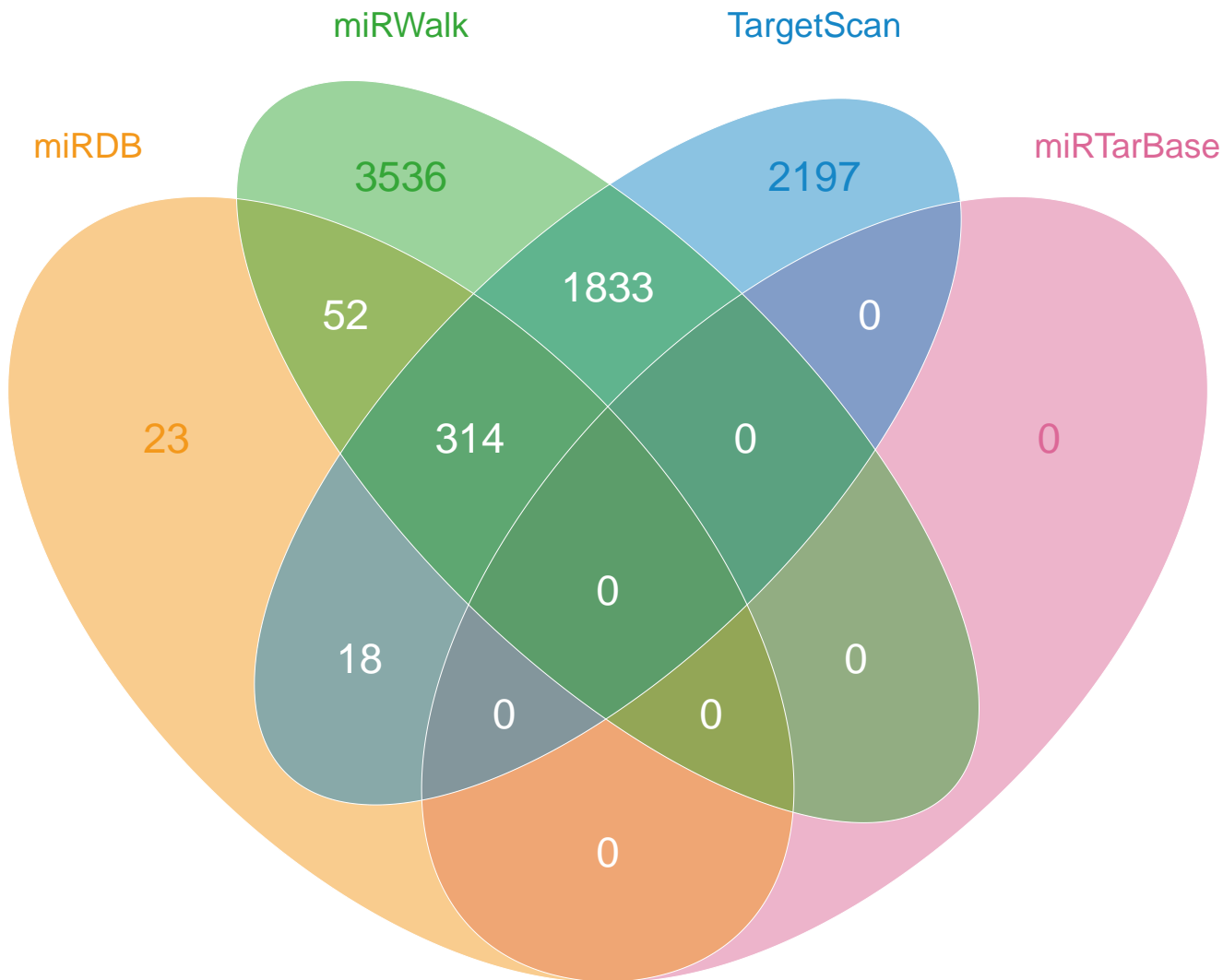

Supplement: Supplementary file 1 [file Presentation1.zip › Data/miRNA/miR_target/Control--Treatment/result/rno-miR-539-5p/rno-miR-539-5p.pdf]

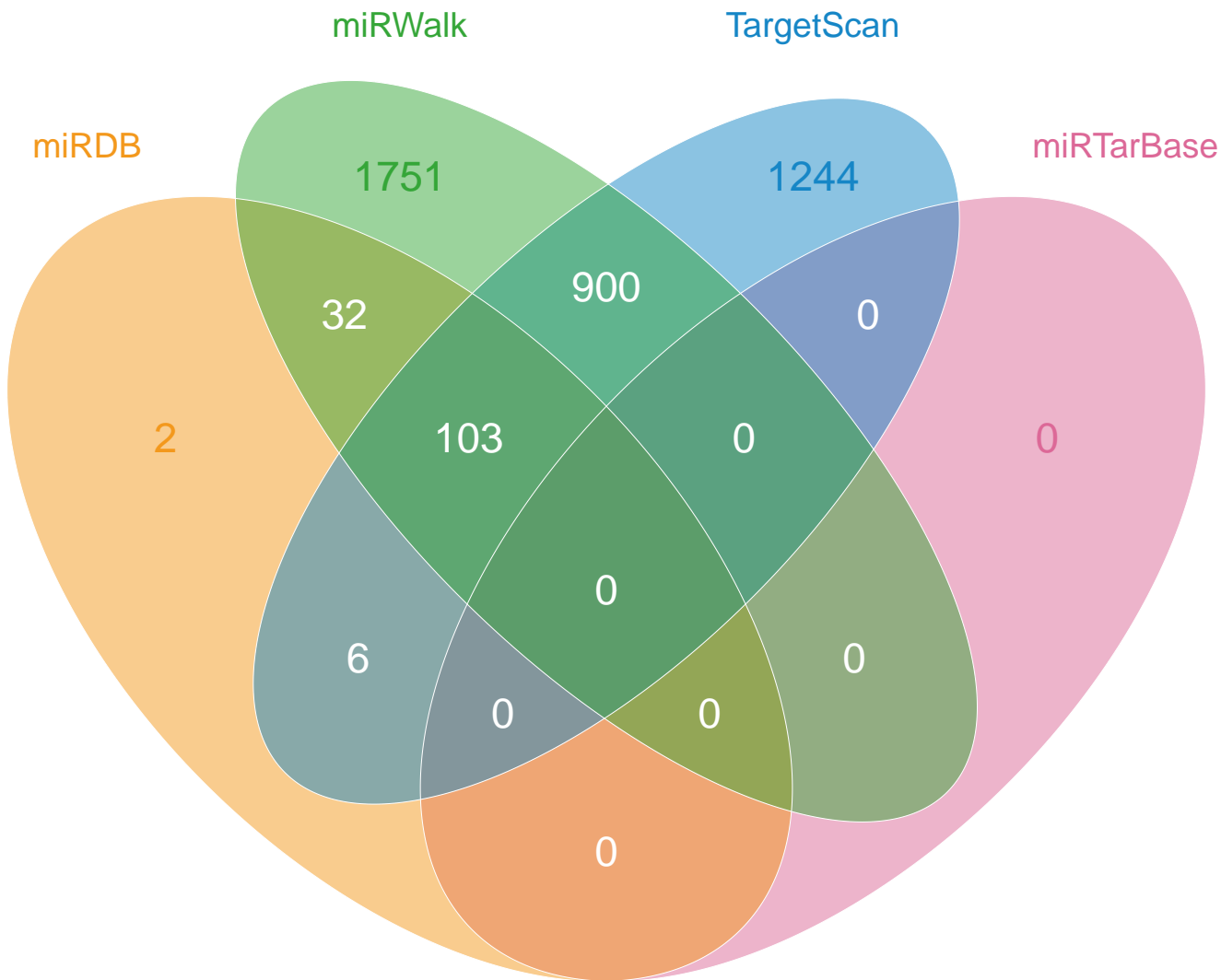

Supplement: Supplementary file 1 [file Presentation1.zip › Data/miRNA/miR_target/Control--Treatment/result/rno-miR-667-3p/rno-miR-667-3p.pdf]

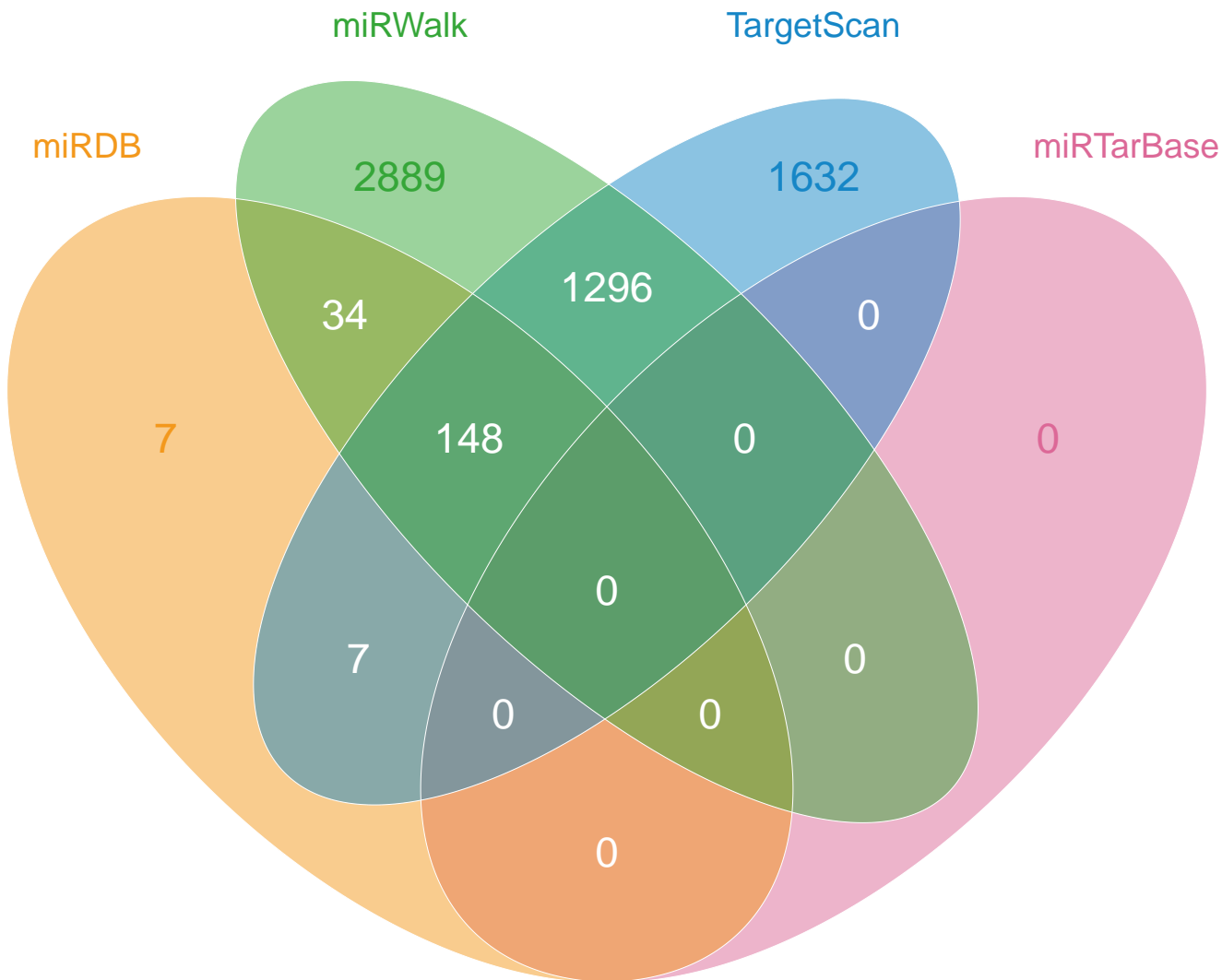

Supplement: Supplementary file 1 [file Presentation1.zip › Data/miRNA/miR_target/Control--Treatment/result/rno-miR-879-5p/rno-miR-879-5p.pdf]
